# AI-imputed and crowdsourced price data show strong agreement with traditional price surveys in data-scarce environments

**DOI:** 10.1371/journal.pone.0320720

**Published:** 2025-04-08

**Authors:** Julius Adewopo, Bo Pieter Johannes Andrée, Helen Peter, Gloria Solano-Hermosilla, Fabio Micale

**Affiliations:** 1 Development Data Group, World Bank, Washington, DC, United States of America; 2 International Institute of Tropical Agriculture – IITA, Ibadan, Nigeria; 3 Universidad Pablo de Olavide, Sevilla, Spain; 4 European Commission Joint Research Center (EC-JRC), Ispra, Italy; United Arab Emirates University Faculty of Science: UAE University College of Science, UNITED ARAB EMIRATES

## Abstract

Continuous access to up-to-date food price data is crucial for monitoring food security and responding swiftly to emerging risks. However, in many food-insecure countries, price data is often delayed, lacks spatial detail, or is unavailable during crises when markets may become inaccessible, and rising prices can rapidly exacerbate hunger. Recent innovations, such as AI-driven data imputation and crowdsourcing, present new opportunities to generate continuous, localized price data. This paper evaluates the reliability of these approaches by comparing them to traditional enumerator-led data collection in northern Nigeria, a region affected by conflict, food insecurity, and data scarcity. The analysis examines crowdsourced prices for two staple food commodities, maize and rice, submitted daily by volunteers through a smartphone application over 36 months (2019–2021), and compares them with data collected concurrently by trained enumerators during the final eight months of 2021. Additionally, the crowdsourced dataset is compared to AI-imputed prices from the World Bank’s Real-Time Prices (RTP) database. Data from the alternative methods reflected similar price inflation trends during the COVID-19 pandemic. Pearson’s correlation coefficients indicate strong statistical agreement between crowdsourced and enumerator-collected prices (*r* =  0.94 for yellow and white maize, *r* =  0.96 for Indian rice, and *r* =  0.78 for Thailand rice). Furthermore, the crowdsourced data shows a high correlation with the AI-imputed prices (*r* =  0.99 for maize, and *r* =  0.94 for rice). The results from additional statistical tests of normality and paired means shows that the discrepancies between price datasets are consistent with measurement error rather than differences in actual price dynamics. Further tests of equivalence confirmed that enumerator and crowdsourced prices represent the same underlying market processes for specific commodity subtypes, and connotes that crowdsourced price data is a credible reference for validating AI-imputed estimates. The results support the use of AI imputation and crowdsourcing methods to improve price data collection and track market dynamics in near real time. These data innovations can be particularly valuable in areas that are underrepresented in national aggregate data due to limited monitoring capacity, and where high-frequency local data can aid targeted interventions.

## Introduction

Food and consumer price inflation directly affects household food affordability and serves as a leading indicator of food insecurity [1–3]. Recent trends in food commodity price inflation have severely undermined food affordability and security for millions of households, posing a significant threat to development across low- and middle-income countries (LMICs) [4–6]. Monitoring commodity prices and food price inflation is essential for early warning systems and rapid assessments of risks to markets and food security [7–9].

Food prices typically reflect market conditions and capture responses to economic shocks and threats. However, despite significant investments in national and regional price monitoring systems, tracking high-frequency changes in food prices at individual markets remains a major challenge in many LMICs [10]. These challenges include long planning time, high costs, and logistical constraints, which limit the coverage, timeliness, and granularity of collected data [11,12]. Despite these challenges, governmental institutions, development agencies, and market stakeholders depend on such data to inform decisions related to economic development, humanitarian interventions, and agricultural investments [13].

In many countries, commodity price data used for national statistics are gathered through snapshot surveys or opaque proxy methods conducted by designated agencies or third-party institutions [14,15]. Effective food security preparedness plans and timely interventions require robust data and credible insights for targeted action and real-time impact assessment. At best, current official data provide a delayed view of food security status and changes; at worst, they misrepresent or under-represent critical market conditions, undermining effective decision-making.

To address the food price data gap, researchers have proposed augmenting official price data systems with alternative data collection methods using different innovative approaches [12,16,17]. Two particularly promising methods for improving the timely monitoring of market prices are phone-based data crowdsourcing, enabled by the widespread availability of mobile phones, and real-time data imputation, driven by rapid advancements in artificial intelligence (AI) techniques. Two examples of these methods include the European Commission’s Joint Research Center (JRC) crowdsourcing initiative in northern Nigeria [18] and the World Bank’s AI-driven Real-Time Prices (RTP) dataset [8,19,20].

In 2019, the Food Price Crowdsourcing in Africa (FPCA) initiative was launched by the JRC and collaborating institutions to curate commodity prices through volunteer submissions, beginning with a pilot implementation in northern Nigeria [21–23]. The crowdsourcing approach leverages “citizen science” principles, engaging volunteers (paid or unpaid) to submit data at regular or irregular intervals, which can then be aggregated across locations and time [24]. This method assumes that the diversity of volunteer contributors yields rich, minimally biased data, as submissions are decentralized and independent, offering a robust quasi-sampling of the population at scale [22,23]. Beyond its cost-effectiveness and timeliness, crowdsourcing fosters an inclusive digital and data ecosystem, empowering citizens as active data curators and stakeholders in food systems data.

An example of AI-augmented price data is the RTP database by the World Bank [19,20,25]. It leverages imputation [8,26] to generate continuous data for over 2,200 markets in 36 countries covering food prices, energy prices, and unofficial parallel market exchange rates. This data is automatically derived from intermittent surveys conducted by organizations such as the Food and Agriculture Organization (FAO) and the World Food Programme (WFP) [27]. While AI applications for complex analyses are not new, the integration of modern models with real-time data pipelines is enabling advanced secondary capabilities [9,28–31] that have the potential to transform food security risk monitoring at both local and global levels.

Given the rapid adoption of these emerging data innovations, it is crucial to evaluate whether these new data sources accurately reflect market signals and actual price levels. Both crowdsourcing and AI-driven imputation have inherent limitations. For instance, crowdsourcing can suffer from inconsistencies in volunteer submissions, such as outdated or spurious price data, which can compromise data integrity despite built-in controls [22,23]. Similarly, AI-generated price estimates can propagate biases contained in input data and be affected by accuracy issues and uncertainties stemming from model testing and design strategies [8,32]. Therefore, validating price data produced through these alternative approaches against independent data collected by trained enumerators is critical to advance their use in real-time food price assessments.

To address this important need, this study aims to assess the reliability of phone-based data crowdsourcing and AI-driven real-time imputation as alternative methods for collecting food price data. We conducted a correlative comparison of crowdsourced food prices with the World Bank’s monthly AI-generated prices over three years (2019–2021) in northern Nigeria, a region affected by conflict, food insecurity, and data scarcity. Using enumerator-collected price data as a benchmark during the final eight months of 2021, we first evaluated whether crowdsourced data accurately reflected market signals and actual price levels at the sub-national level. After anchoring the crowdsourced data to this ground-truth, we investigated the alignment between the crowdsourced and AI-imputed data over the full three-year period. In addition to validating these novel datasets, this study aims to inspire future validation efforts of related initiatives and highlight the operational readiness of AI imputation and crowdsourcing as viable options to monitor prices in data-scarce environments.

Our findings revealed a strong statistical agreement between the enumerator-collected, crowdsourced, and AI-imputed data. Pearson’s correlation coefficients indicated high levels of agreement (*r* ranging from 0.78–0.99) for monthly prices, and additional formal statistical tests suggest that observed discrepancies between datasets are consistent with measurement error rather than differences in actual price dynamics. These validation results are significant because they demonstrate that crowdsourcing and AI imputation can provide timely, localized, and accurate food price information, especially in regions where traditional data collection is challenging. By confirming the efficacy of these methods, this study contributes to closing the food price data gap and supports more reliable monitoring of food security risks, enabling timely decisions and more targeted interventions in LMICs.

## Materials and methods

### Study area

**Fig 1:**
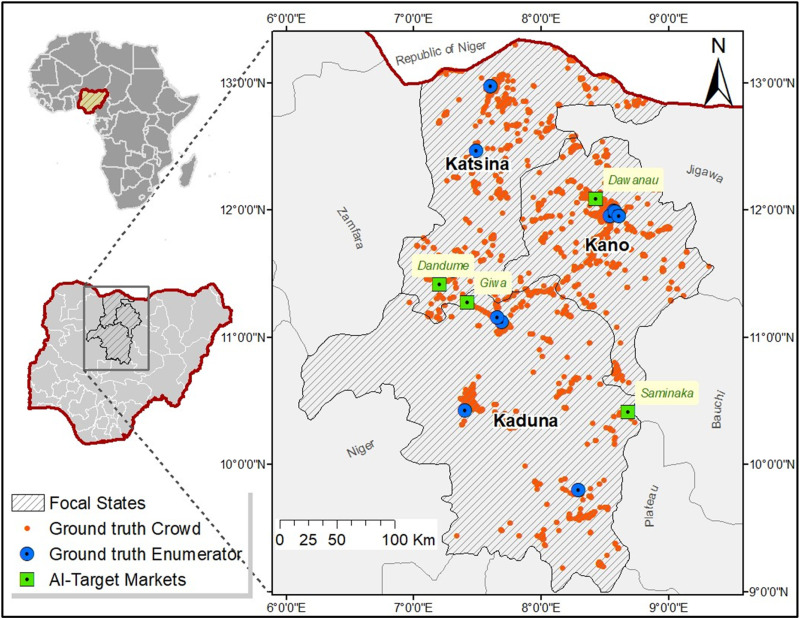
A map of the study area in the core northern region of Nigeria, illustrating georeferenced market locations where ground-truth commodity prices were observed by volunteer crowds and trained enumerators. The map also highlights market locations where artificial intelligence imputation models were employed to estimate prices. The administrative boundary outlines are from GADM, provided under a free license provision (www.gadm.org).

This study was conducted in the core northern region of Nigeria, encompassing three major states (Fig 1). These states collectively have a population of 30.5 million people, representing 15% of the national population [33] that produce 6.5% of the national GDP [34]. The region is characterized by low literacy levels and a history of insecurity, with persistent threats to food security. These conditions underscore the need for alternative approaches to monitoring food commodity prices to support timely and targeted interventions.

The major markets in the region operate year-round, varying in size and typically featuring a diverse range of vendors, from wholesalers to retailers. Smaller markets, such as village markets near farm gates, open periodically, while major cities host small and mid-sized retailers, including kiosks, shops, and supermarkets. Active trade routes facilitate the movement of people and goods between markets, and vendors frequently manage multiple stores within and across markets.

The study area was selected based on specific criteria for validating alternative data sources, including matched coverage areas between the data sources, overlap of commodities, and a clear need for data innovation to address food security challenges and economic monitoring. Prior to the crowdsourcing efforts, the region was under severe warnings due to insecurity stemming from terrorism, armed banditry, and religious conflict. These factors heightened food security and development risks while exacerbating data scarcity. These challenges make the region an ideal setting to test whether crowdsourcing and AI imputation can generate reliable data under such difficult conditions.

It is important to note that both data innovation efforts were implemented independently of each other. The independent parallel implementation of these efforts provided a strong basis for validating the price data generated from both sources in comparison to traditional data collection methods.

### Data description

This analysis focuses on three food price datasets generated through different methods and published for public use via open-access repositories [8,22,26,35]. These datasets are:

(I) **Enumerator-submitted price dataset:** Commodity prices collected from various markets by trained enumerators within the focal geography during the final eight months of the FPCA pilot project (2019–2021) conducted by EC-JRC.
(II) **Crowdsourced price dataset:** Commodity prices reported by citizen volunteers from various markets over a 36-month period during the FPCA pilot project.
(III) **AI-imputed price dataset:** Retail commodity prices from the World Bank’s RTP database, imputed using artificial intelligence.

The enumerator-submitted price data serve as the ground truth for this study. By collecting data concurrently with the crowdsourced data during the final eight months of the FPCA project, we had the opportunity to evaluate the accuracy and reliability of the crowdsourced price data against the benchmark provided by trained enumerators. Once the reliability of the crowdsourced data was established, its extended time span and larger sample size enabled subsequent comparisons with the AI-imputed price data. This sequential approach broadened the scope of our validation, enabling us to assess the reliability of these innovative methods across different time scales and market segments. Details about how these datasets were generated, including processes, scope, and quality control, have been previously published elsewhere [8,22]. A brief description of each dataset is provided below.

### Enumerator-submitted price dataset

The FPCA enumerator-led price data were collected by twelve trained enumerators residing in the focal states who were randomly selected to collect and submit price data. These enumerators were drawn from a broader pool of agricultural extension workers and field research agents who were familiar with markets in their respective states and experienced in-field data collection. Before deployment, they received additional training that included basic instructions on best practices for obtaining price data from sellers, ensuring comprehensive coverage of major market segments, and adhering to expectations for the frequency and quality of price data submissions.

The trained enumerators visited market locations closest to their residences within their states and submitted observed or transacted commodity price data using the Open Data Kit (ODK) app. Due to their dispersed locations across the study area (Fig 1), they provided price data from at least 24 unique market locations, including small shops, kiosks, supermarkets, and large open markets, aligned with the three aforementioned market segments: Farm gate, Retail, and Wholesale.

Each enumerator was required to input a pre-assigned unique identifier (ID) when submitting data. These IDs differentiated records submitted by enumerators from those submitted by the volunteer crowd. Data contributors (both enumerators and volunteers) were not pre-assigned to specific markets, which meant that individuals could choose which markets to visit at any given time. While this might introduce occasional biases in market selection, it was deemed inconsequential to the overall outcomes due to the spatial distribution of contributors and the extended duration of data collection.

This approach contrasts with the more rigid, pre-assigned market visits typically employed by national statistical offices (NSOs), where enumerators are instructed to collect data from only a limited number of markets per state (often between one and five). Such constraints can result in a narrower view of price dynamics and leave NSOs vulnerable to criticisms of bias or “data manipulation,” as selected markets may not fully reflect the price variations experienced by the broader population. In contrast, allowing contributors the flexibility to choose markets provides a more comprehensive and representative perspective on where consumers purchase commodities and the prices they encounter.

Moreover, the diversity of unit packages reported by both the volunteer crowd and the enumerators mirrored the reality of local market transactions, where commodities are sold in a wide range of packaging sizes and measures.

### Crowdsourced price dataset

The FPCA crowdsourced price data were submitted by 1,182 volunteer citizens residing in the study area who had access to various market locations to report commodity prices. The volunteers were remotely invited and onboarded to submit commodity price data, including maize and rice, through the ODK app. To participate, volunteers needed access to a smartphone, demonstrate competency in using the data submission app, and indicate willingness to submit prices during market visits, whether casual or purposeful [21].

The initial data collection phase lasted for one year (January 2019 – January 2020), as originally planned for the project. However, due to the onset of the COVID-19 pandemic, this remote data collection approach was reinstated to monitor price trends market disruptions caused by lockdowns and other shocks. The submission portal was re-opened from May 7, 2020, to September 16, 2020, during which volunteers continued to submit price data. This re-engagement validated the assumption regarding the volunteers’ willingness to continue the task. A subsequent wave of data submission was launched from March 6, 2021, to December 31, 2021.

The participating volunteers submitted georeferenced price data from over 150 unique market locations, classified into three key market segments: Farm gate, Retail, and Wholesale [22,23]. The geographic dispersion of the volunteers and their independent daily submissions enabled the collection of multiple price data points within each day (“intraday prices”), which were averaged into regular time intervals such as daily, weekly, and monthly. The volume of price submissions across diverse market locations peaked at 326 data records per day. To ensure data quality, daily checks were conducted to confirm that submitted prices fell within realistic ranges for the commodities, relative to data from other volunteers in the region, as previously documented [22].

### AI-imputed price dataset

Monthly AI-imputed prices (version dated September 26, 2023) were accessed from the World Bank’s Real-Time Prices (RTP) database [25], which covers 10 price series across 53 market locations in Nigeria from January 2007 onward. The dataset is updated weekly using a Markov Chain Monte Carlo framework that incorporates an ensemble of models to address missing data points in surveys provided by WFP in partnership with the Humanitarian Data Exchange (HDX) [8,19,27].

The source surveys provide a wealth of information on prices, but any analysis of continuous trends is complicated by outliers, incorrect price entries, and the overall sparse, incomplete, and intermittent nature of the data. To overcome this, each missing price data point is imputed using information from price surveys gathered in nearby locations or related commodities. The imputation process employs hybrid machine learning models that integrate local relationships within specific regions of the feature space, akin to the concept of local receptive fields in convolutional neural networks [8]. These models rely on fast tree-based approaches to further enhance computational efficiency. Input surveys are adjusted using non-parametric density estimation and other techniques, resulting in an outlier-resistant and fault-tolerant price monitoring system capable of handling misplaced digits, incorrect price entries, and missing data in the underlying surveys. This ongoing effort by the World Bank aims to support the analysis of local price dynamics in markets where prices are highly sensitive to localized shocks and where traditional data collection methods are unavailable.

The AI-imputed monthly data are presented as Open, High, Low, and Close (OHLC) price estimates to capture intra-month variation, while the crowdsourced and enumerator-collected price data were gathered daily. With each update of the AI-imputed dataset, the World Bank conducts a model-based internal accuracy assessment using cross-validation techniques. These assessments compare imputations against observed prices and calculate an outlier-robust pseudo *r*-squared, based on a normalized mean-absolute error metric. Accuracy statistics are and updated weekly for each price item and published along with the dataset. For the version of the data used in this analysis, reported accuracy ranged from 87% to 97%, indicating a high level of confidence in the modeled price estimates [25]. Prices are generated for 53 markets, of which four markets matched directly to locations covered by the FPCA project.

### Data pre-processing

The enumerator and crowdsourced prices were reported in various local measurement standards (such as *derica*, *kongo*, *mudu*) or packaging sizes (5 kg bags, 10 kg bags, 25kgs bags and others) as previously reported [22]. In contrast, the AI-imputed prices were exclusively based on 100 kg bags for maize and 50 kg bags for imported rice. To compare commodity prices across all three datasets, the price per unit (₦/kg) was calculated by dividing the reported price by the corresponding weight for each package or by the standard weight associated with the local measurement unit for each commodity, following established methods [22]. Note that the conversion does not control for possible discounts associated with larger packaging sizes. The potential effects of bulk-discounted prices are deemed to be minor (12% of the data record) because they are randomly within the reported quantities and showed no impact on aggregated price trends.

The first step of our analysis involved evaluating the relationship between daily crowdsourced prices and enumerator-submitted prices collected from markets. Next, average prices were computed to align the data across uniform time intervals, enabling pairwise correlations between the datasets. Although the AI-imputed data include OHLC prices, we focused on the closing prices, as they best represent prevailing market conditions at a fixed moment within each month. Data aggregation and averaging were performed at administrative and market segment levels, consistent with the general approach for price reporting in the country, to minimize the risk of misrepresenting prices from individual submissions or isolated markets.

The raw intraday crowdsourced and enumerator-submitted prices were screened for outliers using the Tukey method [35,36], which calculates the interquartile range and applies a fence rule to identify and exclude extreme values. This process was further refined by expert knowledge of realistic price ranges for each commodity during the study period. In total, 1.3% of the crowdsourced data were excluded, with negligible impact on the calculated averages or correlations. The remaining intraday prices were aggregated into daily, weekly, and monthly averages for each commodity.

### Correlation analysis

We focused our analysis on the prices of maize and rice, as these commodities were common across all three datasets. Both are staple foods widely consumed and traded in the region and nationally, making them ideal for the validation analysis. All statistical analyses were implemented using R software. The entire code and data are available in the data and materials section of this paper. Also, the metadata and documentation have been published as an open-access reproducible package at https://reproducibility.worldbank.org/index.php/catalog/137.

To evaluate the consistency among the three data sources, we conducted Pearson’s pairwise correlation analysis, and interpreted the correlation coefficient as a measure of the linear association between two variables. This approach follows methodologies described in previous studies [37,38]. The primary goal was to determine whether the crowdsourced price data were strongly correlated with the enumerator-submitted prices during their eight-month overlap, and whether the AI-imputed prices showed a strong correlation with the crowdsourced data over the full 36-month period. Establishing strong correlations in both cases would suggest that the alternative data sources accurately reflect comparable market price dynamics over time and are reliable alternatives.

The correlation coefficient (*r* ) and the coefficient of determination ($r^{2}$) were the key metrics used to assess the reliability of the alternative datasets. We calculated these as follows:

**Correlation Coefficient (** *r* **):**


$$r=\frac{n\sum XY-\left(\sum X\right)\left(\sum Y\right)}{\sqrt{\left[n\sum X^{2}-{\left(\sum X\right)}^{2}\right]\left[n\sum Y^{2}-{\left(\sum Y\right)}^{2}\right]}}$$ (1)

**Coefficient of Determination (** $r^{2}$ **):**


$$r^{2}={\left(r\right)}^{2}\;,\left(\text{trivially}\right)\text{.}$$ (2)

Where:

- *n* is the number of observations,
- *X* represents the prices from data source 1,
- *Y* represents the prices from data source 2,

**Correlation Coefficient (***r*): Measures the direction and strength of the linear relationship between two variables, in this case, prices from two different data sources. The value of *r* ranges from -1 to + 1:

- r=+1: Perfect positive linear relationship.
- r=0: No linear relationship.
- r=−1: Perfect negative linear relationship.

A high positive *r* indicates that as prices from one source increase, prices from the other source also increase proportionally.

**Coefficient of Determination (**$r^{2}$): Represents the proportion of variance in one variable that is explained by the variance in the other variable. It ranges from 0 to 1:

- $r^{2}=1$: Perfect fit; the observations in one price measurement explain all the variability of the second price variable around its mean.
- $r^{2}=0$: The price measurement explains none of the variability in the other price measurement.

When $r^{2}$ value is close to 1, it suggests that a large proportion of the variance in one dataset is explained by the other dataset.

These metrics are particularly useful in our validation exercise because they provide quantitative measures of how well the different data sources replicate patterns observed in the validation data. Specifically, the correlation metrics measure the co-movement of alternative measurements of the same underlying price process, and the degree of correspondence is not affected by constant factors such as fixed mark-ups, differences in transport costs, or fixed differences in per-unit prices related to packaging sizes.

### Assumptions and tests

The use of *r* and $r^{2}$ assumes only a linear relationship between the price variables, which is reasonable for our data because the analysis focuses on different independent measurements of the same underlying price processes. The calculation of the correlation coefficient does not require further statistical assumptions, but several aspects are worth noting.

While the correlation coefficient measures the strength and direction of a linear relationship between two variables, it is not directly a measure of independence. If two variables *X* and *Y* are independent, then their correlation coefficient is zero because independence implies that *X* provides no information about *Y*, and vice versa, resulting in no linear relationship between them. However, a zero correlation does not imply independence unless *X* and *Y* are jointly normally distributed, as non-linear or more complex relationships can still exist between the variables. Hence, while the linearity assumption is trivially satisfied in our case, it is crucial.

Independence of datapoints still plays an important role in our study, particularly in the context of measurement errors. Suppose *X* is a measurement of x+e and *Y* is a measurement of y+v, where *x* and *y* are the true price processes with *e* and *v* as additional measurement errors. If there is a correlation between the measurement errors *e* and *v*, a correlation between *X* and *Y* may be observed even when *x* and *y* are independent. In our setting, the data points were collected independently over time and across different locations. This allows us to assume that there is no correlation between measurement errors, so that the calculated correlation coefficients are a measure of the co-movement between the underlying price processes and not of the measurement processes. Hence, a high correlation indicates that the underlying price process tracked by the different measurements are related and likely identical, and that measurement errors must be small. While it is not a formal test, it provides an important metric to conclude on the reliability of the measurement processes.

Strict normality is not required for this correlation analysis itself when the focus is on the metric as a measure of co-movement. Normality is only required for further inference, such as constructing confidence intervals around the correlations or conducting hypothesis tests. However, outliers can impact correlation results, which is why basic outlier treatment was applied to the data (note that the AI-imputed data is inherently filtered from outliers, and no further treatment was needed).

### Sensitivity checks

If one price source consistently lags another, for instance because enumerators visit leading or lagging locations, or because AI imputations lag actual price developments, this leads to lower correlations, notwithstanding the correctness of both price observations or estimates. Therefore, we also examined if there was a temporal lag in the AI-imputed price trends compared to ground observations. Understanding time-lagged relationships is important for assessing whether the AI-imputation models estimate current prices accurately or if they misrepresent past or future prices, which could limit their usefulness for real-time monitoring. By analyzing potential time lags, we aimed to ensure that the AI-imputed data could effectively serve as timely indicators of market prices.

To further assess the locational accuracy of the AI-imputed prices, we also evaluated the correlation of the price datasets within lower administrative boundaries (Admin2 level). We calculated the centroid of each Admin2 boundary and selected the closest AI-imputation market location. Thereafter, we computed the average monthly crowdsourced prices for each Admin2 boundary using all georeferenced data within each unit. A final correlation test was conducted between the monthly AI-generated price data (nearest to the centroid) and the crowdsourced prices within each Admin2 boundary over the 36-month period. This analysis aimed to test the assumption that prices should correlate more closely with nearby AI-imputation market locations than with more distant ones, thereby assessing the spatial reliability of the AI-imputed data.

### Formal equivalence tests

While the correlation coefficients *r* and $r^{2}$ indicate the direction and strength of relationships between datasets, they do not assess whether the average prices are strictly equivalent across data sources. Strong correlations may arise between the prices of different goods due to shared inflationary dynamics or input-output linkages; however, such correlations do not guarantee that the price data reflect the same underlying goods. To address this limitation, we conducted additional formal tests to determine whether the price data differed significantly between the data sources in terms of their distributions during the study period [35]. These tests impose stricter criteria for evaluating dataset equivalence, allowing us to establish equivalence or identify differences regardless of the presence of strong correlations between distinct price series.

### Null hypothesis: Identical price processes and measurement errors

Consider the example of comparing the AI-imputed price data against the crowdsourced price data: *X* representing the former, and *Y* representing the latter. These measurements can be expressed as:


X=x+e (3)

Y=y+v (4)

where *x* and *y* are the true underlying price processes, and *e* and *v* are independent measurement errors, each normally distributed with mean zero:


$$e∼N\left(0,\sigma_{e}^{2}\right),\;v∼N\left(0,\sigma_{v}^{2}\right)$$ (5)

Under the assumption that both datasets track the same underlying price process, as *x* and *y* converge (i.e., x−y→0), the difference between the measurements X−Y should tend toward a normal distribution centered at zero, with variance equal to the sum of the measurement errors. Specifically, the difference between the measurements is:


$$D=X-Y=\left(x+e\right)-\left(y+v\right)=\left(x-y\right)+\left(e-v\right)$$ (6)

Thus, the expectation of *D* is:


$$E\left[D\right]=E\left[X-Y\right]=E\left[x-y\right]+E\left[e-v\right]=E\left[x-y\right]+0=E\left[x-y\right]$$ (7)

Since *e* and *v* have zero mean. This shows that $E\left[D\right]=0$ when both price processes captured by the two datasets are identical, or $E\left[D\right]=\tau$ if $E\left[x\right]=E\left[y\right]+\tau$, where *τ* represents a fixed price markup, such as transport costs or other fixed price differences between two measurement locations selling the same goods.

The variance of *D* is:


$$\text{Var}\left(D\right)=\text{Var}\left(X-Y\right)=\text{Var}\left(x-y\right)+\text{Var}\left(e-v\right)=0+\left(\sigma_{e}^{2}+\sigma_{v}^{2}\right)=\sigma_{e}^{2}+\sigma_{v}^{2}$$ (8)

Thus, the distribution of *D* is:


$$D=X-Y∼N\left(x-y,\sigma_{e}^{2}+\sigma_{v}^{2}\right)$$ (9)

This means that as the underlying processes converge to a fixed markup x−y→τ, the distribution of *D* becomes:


$$D=X-Y∼N\left(\tau ,\sigma_{e}^{2}+\sigma_{v}^{2}\right)$$ (10)

Moreover, if no markup exists, the difference between *X* and *Y* approximates the difference between the measurement errors:


$$D=e-v∼N\left(0,\sigma_{e}^{2}+\sigma_{v}^{2}\right)$$ (11)

### Alternative hypothesis: Diverging price processes and non-normal innovations

While the difference sequence $D_{t}=x_{t}-y_{t}$ should be approximately normal when the two datasets track the same prices and differ only due to normal measurement errors, it is crucial to consider alternative scenarios where the price processes diverge or exhibit more complex dynamics.

In practice, price data innovations to track changes or fluctuations in price levels often do not follow a normal distribution. Prices can exhibit skewness and kurtosis due to economic shocks, market imperfections, or structural breaks in exchange rates and price levels. Such non-normality is common in spatial time series [39] and is characteristic of financial and commodity price series, which may display long tails and asymmetries in their distributions. Our data visually confirms this notion, especially during periods like the COVID-19 pandemic when prices rose sharply over a short time span. We shall also provide test statistics that reject normality of the price data formally.

When $x_{t}$ and $y_{t}$ are different (non-normal) price processes, the assumptions underlying the normality of $D_{t}$ are unlikely to hold. For clarity, we will consider the case in which they are random walks driven by independent innovations from distributions with skewness and kurtosis, sharing a common drift term that induces correlation between $x_{t}$ and $y_{t}$:


$$\{\begin{array}{l} x_{t}=x_{t-1}+c_{t}+\epsilon_{t}^{x},\\ y_{t}=y_{t-1}+c_{t}+\epsilon_{t}^{y}, \end{array}$$ (12)

where $\epsilon_{t}^{x}$ and $\epsilon_{t}^{y}$ are the innovations at time *t* for $x_{t}$ and $y_{t}$, respectively, and $c_{t}$ represents a common drift (e.g., common inflationary pressures affecting both goods). Assume the innovations are independent and identically distributed (i.i.d.) random variables drawn from generalized error distributions (GED) with skewness and kurtosis:


$$\epsilon_{t}^{x}∼\text{GED}\left(\mu_{x},\sigma_{x},s_{x},k_{x}\right),\;\epsilon_{t}^{y}∼\text{GED}\left(\mu_{y},\sigma_{y},s_{y},k_{y}\right)$$ (13)

The difference between the two random walks is:


$$D_{t}=x_{t}-y_{t}=\left(x_{t-1}-y_{t-1}\right)+\left(\epsilon_{t}^{x}-\epsilon_{t}^{y}\right),$$ (14)

since the common drift term $c_{t}$ cancels out. This recursive relationship shows that $D_{t}$ itself is a random walk driven by the differences of the innovations:


$$D_{t}=D_{t-1}+\delta_{t},$$ (15)

with $\delta_{t}=\epsilon_{t}^{x}-\epsilon_{t}^{y}$. The probability density function (PDF) of $\delta_{t}$ is given by the convolution of the PDFs of $\epsilon_{t}^{x}$ and $\epsilon_{t}^{y}$:


$$f_{\delta }\left(\delta \right)=\overset{\infty }{\underset{-\infty }{\int }}f_{\epsilon^{x}}\left(\epsilon \right)\;f_{\epsilon^{y}}\left(\epsilon -\delta \right)\;d\epsilon$$ (16)

If $f_{\epsilon^{x}}$ and $f_{\epsilon^{y}}$ are non-normal GEDs with specific skewness and kurtosis parameters, their convolution $f_{\delta }\left(\delta \right)$ will also be non-normal and may exhibit increased skewness and kurtosis. This is because the difference of two independent non-normal random variables generally results in a non-normal distribution; normality would require specific conditions like exact cancellation of non-normal features, which is highly unlikely in practice. Consequently, when $x_{t}$ and $y_{t}$ are random walks driven by non-normal innovations, the difference accumulates these non-normal innovations over time. This challenges the assumption that $D_{t}$ could be normally distributed when $x_{t}$ and $y_{t}$ are different price processes with non-normal innovations because the distribution of $D_{t}$ will retain the skewness and kurtosis from the innovations.


$$D_{t}=x_{t}-y_{t}$$ (17)

In this argument, we only considered a common drift term as a source of correlation between $x_{t}$ and $y_{t}$. In practice, prices of two different but related goods may share more sources of similarity than a common drift term. However, we note that even when $x_{t}$ and $y_{t}$ share complex inter-dependencies that result in further correlation between the two series, such as a co-integrating relationship or dependence on lags in the first difference series $\Delta x_{t}$ and $\Delta y_{t}$, our main point that the presence of non-normal innovations in $x_{t}$ and $y_{t}$ affects the distribution of $D_{t}$ holds true, as can be shown by extending the arguments provided here. Further details are included in the supporting information section (S1 Text).

We distinguish three important levels of agreement based on normality of the linear difference equation, even in the presence of distributional differences:

1. **Normality in differences:** If the differences, D=X−Y, follow a normal distribution and the variances of *X* and *Y* are equal, this indicates a high level of agreement. Both datasets are likely tracking the same underlying price process without significant biases or systematic differences. Any deviations are attributed to normally distributed measurement errors.
2. **Normality in differences but unequal variance:** If the differences *D* are normally distributed but the variances of *X* and *Y* are not equal, the datasets are still likely tracking the same price process, but they exhibit different levels of variability. This suggests that one dataset is subject to higher measurement error variance. Unequal variances could result from differences in data collection methods or sample sizes. When *D* is not normal, unequal variance may also suggest differences in market conditions leading to higher short-run price volatility in one dataset. However, the normality of *D* implies that the variability is due to measurement error, meaning that one method is inherently more precise than the other.
3. **Normality in differences but unequal means:** If the differences *D* are normally distributed but the means of *X* and *Y* are not equal, the datasets are still likely tracking the same price process, but one source reflects a price markup. This suggests that one dataset is measuring prices at a different location or in a different quantity, where the same market conditions apply, but there is a fixed difference in price. It is likely, though not necessary, that the variances will also be unequal.

If none of these levels of agreement hold, and *D* is not normally distributed, it suggests that the datasets are capturing different price dynamics, and the deviations cannot be solely attributed to measurement errors. In such a case, a high correlation between the price series and the intention of both datasets to track the same price item provide circumstantial evidence that the datasets track similar goods. However, there is no conclusive statistical evidence to support that the underlying prices are indeed the same, despite providing a similar user experience.

To assess these levels of agreement, we conduct the following tests:

### Shapiro-wilk test for normality

We use the Shapiro-Wilk test to assess whether the differences *D* are drawn from a normal distribution. The hypotheses are:

- **Null hypothesis (**$H_{0}$): The differences *D* are normally distributed, implying that *X* and *Y* are consistent with the same underlying price process.
- **Alternative hypothesis (**$H_{a}$): The differences *D* are not normally distributed, suggesting that *X* and *Y* may be tracking different price processes or are subject to non-normal measurement errors.

The Shapiro-Wilk test is implemented by calculating the differences $D_{i}=X_{i}-Y_{i}$, ordering the sample values $D_{1},D_{2},\ldots D_{n}$ and comparing them against expected values under a normal distribution. If the test fails to reject $H_{0}$, it supports the conclusion that the AI-imputed and crowdsourced data are tracking the same underlying price process. However, if *x* and *y* are two different price processes, are unlikely to be normally distributed due to non-normality of the innovations propagating to the distribution of the difference sequence.

### Paired sample t-test for fixed price markup

We perform a t-test to assess whether the mean difference $E\left[D\right]$ is significantly different from zero or a fixed markup *τ*. The hypotheses are:

- **Null Hypothesis (**$H_{0}$): The mean difference $E\left[D\right]=\tau$, where *τ* represents a fixed markup.
- **Alternative Hypothesis (**$H_{a}$): The mean difference $E\left[D\right]\neq \tau$, implying that the two datasets differ by more than just a fixed markup.

The t-statistic is computed as:


$$t=\frac{\overset{ˉ}{D}-\tau }{s_{D}/\sqrt{n}}$$ (18)

where, $\overset{ˉ}{D}$ is the sample mean of the differences, $s_{D}$ is the standard deviation of the differences, and *n* is the number of paired observations. This test helps determine whether the datasets differ by more than a constant markup, providing further validation of their comparability.

### Variance Ratio Test (Equality of Variance)

We use the Variance Ratio Test (F-test) to determine whether the variances of *X* and *Y* are equal, which is important for the validity of the t-test. The hypotheses are:

- **Null Hypothesis (**$H_{0}$): The variances of the two datasets are equal, $\sigma_{X}^{2}=\sigma_{Y}^{2}$.
- **Alternative Hypothesis (**$H_{a}$): The variances of the two datasets are not equal, $\sigma_{X}^{2}\neq \sigma_{Y}^{2}$.

The test statistic is:


$$F=\frac{\sigma_{X}^{2}}{\sigma_{Y}^{2}}$$ (19)

## Results

### Main correlation analysis

The results of this study are presented in two layers, as summarized in Table 1. First, the table shows the correlations between the monthly-averaged crowdsourced and enumerator-submitted prices (in Naira per kilogram; ₦/kg) of yellow maize, white maize, Indian rice, and Thailand rice. Second, we present the relationship between the crowdsourced and monthly AI-imputed price data for these commodities.

**Table 1:** Summary of Commodity Price Relationships Across Data Sources.

Duration	Commodity	Paired Dataset	*r*	*r* ^*2*^	*p*
8 Months	Maize	Er (YM) – Cr (YM)	0.94	0.86	<0.001
		Er (WM) – *Cr* (WM)	0.94	0.86	<0.001
		Er (YM) – *Cr* (WM)	0.97	0.94	<0.001
		Er (WM) – *Cr* (YM)	0.90	0.77	0.003
		Er (MA) – *Cr* (MA)	0.92	0.84	<0.001
	Rice	Er (IR) – Cr (IR)	0.96	0.91	<0.001
		Er (TR) – Cr (TR)	0.78	0.55	0.022
		Er (IR) – Cr (TR)	0.20	0.04	0.63
		Er (TR) – Cr (IR)	-0.26	0.07	0.536
		Er (RA) – Cr (RA)	0.35	0.09	0.049
3 Years	Maize	Cr (WM) – AI (WM)	0.99	0.98	<0.001
		Cr (YM) – AI (WM)	0.99	0.97	<0.001
		Cr (MA) – AI (WM)	0.99	0.98	<0.001
	Rice	Cr (IR) – AI (ImpR)	0.93	0.86	<0.001
		Cr (TR) – AI (ImpR)	0.94	0.87	<0.001
		Cr (RA) – AI (ImpR)	0.94	0.88	<0.001

The table summarizes the relationships between commodity prices from three sources: enumerators (Er), crowdsourced data (Cr), and AI-imputed prices (AI). The analysis covers maize (M) and rice (R), with further classification into subtypes for Er and Cr data: yellow maize (YM), white maize (WM), Indian rice (IR), and Thailand rice (TR), while AI-imputed prices are available only for WM and imported rice (ImpR). MA represents all combined price data for both maize subtypes, and RA represents all combined price data for both rice subtypes. The Er-Cr price comparison is based on monthly values derived from intraday submissions over an eight-month period (March–October 2021), whereas the Cr-AI comparison spans three years (2019–2021) using monthly average Cr prices and AI-imputed closing prices. The number of individual data points from each source is as follows: during the eight-month period, Er (MA) =  1,381, Cr (MA) =  62,413, Er (RA) =  996, and Cr (RA) =  42,518; during the three-year period, Cr (MA) =  97,673, Cr (RA) =  69,063, AI (MA) =  144, and AI (RA) =  144. The correlation coefficient (*r*, ranging from -1 to + 1) indicates the direction of the relationship, and the coefficient of determination (*r²*, ranging from 0–1) indicates the strength of the relationship between paired price datasets. High *r* and *r²* values suggest reliability of the alternative price data. All tests performed at α =  0.05.

The analysis was conducted for both maize and rice prices, therefore, results will be discussed jointly. For clarity and focus, we mainly present charts for maize within this paper, while equivalent results for rice are included in text and as supporting information. The emphasis on maize is motivated by the universal availability of white maize prices across all three datasets. In contrast, the metadata for rice suggests potential minor differences in subtype across datasets. The analysis focuses on a monthly frequency, which adheres to current conventional cadence for national price assessment and reporting in Nigeria.

**Fig 2:**
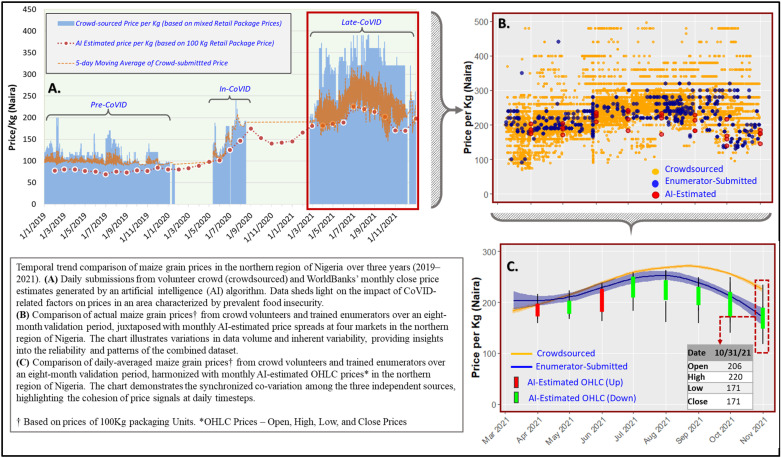
Temporal trends of white maize prices from AI-imputed and ground-truth sources (volunteer crowds and trained enumerators) in the northern region of Nigeria over three years (2019–2021). The top-left graph (A) illustrates the similarity in temporal trends between AI-imputed prices and the 5-day moving average of crowdsourced prices. The top-right graph (B) shows the volume of price data points from each source during the validation period (March 2021 – October 2021), with 112 data points for AI-imputed prices, 1,382 for enumerator data points, and 62,414 crowdsourced data points. The lower-right chart (C) shows the smoother trend of monthly prices across data sources during the validation period, with a 95% confidence interval band and a callout of the AI-imputed prices in the final month. The blue and yellow bands around the lines represent 95% confidence interval for each point along each trend line.

Overall, the prices of both commodities followed a similar trajectory in all datasets throughout the study period (as illustrated for maize in Fig 2), even during periods of market disruption. In the following paragraphs, we provide detailed analysis of how the correlations between the price datasets vary based on temporal alignment of datasets, aggregation over specific market segments, and aggregation by geographic boundaries. Table 1 shows that correlations between prices from paired data sources are higher when the commodity subtype is specified and identical between the datasets, especially for rice prices which are typically different between brands. The results from the formal equivalence tests, in subsequent sections, provides more statistical context for the price correspondence in relation to specificity of commodity subtype.

Significant correlations were observed between the price data for both rice and maize commodities submitted by trained enumerators and crowdsourcing volunteers during the eight-month period of concurrent ground-level data collection. The trend and progression of daily-averaged commodity prices from enumerators and crowdsourcing volunteers were comparable, despite some differences in daily price ranges and overall average prices, as shown for maize (Figs 2B and 2C). For instance, the overall average yellow maize price from enumerators and crowd volunteers averaged at ₦229.75/kg and ₦216.25/kg, respectively, but the difference was not statistically significant (*p = * 0.40). The variation observed in daily enumerator-submitted prices, ranging from ₦120/kg to ₦374/kg, was similar to daily average crowdsourced yellow maize prices, ranging from ₦156/kg to ₦296/kg. White maize prices exhibited similar trend and magnitude of difference as yellow maize for both data sources.

For rice, the price trends were consistent between both data sources, with some variation between sub-types. For instance, the average Indian rice price from enumerators (₦575/kg) was lower than average price from crowdsourcing volunteers (₦606/kg). In contrast, the average Thailand rice price reported by enumerators (₦649/kg) was higher than average price from crowdsourcing volunteers (₦625/kg).

**Fig 3:**
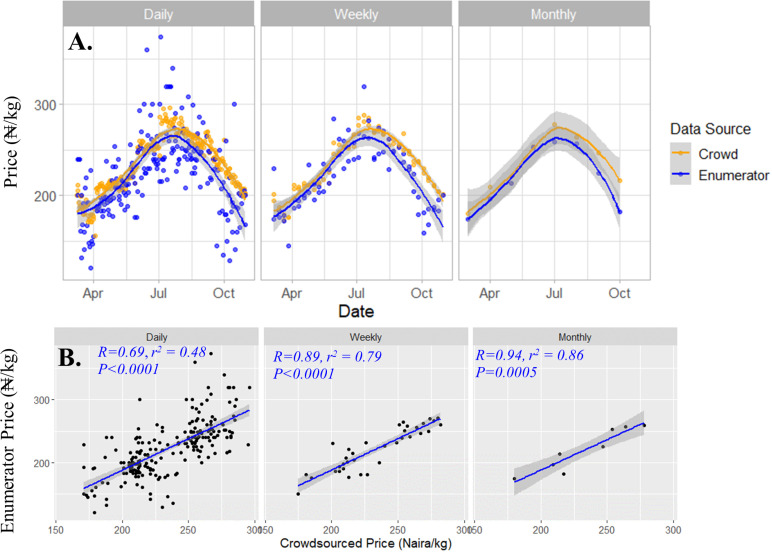
Co-evolution (A, upper) and relationship (B, lower) of white maize prices submitted by trained enumerators and volunteer crowd (crowdsourced) over an eight-month period (Mar 2021 – Oct 2021) in the northern region of Nigeria. The consistency of the price signals and their relationship improved as intraday data points were averaged into daily, weekly, and monthly time intervals. The correlation coefficient (*r*, ranging from 0-1) indicates the direction of the relationship, while the coefficient of determination (*r²*, ranging from 0-1) indicates the strength of the relationship between the paired price datasets. The straight lines represent best line of fit between the correlated price data pairs, while the grey band around the lines represent 95% confidence interval for each point along the respective regression or trend line. Higher *r* and *r²* values indicate better agreement between the datasets, suggesting the reliability of the crowdsourced prices relative to those collected by enumerators. Similar figure for rice is presented in the supporting information (S1 Fig).

Aggregating the intraday prices into daily, weekly, and monthly averages (e.g., Figs 3A and S1 Fig), revealed greater consistency between the two data sources for all commodities, and the correlation coefficient improved as the time interval increased. For yellow maize, the correlation changed from 0.69 at the daily interval to 0.94 at the monthly interval (Fig 3B). At the monthly interval, most of the variation in the crowdsourced data was corroborated by the variation in the enumerator-submitted prices, as indicated by the high *r²* values (*r*^*2*^ =  0.86 for maize and *r*^*2*^ =  0.91 for Indian rice). In contrast, the explained variation at weekly and daily intervals was lower across all commodities, likely due to the inherent noisiness of daily prices and the smaller sample sizes underlying the averages at these time steps.

Further analysis of the correlation between monthly averages of crowdsourced data and AI-based monthly estimates over a longer timeframe (36 months) revealed a strong relationship between both innovative data sources. Note that the prior assessment of crowdsourced and enumerator-submitted data indicated that the crowdsourced data was consistent with the ground-truth benchmark at this interval.

**Fig 4:**
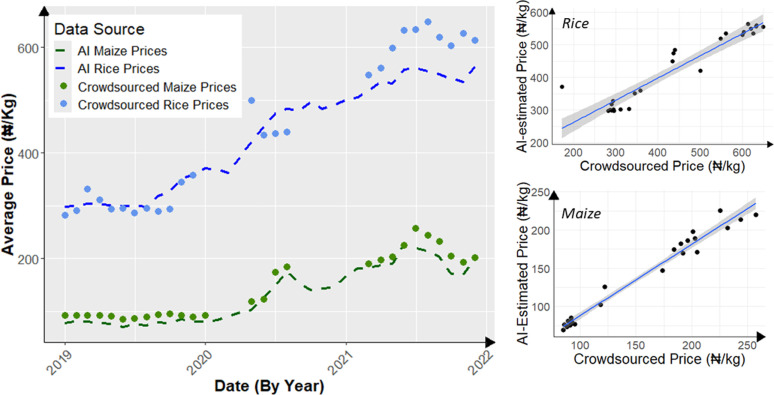
Temporal trend of average monthly maize and rice prices from volunteer crowd and AI- imputation in the northern region of Nigeria (A) and relationship between the monthly the monthly prices for maize (B) and rice (C) over a 3-year period (2021-2023). Crowdsourced prices represent monthly averages derived from intraday submissions by volunteers across over 150 geolocated market locations, while AI-imputed prices represent average estimated monthly close prices across four geolocated markets within the study region. The correlation coefficient (*r*, ranging from -1 to + 1) indicates the direction of the relationship, while the coefficient of determination (*r²*, ranging from 0-1) indicates the strength of the relationship between the paired price datasets. The straight lines represent best line of fit between the correlated price data pairs, while the grey band around the lines represent 95% confidence interval for each point along the respective regression or trend line. Higher *r* and *r²* values indicate better agreement between the datasets.

Broadly, between January 2019 and December 2021, the crowdsourced and AI-imputed price data showed comparable trends in prices of both commodities over time. For instance, the average monthly maize prices from both sources followed a similar trajectory, with a near-perfect correlation over the 36-month period (*r*² =  0.94; *p* < 0.001; Table 1 and Fig 4A and 4B). However, there was one exception in June 2021, when the AI-imputed maize price declined by 2.7% while the crowdsourced maize price increased by 9.3%, compared to the previous month. A similar divergence was observed for rice prices during this period (Fig 4B). Importantly, the alignment between the two data sources gradually resumed in the subsequent five months, as crowdsourced maize prices exhibited a steeper decline (-25.3%) compared to the AI-imputed maize prices (-12.4%). Despite these month-to-month variations in the crowdsourced data, the overall average monthly price of maize was slightly higher than the average AI-imputed closing prices (Table 1). In contrast, the average price of rice closely matched between crowd-submitted (₦435/kg) and AI-imputed (₦430/kg) data.

**Fig 5:**
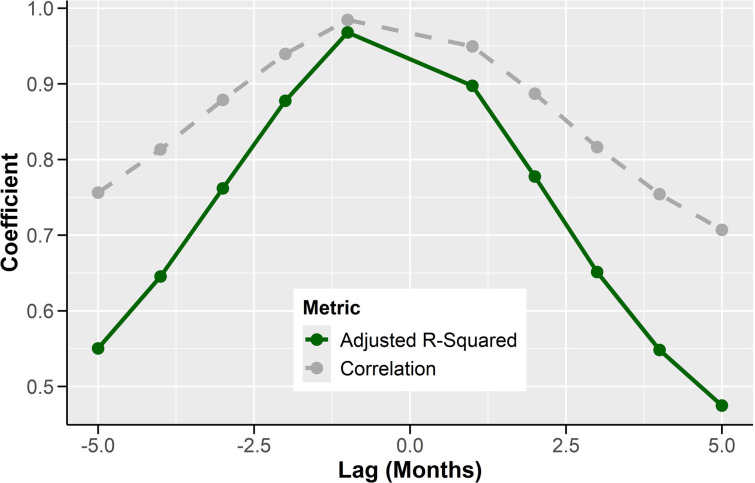
Time-shift relationship between monthly average prices of maize submitted by volunteer crowd (crowdsourced) and generated by artificial intelligence (AI) imputation. The AI-imputed prices were successively offset at monthly intervals, up to five months, to assess the temporal coherence of price signals from both data sources based on 3-year price datasets.

To assess the timeliness and temporal alignment of the datasets, time-shifted correlation analyses were conducted. The results showed that crowdsourced prices were most strongly correlated with AI-imputed prices for the current month, with an *r²* value of 0.97 for maize. This correlation diminished when AI prices were compared to crowdsourced prices from one month later (*r²* =  0.90 for maize) and decreased further when increasing the time shift. The correlation between the datasets weakened almost linearly and the *r²* dropped to 0.55 and 0.48 by the fifth month of lag and lead respectively (Fig 5). This indicates that the AI-imputed close prices accurately reflect current market prices and are a reliable source for real-time price monitoring.

**Fig 6:**
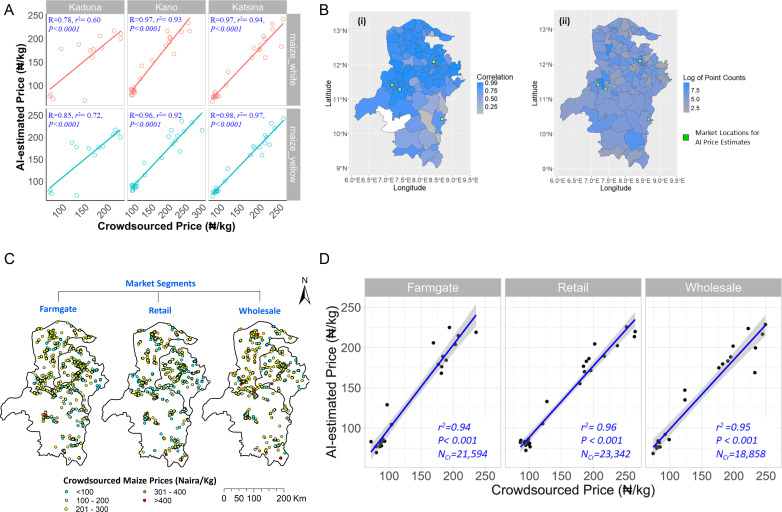
A. Relationship between crowdsourced and AI-imputed maize prices, disaggregated by state and commodity subtype within the northern region of Nigeria. Crowdsourced prices, submitted daily by volunteers over a 3-year period (2019–2021), are averaged into monthly values. High correlations were observed across all states and by commodity subtypes. The correlation coefficient (*r* ranging from -1 to + 1) and the coefficient of determination *r²* (ranging from 0-1) quantify the reliability of AI-estimated prices. The straight lines represent best linear line of fit between the correlated price data pairs. The *p*-value indicates the significance of the relationship at α =  0.05. A similar figure is available for rice in the supporting information (S2 Fig). B. Map of study area, with Admin 2 boundaries, in northern Nigeria, showing (i) local correlation between crowdsourced and AI-imputed maize prices at the nearest market, and (ii) log-transformed count of submissions in the crowdsourced dataset, indicating the sample size underpinning the average maize prices in the sub-national correlation analysis. The value for counts were log-transformed to normalize the skewed distribution and improve visual representation of the map. Both maps show that the target market locations for AI-imputation are within Admin 2 boundaries where there is moderate to high count of submissions and where high correlations were achieved. The correlation coefficient (*r*, ranging from -1 to + 1) was calculated based on monthly average prices from both data sources over a 36-month period (January 2019–December 2021). The administrative boundary outlines are mapped using shapefile data from GADM, available under a free license (www.gadm.org). C. Spatial distribution of crowdsourced maize prices across three major market segments within the northern region of Nigeria. Each point represents a georeferenced price quote submitted by volunteers through a smartphone-based app. The administrative boundary outlines are mapped using shapefile data from GADM, available under a free license (www.gadm.org). D. Relationship between crowdsourced and AI-imputed maize prices across three major market segments within the northern region of Nigeria. Crowdsourced prices were collected from volunteer submissions over a 3-year period (2019–2021) and averaged into monthly values. The AI-imputed monthly close prices were averaged over four market locations within the study region. *N*_*Cr*_ denotes the number of crowdsourced data points included in the analysis during the period. The coefficient of determination (*r²*; ranging from 0-1) was tested for significance at α =  0.05. The straight lines represent best line of fit between the correlated price data pairs, while the grey area around the line represent 95% confidence interval for each point on the regression line. The figure shows strong covariance between both price datasets in each of the market segments.

The strong relationship observed between crowdsourced and AI-imputed maize and rice prices remained consistent when the datasets were disaggregated by geographic region (Admin1 level, or “States”) and market segments. This disaggregation involved querying the crowdsourced dataset for price data points associated with each state or market segment. For example, average monthly crowdsourced maize prices were comparable to AI-imputed prices by location, with values of ₦154/kg vs. ₦156/kg in Kaduna, ₦152/kg vs. ₦139/kg in Kano, and ₦148/kg vs. ₦134/kg in Katsina. The correlations between crowdsourced and AI-imputed maize and rice prices were consistently strong within each state (*r* ranging from 0.72 to 0.98, *p* <  0.001, Figs 6A and S2 Fig). At the Admin2 level, the correlation of maize prices was slightly stronger when crowdsourced data was compared to the nearest AI-targeted market location (*r* ranging from 0.81 to 0.99; Fig 6B). This highlights that the AI-imputed prices captured spatial variation reliably at the local level.

When maize price datasets were disaggregated and compared by market segments, the correlation remained consistently strong across farmgate, retail, and wholesale segments, with *r²* values ranging from 0.94 to 0.96 (Figs 6C and 6D). This indicates that the AI-imputed prices are reliable for drawing inferences about price changes across different market segments. Crowdsourced maize prices showed the highest concordance with AI-imputed maize prices at the retail market segment (*r*^*2*^ = 0.96), with slightly lesser agreement at the farmgate market segment (*r*^*2*^ = 0.94). Similarly, average crowdsourced price of rice showed stronger correlation with AI-imputed price at the retail market segment (*r*^*2*^ =  0.82), compared to farmgate market segment (*r*^*2*^ = 0.55). The lower correlation suggests there may be higher variation in prices of Indian rice and Thailand rice, which are both imported as opposed to maize that is locally produced, across farmgate locations.

### Formal equivalence testing of specific food commodities

The strong correlations suggest alignment between the alternative data sources. However, formal statistical tests (Table 2) provided deeper insights into whether these sources track the same commodities or merely align for inflation trends.

**Table 2:** Results of formal equivalence tests to assess correspondence between weekly enumerators (Er) and crowdsourced data (Cr) commodity prices over an eight-month period (March - October 2021), and between monthly crowdsourced data (Cr) and AI-imputed (AI) food prices, over a 3-year period (2019-2021).

Temporal Frequency	Paired Price Data		SW-test		t-test		VR-test	
		*n*	*W*_*stat*_	*p*	*t*_*stat*_	*p*	*F*_*stat*_	*p*
Weekly	*ln*Er (YM) – *ln*Cr (WM)	35	0.96	0.23	-4.50	0	1.42	0.31
	*ln*Er (WM) – *lnCr* (WM)	35	0.97	0.44	-5.59	0	1.08	0.83
	*ln*Er (IR) – *ln*Cr (IR)	35	0.97	0.35	-5.06	0.42	6.53	0
	*ln*Er (TR) – *ln*Cr (TR)	35	0.97	0.32	3.82	0.54	1.68	0.14
Monthly	*ln*Cr (YM) – *ln*AI (WM)	27	0.91	0.03	-10.69	0	0.96	0.89
	*ln*Cr (WM) – *ln*AI (WM)	27	0.96	0.34	-8.41	0	0.97	0.93
	*ln*Cr (IR) – *ln*AI (ImpR)	26	0.77	0	0.82	0.42	0.37	0.01
	*ln*Cr (TR) – *ln*AI (ImpR)	27	0.66	0	0.61	0.54	0.47	0.04

SW-test is Shapiro-Wilk test of normality, t-test is paired t-test of means, VR-test denotes variance ratio test, YM is yellow maize, WM is white maize, IR is Indian rice, TR is Thailand rice, ImpR is Imported Rice; *ln* denotes log-transformation, *n* is the number of datapoints, while *W*_*stat*_, *t*_*stat,*_ and *F*_*stat*_ are computed statistics for each respective tests, and *p* denotes the probability of the test statistics exceeding the critical values. The Shapiro-Wilk test establishes equivalence up to residual measurement error if the individual series’ innovations follow a non-normal distribution and the *p*-value for the linear difference sequence does not reject normality (*p* > 0.1). Supporting results (S1 Table) show that first-order differences of log-transformed crowdsourced and AI prices were non-normal for both maize and rice, as expected for commodity prices. Due to limited data points (n = 8), this test was not applied to monthly averages of enumerator and crowdsourced prices. However, equivalence established at the weekly level suggests that non-normality observed in crowdsourced data likely extends to enumerator data.

At the weekly level, Shapiro-Wilk tests indicated equivalence between crowdsourced and enumerator data for all paired commodities (*p* ranging from 0.23 to 0.44 for yellow maize, white maize, Indian rice, and Thai rice) during the eight-month period (March–October 2021). The equivalence tests confirmed that enumerator and crowdsourced prices reflect the same price processes for specific subtypes, supporting the reliability of crowdsourced data as a ground truth for benchmarking AI-imputed prices. Over the 36-month period, crowdsourced prices aligned fully with AI-imputed prices for white maize (p = 0.34) but not for rice (p < 0.001). As expected, correspondence was stronger when metadata indicated an identical commodity subtype (white maize), and no formal equivalence was established between the price series when the metadata lacked specificity (e.g., imported rice in the AI-imputed data, which could represent either Thailand or Indian rice, or a mix, with ratios varying over time).

To confirm the sensitivity of our tests to subtype differences, the Shapiro-Wilk test was also performed on crowdsourced yellow maize and AI-imputed white maize prices. The test confirmed equivalence when both datasets represented white maize but rejected equivalence when switching the crowdsourced data pair to yellow maize (p = 0.03), indicating sensitivity to subtype differences and supporting the notion that rice price discrepancies likely reflect differing price dynamics attributable to subtype differences. Similarly, variance ratio tests revealed differences in variance between enumerator and crowdsourced prices for maize and rice (Table 2). The larger variance in enumerator data (positive F-statistics) likely reflects greater variability in single monthly price datapoints compared to crowdsourced data, which averages multiple submissions over the month. This difference was significant only for Indian rice. For maize, no significant variance differences were found between crowdsourced and AI-imputed prices. For rice, crowdsourced data showed greater variance than AI-imputed data, but the lack of equivalence for rice subtypes suggests differing price dynamics. Earlier results (Table 1) support this conclusion, because no meaningful relationship emerged when the crowdsourced datasets for Thailand rice and Indian rice were combined as a single dataset (i.e., RA), prior to correlation with AI-imputed prices for imported rice.

The *t*-tests revealed significant differences in price levels across the three data sources. For white maize, AI-imputed prices consistently showed a markdown (t =  − 8.41, *p* < 0.001), likely due to wholesale packaging discounts (100 kg bags) and reduced transport costs at central AI-targeted markets. For rice, no markdown was observed. AI-imputed prices for 50 kg bags aligned closely with crowdsourced and enumerator prices, potentially indicating either no volume discount for 50 kg bags or that enumerator and crowdsourced data tracked lower-quality rice, while AI-imputed data reflected higher-quality grains with a volume discount.

## Discussion

This correlative analysis of crowdsourced and AI-imputed price datasets provides valuable insights into the inherent credibility of data generated through both approaches, compared to enumerator-submitted ground-truth prices. While new data innovations often promise increased data volume, variety, velocity, and high-throughput processing, they also raise important questions about their trustworthiness and accuracy, especially under rapidly changing or high-entropy conditions, such as volatile markets and fragile contexts. It is essential to ensure that these new data approaches meet the minimum standards for validity and reliability, in comparison to traditional methods.

The innovative crowdsourcing of food commodity prices and the deployment of AI algorithms for price imputation are promising strategies for frequent monitoring of food prices and near-real-time assessment of market signals to support food security analysis and economic monitoring. Both strategies are relatively new and evolving independently, showing promising results so far. This paper addresses two fundamental questions relevant to stakeholders, including the data science and policy communities, and provides evidence regarding the accuracy of these datasets. This evidence can guide the application of these alternative data approaches for other data-centric use cases. The first question examines whether there is a credible relationship between conventional enumerator-submitted price data and crowdsourced price data within the same geographical context. The second question explores whether there is a meaningful relationship between crowdsourced commodity prices and AI-imputed prices. Although these questions may seem straightforward, they are far from trivial, as answering them requires independence in each data collection method and alignment across at least six key elements: national context, timeframe, geographic locations, market segments, commodity types, and packaging units. The opportunity to explore the relationships between these datasets depended on the overlap of the focal geography (Fig 1), co-occurrence of selected commodities, and the intersection of the temporal data granularity (i.e., intraday, daily, or monthly).

The answers to these questions ultimately carry important relevance to determine whether these innovations present viable alternatives to gold standard enumerator-collected data and are reliable enough to track price dynamics in challenging situations where traditional data collection methods are not viable. The value of both AI-imputation and crowdsourcing goes beyond merely understanding current or past commodity price trends. These innovative approaches have already enabled significant progress toward forecasting emerging food crises, using the rich data flow to detect vulnerability of households to shocks and stressors [29,30,40].

### AI-imputation, crowdsourcing and enumerator data as reliable complements

Our findings demonstrate that both AI-driven imputation and crowdsourcing can reliably track commodity prices, offering new ways to gather timely, localized data on food markets. Despite the challenging context of northern Nigeria, marked by conflict, food insecurity, and limited data availability, we found that crowdsourced and enumerator-collected prices closely matched over an eight-month validation period, with strong correlation coefficients (*r²* =  0.86 for maize and *r²* =  0.91 for rice). Over a longer timeframe, crowdsourced data also aligned closely with AI-imputed prices, showing near-perfect agreement for maize (*r²* =  0.98) and high agreement for rice (*r²* =  0.88).

Aggregating prices from various packaging units, market types, and locations could potentially affect the strength of correlation estimates. However, we found no evidence that this aggregation compromised the relationship between crowdsourced and AI-imputed prices. Generally, the AI-imputed prices are based on wholesale units (50 kg and 100 kg packages) and represent wholesale prices [8]. Meanwhile, the crowdsourced data include a variety of selling packages across farmgate, retail, and wholesale segments, with metadata distinguishing these units [22,41]. Consistent with previous studies [42], the price per unit was lower in the wholesale segment compared to the retail segment. Therefore, the strong correlation between AI-imputed prices and the wholesale subset of the crowdsourced price for maize was not surprising (*r²* = 0.95). Notably, the AI-generated data also accurately reflected market signals across farmgate and retail prices (Fig 6D), which aligns with the dynamics of markets in LMICs, where commodities are transacted in different packaging units within and between market locations. Effective price monitoring systems must be robust enough to track prices across various market segments and packaging units. Our analysis shows that both crowdsourced and AI-imputed price datasets are reliable for generating insights and metrics across market segments.

Formal statistical tests suggest that observed discrepancies between datasets can be attributed to measurement error rather than genuine differences in underlying price dynamics. Equivalence tests confirmed that enumerator and crowdsourced prices represent the same price processes for specific commodity subtypes, establishing crowdsourced data as a credible reference for validating AI-imputed estimates. When the metadata from all sources described identical commodities, such as white maize, the datasets tracked the same market price signals. However, when commodity classifications were generic, as with “imported rice,” the alignment was weaker, emphasizing the importance of product-specific metadata for accurate comparisons.

In terms of precision, crowdsourced data exhibited lower variance than enumerator-collected data at the weekly level, while AI-imputed data showed the lowest variance at the monthly level, although this difference was not statistically significant. These findings suggest that crowdsourced and AI-imputed data may sometimes offer equal or even superior precision compared to traditional enumerator-based methods, long considered the benchmark in many monitoring systems.

Nonetheless, the t-tests revealed differences in price levels across data sources, indicating potential challenges posed by the dynamic and geographically diverse nature of crowdsourced inputs. Crowdsourcing’s flexibility can yield rich, granular insights into local market conditions but may also introduce variability that must be carefully managed and interpreted.

Taken together, these results support using both AI imputation and crowdsourcing approaches to enhance and complement traditional price data collection. In contexts where food prices are subject to rapid shifts, especially during crises, high-frequency local data can offer critical, near real-time intelligence. Such methods are particularly beneficial in subnational regions often overlooked in national datasets, where targeted interventions depend on reliable, fine-grained, and timely information. As innovations in data collection and modeling continue to evolve, incorporating crowdsourced and AI-imputed datasets can strengthen early warning systems, improve the responsiveness of humanitarian efforts, and ultimately contribute to more resilient and equitable food systems.

### Challenging perceptions: Crowdsourced price data and AI-imputed data as viable alternatives to the gold standard

As efforts to apply crowdsourcing for high-frequency, large-volume data collection gain momentum [24,43], a common perception persists that citizen volunteers may not be able to submit data as credible as that from trained enumerators [17]. However, the strong relationship between conventional enumerator-submitted prices and crowdsourced prices challenges this notion, showing that high-frequency price datasets from citizens or market actors can be both useful and valid. This evidence can positively influence perceptions about the dependability of volunteers to consistently submit accurate price data and may encourage stakeholders to adopt crowdsourcing for data collection and market price assessment.

It is noteworthy that the underlying price trends in crowdsourced data became more apparent when intraday data points were averaged into daily, weekly, and monthly values. This effect is due to averaged price values converging around modal values, which reduces the influence of outliers and enhances the signal-to-noise ratio. It also justifies our focus on comparing crowdsourced prices with AI-imputed prices at monthly intervals. The emergence of discernible price signals from the volume of crowdsourced data points can be valuable for various applications in food system analysis and decision-making. Detecting price signals enables the tracking of seasonal price dynamics and supports the development of agile analytical systems that link commodity prices to socio-economic and ecological factors. Furthermore, the temporal granularity of price monitoring systems should align with the time scale of market-focused policies or food system interventions. High-frequency data points can be aggregated as needed, offering the flexibility to generate insights and support decision-making in fragile national or regional contexts.

In contrast, the presence of outliers in enumerator-submitted prices raises potential concerns about this method, despite the understanding that it is often considered as the gold standard. Enumerator-collected data represent single-source measurements vulnerable to individual errors. In contrast, crowdsourcing inherently provides large-sample smoothing, while imputation methods incorporate low-pass filtering, offering greater resistance to outliers. As shown in Fig 3A, outliers in the daily enumerator price data are sharply noticeable due to the limited number of enumerators [16] compared to the much larger number of volunteer submissions, which tallied up to hundreds of daily datapoints. The larger volume of crowdsourced data points allowed daily averaging to smooth out potential outliers, suggesting that crowdsourced data better represent the prevailing market prices across the study area, benefiting from a more robust sample size and broader spatial coverage.

The strong correlation between AI-imputed prices and crowdsourced data over an extended period (36 months) highlights the potential of both methods to support timely assessments of market price changes. However, even though enumerator-led data is sensitive to outliers, the small sample nature also allows tracing errors more easily to source. Both Crowdsourcing and AI efforts are big data in nature, and when errors occur, they are less easily detected. Both systems require statistical quality checks to detect and correct outliers on the fly, data system maintenance, and extensive ongoing validation. Ultimately, neither system can be fully fault-proof and these systems cannot operate without a ground truth. Although the AI-imputed monthly price estimates are regularly cross-validated [8], this additional validation against independent, ground-level data from volunteers provides a new perspective on the utility of AI-generated price data. The near-perfect agreement between the two sources furthermore suggests an opportunity to integrate ground-level price monitoring with AI-based systems, creating a hybrid high-frequency price monitoring system.

A future AI-driven price data system could be further enhanced with an integrated validation component that frequently incorporates ground-truth data, including from crowdsourced inputs, to maintain and demonstrate reliability. Conceptually, crowdsourced data could provide georeferenced intraday price points, offering a spatially rich stream of training and testing data for AI algorithms, thus improving model performance and data output credibility. Conversely, AI algorithms can generate price data and insights at multiple administrative levels, especially in areas where crowdsourcing is less effective or results in sparse data.

### Potential limitations and caveats for crowdsourced and AI-imputed prices

While this study suggests the overall reliability of the alternative data sources, some limitations should be considered when interpreting the findings.

First, because the FPCA was implemented in two discontinuous phases, there were two short gaps (4 and 6 months) in the temporal trend of the crowdsourced data within the 3-year period (Fig 4A). This highlights both the challenges of producing continuous data through traditional collection methods and the advantages of automated price estimation systems. Despite the gap in the FPCA data during a sharp level shift in prices, the correlation with AI-imputed prices remained strong, reflecting that both datasets accurately captured major price shifts over time. The data period included the disruptive shock of the COVID-19 pandemic, which caused unanticipated price swings. During this period, crowdsourced data provided real-time insights that were otherwise unavailable [16], while the sensitivity of AI-imputed prices to such disruptions had not been fully proven. The strong correlation between crowdsourced and AI-imputed prices, even during transitional phases, suggests that the underlying algorithms effectively tracked overall price shifts and monthly price movements.

Second, real-time AI-based price estimates are currently available for 36 countries, with comparable survey data from various organizations potentially allowing this approach to scale to other high-priority countries. However, AI algorithms can be constrained by a lack of ground truth data to validate model outputs. Given the limited availability of food price data for model calibration in LMIC contexts [8], it is reasonable to assume that models might exhibit instability over time. This could result in incorrect price estimates, especially over longer periods. However, no evidence of such model instability was found in the data analyzed, rather, we found evidence that specificity of commodity subtype can enhance the accuracy of imputed prices relative to ground observations. It is relevant to note that price signals from the previous month(s) may significantly influence subsequent price estimates, and point towards a need to assess whether correlations of alternative dataset with ground-truth prices exhibit time lags. The time-lag analysis of paired datasets was limited to monthly intervals (up to 5 months forward and backward) and this range was sufficient to show that both data sources displayed real-time coherence in monthly price trends, supporting the notion that AI-imputed prices can provide timely insights. As AI-imputation methods are further developed to produce price estimates at finer time scales (e.g., daily or weekly), future studies should revisit the issue of timeliness by analyzing degrees of correspondence across time lags.

## Conclusion

This assessment provides strong evidence supporting the validity and complementarity of crowdsourced and AI-imputed prices within a specific geographic context. First, the high positive correlation and equivalence between enumerator-submitted and crowdsourced prices establishes a basis for accepting crowdsourcing as a reliable means of generating ground-truth equivalent data with broad spatial coverage and temporal depth. Second, by demonstrating that AI-imputed data aligns well with the crowdsourced price observations, this study validates the use of AI-imputed prices for real-time assessment of price trends. This conclusion is further supported by the finding that there is no time lag associated with the highest correlation of crowdsourced and AI-imputed prices (Fig 5). Additionally, the high correlation between crowdsourced and AI-imputed prices at finer geographic levels (as shown in Fig 6B) underscores the reliability of these alternative data sources for use in local contexts.

As both innovative approaches continue to evolve, the findings from this study can guide the refinement and application of these methods toward addressing data gaps in food price monitoring systems in LMICs. This can contribute to the advancement of data-rich analytics, including now-casting and forecasting of food affordability and security over time, at regional, national, and sub-national levels [44].

Invariably, the deployment of these methods should be guided by ethical considerations, including privacy and do-no-harm principles. Moreover, issues related to system set-up strategies to effectively engage crowdsourcing volunteers, the incentive structure for data submission, and controls for data quality and privacy remain critical for sustainable price crowdsourcing. Future directions should include expanding the coverage to broader markets within each sub-national geography, incorporating a wider range of commodities and sub-types, and replicating similar validation workflows in other countries to diversify the contexts in which these methods are applied. Outcomes may vary in other geographical contexts where opportunity to crowdsource data may be limited by capacity and access for phone-based data submission, while AI-based price imputation can be constrained by availability of independent input data to train models.

## Supporting information

S1 Text Equivalence theory for dynamically related price series. These additional texts and equations show the theoretical models guiding the assessment of price relationship over time.
(PDF)

S1 Fig Co-evolution (A) and relationship (B) of Thailand rice prices submitted by trained local enumerators and volunteer crowds (crowdsourced) over an eight-month period (March 2021 – October 2021) within the northern region of Nigeria. The correlation between the price datasets improved as the numerous intraday data points were averaged into daily, weekly, and monthly time intervals. The straight lines represent best line of fit between the correlated price data pairs, while the grey area around the lines represent 95% confidence interval for each point along the respective regression or trend line.
(TIF)

S2 Fig Relationship between crowdsourced and AI-imputed prices of imported rice in the northern region of Nigeria, disaggregated by state (Admin1) and commodity subtype (Indian rice and Thailand rice). The intraday volunteer-submitted crowdsourced prices were collected over a 3-year period (2019–2021) and averaged into monthly values. The AI-imputed monthly closing prices were averaged over four market locations within the study region. *r²* denotes the coefficient of determination, and the significance of the relationship is tested at α =  0.05. The straight lines represent best line of fit between the correlated price data pairs.
(TIF)

S1 Table Results of normality tests on first order differential of commodity prices from enumerators (Er), crowdsourcing (Cr), and artificial intelligence imputation (AI). *δ
* denotes first order price differential, *ln* denotes log-transformation of prices (₦/kg), *n* is the number of datapoints, YM is yellow maize, WM is white maize, IR is Indian rice, TR is Thailand rice, ImpR is Imported Rice; *W*_*stat*_ *i*s the computed statistics for the normality test, and *p* denotes the probability of the test statistics exceeding the critical values, based on 95% confidence limit at α= 0.05. The AI-imputed datasets are represented at monthly timesteps, while the Er and Cr datasets are represented at weekly timesteps.
(DOCX)
