# Supplementary material for: AI-imputed and crowdsourced price data show strong agreement with traditional price surveys in data-scarce environments
Source: PLoS One. 2025 Apr 8;20(4):e0320720. doi: 10.1371/journal.pone.0320720 (PMC11978078; doi:10.1371/journal.pone.0320720)
Supplement: S1 Text — These additional texts and equations show the theoretical models guiding the assessment of price relationship over time. (PDF) [file pone.0320720.s001.pdf]

Note that, in a Vector Autoregression, VAR model:

$$\begin{cases} \Delta x_t = \phi_{11}\Delta x_{t-1} + \phi_{12}\Delta y_{t-1} + \epsilon_t^x, \\ \Delta y_t = \phi_{21}\Delta x_{t-1} + \phi_{22}\Delta y_{t-1} + \epsilon_t^y, \end{cases} \tag{1}$$

or in a Vector Error Correction Model, VECM:

$$\begin{cases} \Delta x_t = \alpha_1(y_{t-1} - \lambda x_{t-1}) + \epsilon_t^x, \\ \Delta y_t = \alpha_2(y_{t-1} - \lambda x_{t-1}) + \epsilon_t^y, \end{cases} \tag{2}$$

the difference $D_t = x_t - y_t$ still depends on lagged differences but this time the innovation δ_t incorporates both the differences of the innovations and the effects of the model dynamics. Specifically, for the VAR model, δ_t may be expressed as:

$$\delta_t = (\phi_{11} - \phi_{21})\Delta x_{t-1} + (\phi_{12} - \phi_{22})\Delta y_{t-1} + \left(\epsilon_t^x - \epsilon_t^y\right), \tag{3}$$

while for the VECM model it is:

$$\delta_t = (\alpha_1 - \alpha_2)(y_{t-1} - \lambda x_{t-1}) + \left(\epsilon_t^x - \epsilon_t^y\right). \tag{4}$$

In both cases, δ_t captures the combined effects of the model dynamics and the innovation differences. Even though VAR and VECM dynamics introduce dependencies on lagged values and error correction mechanisms, they still result in specific forms for δ_t that include the non-normality of the innovations. Therefore, they do not change the fundamental argument regarding the distribution of D_t. Specifically, the probability density function (PDF) of δ_t is determined by the convolution of the PDFs of the individual innovations and any additional terms from the model dynamics:

$$f_\delta(\delta) = \int_{-\infty}^{\infty} f_{\text{model}}(\theta) \int_{-\infty}^{\infty} f_{\epsilon^x}(\epsilon)\, f_{\epsilon^y}\big(\epsilon - (\delta - \theta)\big)\, d\epsilon\, d\theta, \tag{5}$$

where $f_{\text{model}}(\theta)$ represents the distribution of the terms arising from the temporal dynamics. Although considering temporal dynamics makes the convolution more complex, the key point remains: if the innovations are non-normal, δ_t will also be non-normal, and this non-normality accumulates in D_t. The normality assumption is however realistic for the measurement errors e and v, which are expected to be random and normally distributed due to the Central Limit Theorem, assuming they arise from numerous small, independent sources of error. We can exploit this and test the normality of D to determine whether the differences between X and Y are primarily due to normally distributed measurement errors or a reflection of differences in the underlying price processes x and y.
