# Supplementary figures and images for: AI-imputed and crowdsourced price data show strong agreement with traditional price surveys in data-scarce environments
Source: PLoS One. 2025 Apr 8;20(4):e0320720. doi: 10.1371/journal.pone.0320720 (PMC11978078; doi:10.1371/journal.pone.0320720)

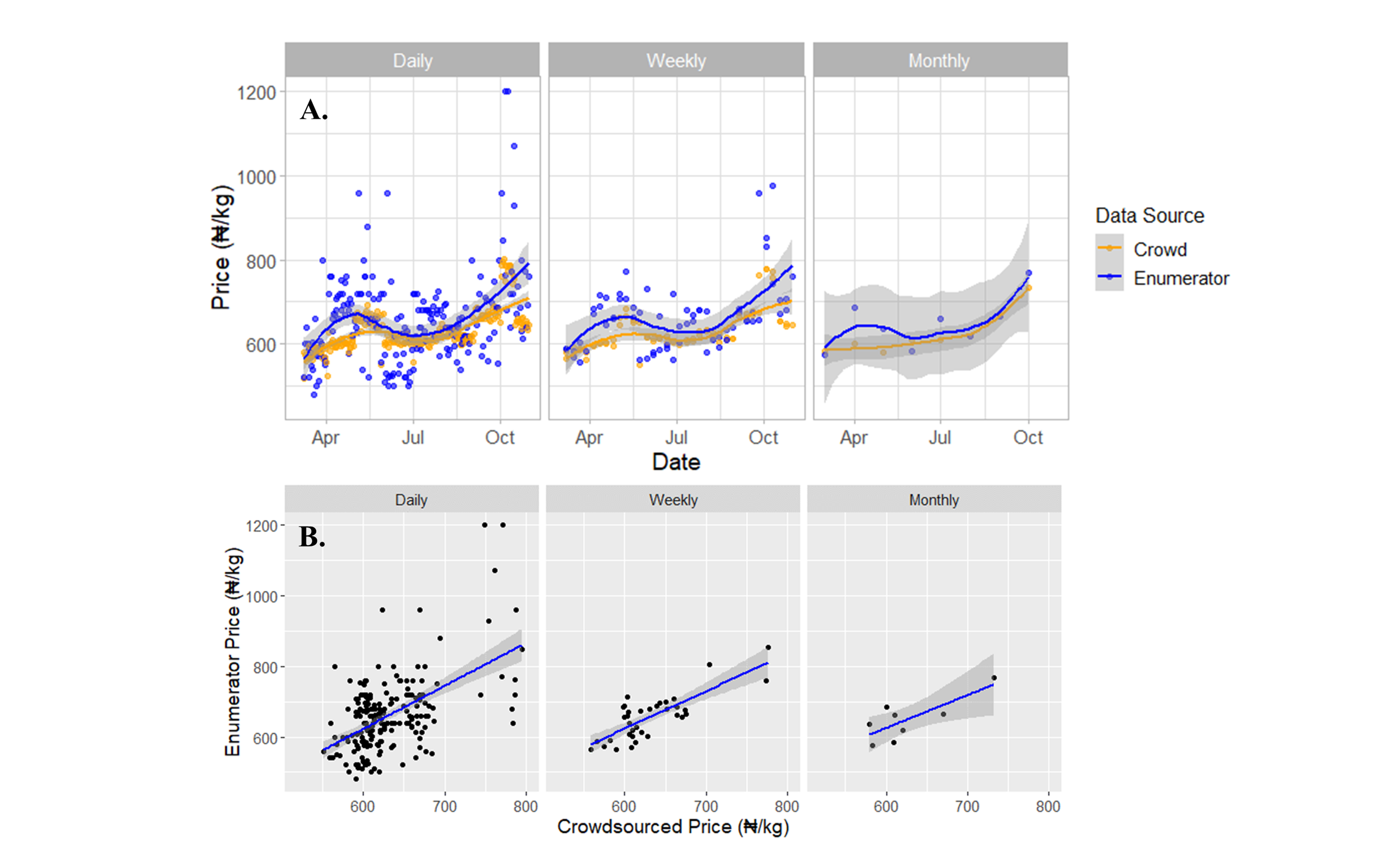

Supplement: S1 Fig — The correlation between the price datasets improved as the numerous intraday data points were averaged into daily, weekly, and monthly time intervals. The straight lines represent best line of fit between the correlated price data pairs, while the grey area around the lines represent 95% confidence interval for each point along the respective regression or trend line. (TIF) [file pone.0320720.s002.tif]

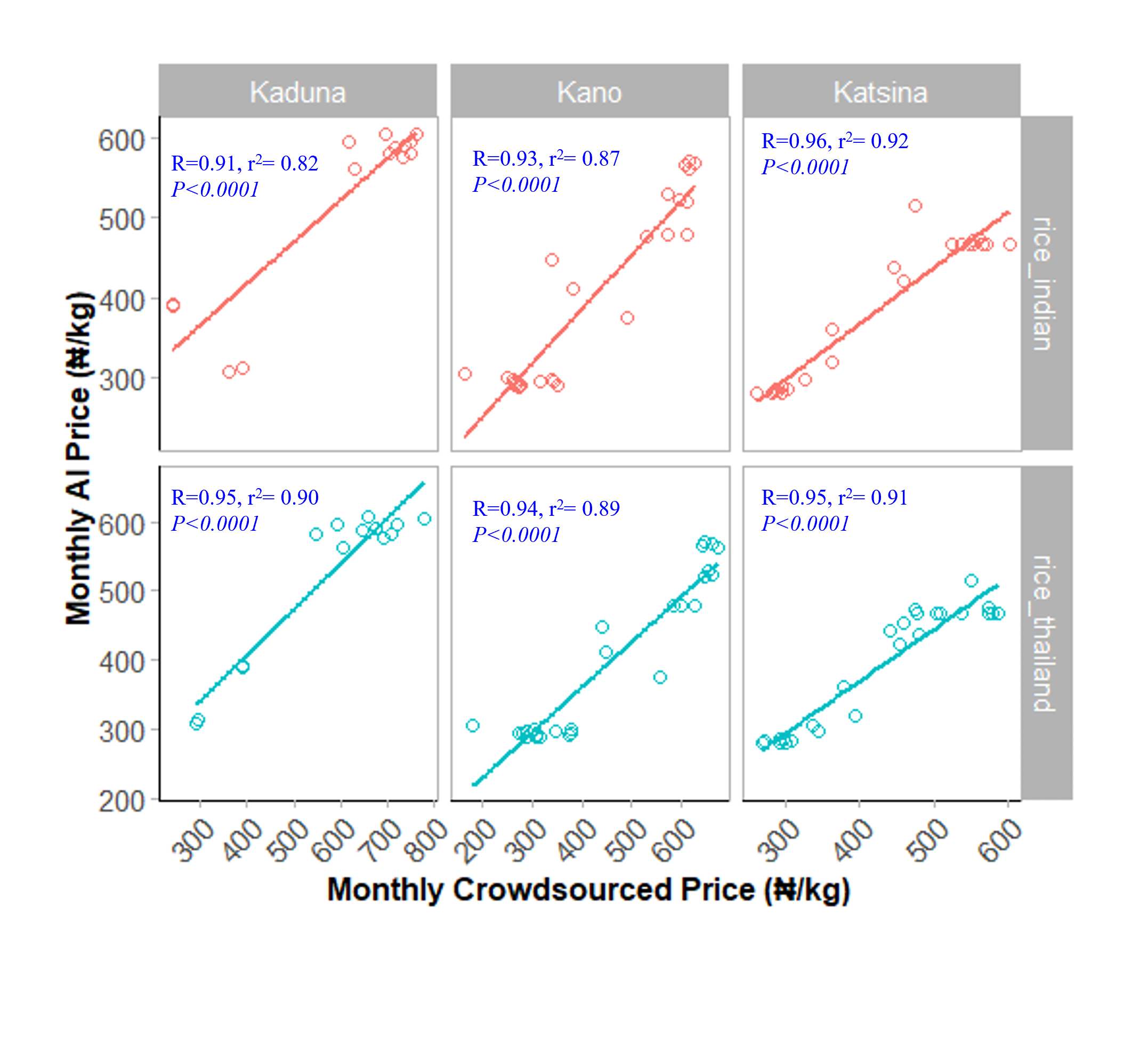

Supplement: S2 Fig — The intraday volunteer-submitted crowdsourced prices were collected over a 3-year period (2019–2021) and averaged into monthly values. The AI-imputed monthly closing prices were averaged over four market locations within the study region. r² denotes the coefficient of determination, and the significance of the relationship is tested at α = 0.05. The straight lines represent best line of fit between the correlated price data pairs. (TIF) [file pone.0320720.s003.tif]
