# Supplementary material for: AI-imputed and crowdsourced price data show strong agreement with traditional price surveys in data-scarce environments
Source: PLoS One. 2025 Apr 8;20(4):e0320720. doi: 10.1371/journal.pone.0320720 (PMC11978078; doi:10.1371/journal.pone.0320720)
Supplement: S1 Table — denotes first order price differential, ln denotes log-transformation of prices (₦/kg), n is the number of datapoints, YM is yellow maize, WM is white maize, IR is Indian rice, TR is Thailand rice, ImpR is Imported Rice; Wstat is the computed statistics for the normality test, and p denotes the probability of the test statistics exceeding the critical values, based on 95% confidence limit at α= 0.05. The AI-imputed datasets are represented at monthly timesteps, while the Er and Cr datasets are represented at weekly timesteps. (DOCX) [file pone.0320720.s004.docx]

| **Commodity** | **Price**  **Data** |  | **Shapiro-Wilk Test** | |
| --- | --- | --- | --- | --- |
|  |  | *n* | *W_stat_* | *p_­_* |
| Maize | $\delta$*ln*Er (YM) | 34 | 0.93 | 0.03 |
|  | $\delta$*ln*Cr (YM) | 34 | 0.94 | 0.08 |
|  | $\delta$*ln*Er (WM) | 34 | 0.94 | 0.05 |
|  | $\delta$*ln*Cr (WM) | 34 | 0.94 | 0.04 |
|  | $\delta$*ln*AI (WM) | 35 | 0.87 | <0.001 |
| Rice | $\delta$*ln*Er (IR) | 34 | 0.97 | 0.37 |
|  | $\delta$*ln*Cr (IR) | 34 | 0.94 | 0.07 |
|  | $\delta$*ln*Er (TR) | 34 | 0.95 | 0.09 |
|  | $\delta$*ln*Cr (TR) | 34 | 0.91 | 0.01 |
|  | $\delta$*ln*AI (ImpR) | 35 | 0.86 | <0.001 |
