# Supplementary material for: AI-imputed and crowdsourced price data show strong agreement with traditional price surveys in data-scarce environments
Source: PLoS One. 2025 Apr 8;20(4):e0320720. doi: 10.1371/journal.pone.0320720 (PMC11978078; doi:10.1371/journal.pone.0320720)
Supplement: Attachment — Submitted filename: Reviewer_Responses_JAdewopo.docx [file pone.0320720.s006.docx]

**Response Letter to Editor and Reviewer Notes**

**We thank the reviewers and editor for the extra diligence in reviewing this manuscript. The comments, suggestions, and notes have been very useful to enhance the content and clarity of the manuscript. Responses to each note are provided in blue texts below.

1. Please ensure that your manuscript meets PLOS ONE's style requirements, including those for file naming. The PLOS ONE style templates can be found at

https://journals.plos.org/plosone/s/file?id=wjVg/PLOSOne_formatting_sample_main_body.pdf and

https://journals.plos.org/plosone/s/file?id=ba62/PLOSOne_formatting_sample_title_authors_affiliations.pdf

All formatting requirements have been reviewed and addressed throughout the manuscript

2. Please note that PLOS ONE has specific guidelines on code sharing for submissions in which author-generated code underpins the findings in the manuscript. In these cases, we expect all author-generated code to be made available without restrictions upon publication of the work. Please review our guidelines at https://journals.plos.org/plosone/s/materials-and-software-sharing#loc-sharing-code and ensure that your code is shared in a way that follows best practice and facilitates reproducibility and reuse.

The guidelines for code sharing were duly followed, with DOI and associated notes published in the reproducibility package. We published the reproducibility package through WorldBank’s rigorous peer-review process, which includes exhaustive verification of the code and final independent report on the reproducibility of the outputs. The reproducibility package can be accessed [here](https://mcas-proxyweb.mcas.ms/certificate-checker?login=false&originalUrl=https%3A%2F%2Freproducibility.worldbank.org.mcas.ms%2Findex.php%2Fcatalog%2F137%3FMcasTsid%3D20893&McasCSRF=aa78c3543ef4a8e80aca8f5a228634bfae57b39545d0f32d49b9070ad6b9b955) . Also, we have created a new version or the code and posted in GitHub, updated with annotation that corresponds to the figure labeling within the paper. The url is https://github.com/PJNation/FoodPriceAnalytics

3. Please note that funding information should not appear in any section or other areas of your manuscript. We will only publish funding information present in the Funding Statement section of the online submission form. Please remove any funding-related text from the manuscript.

Done; Funding and other extraneous information has been removed

4. Thank you for stating the following financial disclosure:

"Funding by the Federal Ministry for Economic Cooperation and Development (BMZ, Germany) as part of the World Bank’s Food Systems 2030 (FS2030) Multi-Donor Trust Fund program (grants TF073570 and TF0C0728)

European Commission Joint Research Center (EC-JRC) Agropolis Foundation for the research mobility under Louis Malassis International Scientific Prize 2019"

Please state what role the funders took in the study. If the funders had no role, please state: "The funders had no role in study design, data collection and analysis, decision to publish, or preparation of the manuscript."

If this statement is not correct you must amend it as needed.

Please include this amended Role of Funder statement in your cover letter; we will change the online submission form on your behalf.

The role of funders is now indicated in the cover letter for your reference and update in the online submission

5. When completing the data availability statement of the submission form, you indicated that you will make your data available on acceptance. We strongly recommend all authors decide on a data sharing plan before acceptance, as the process can be lengthy and hold up publication timelines. Please note that, though access restrictions are acceptable now, your entire data will need to be made freely accessible if your manuscript is accepted for publication. This policy applies to all data except where public deposition would breach compliance with the protocol approved by your research ethics board. If you are unable to adhere to our open data policy, please kindly revise your statement to explain your reasoning and we will seek the editor's input on an exemption. Please be assured that, once you have provided your new statement, the assessment of your exemption will not hold up the peer review process.

As stated in the manuscript, the entire datasets are already published in public access repositories and the extracted data are included in the published reproducibility package. However, we have created a new version of the code and posted in GitHub, with annotation that corresponds to the figure labeling within the paper. The public access url is https://github.com/PJNation/FoodPriceAnalytics

6. We note that you have included the phrase “data not shown” in your manuscript. Unfortunately, this does not meet our data sharing requirements. PLOS does not permit references to inaccessible data. We require that authors provide all relevant data within the paper, Supporting Information files, or in an acceptable, public repository. Please add a citation to support this phrase or upload the data that corresponds with these findings to a stable repository (such as Figshare or Dryad) and provide and URLs, DOIs, or accession numbers that may be used to access these data. Or, if the data are not a core part of the research being presented in your study, we ask that you remove the phrase that refers to these data.

This phrase was inadvertently included in the paper and has been excluded because the full AI and crowdsourced data are included in the data repository. The average values are calculated from the respectively datasets in the open access reproducibility package.

7. We note that Figure 1 in your submission contain map/satellite images which may be copyrighted. All PLOS content is published under the Creative Commons Attribution License (CC BY 4.0), which means that the manuscript, images, and Supporting Information files will be freely available online, and any third party is permitted to access, download, copy, distribute, and use these materials in any way, even commercially, with proper attribution. For these reasons, we cannot publish previously copyrighted maps or satellite images created using proprietary data, such as Google software (Google Maps, Street View, and Earth). For more information, see our copyright guidelines: http://journals.plos.org/plosone/s/licenses-and-copyright.

We require you to either (a) present written permission from the copyright holder to publish these figures specifically under the CC BY 4.0 license, or (b) remove the figures from your submission:

a. You may seek permission from the original copyright holder of Figure(s) [#] to publish the content specifically under the CC BY 4.0 license.

We recommend that you contact the original copyright holder with the Content Permission Form (http://journals.plos.org/plosone/s/file?id=7c09/content-permission-form.pdf) and the following text:

“I request permission for the open-access journal PLOS ONE to publish XXX under the Creative Commons Attribution License (CCAL) CC BY 4.0 (http://creativecommons.org/licenses/by/4.0/). Please be aware that this license allows unrestricted use and distribution, even commercially, by third parties. Please reply and provide explicit written permission to publish XXX under a CC BY license and complete the attached form.”

Please upload the completed Content Permission Form or other proof of granted permissions as an "Other" file with your submission.

In the figure caption of the copyrighted figure, please include the following text: “Reprinted from [ref] under a CC BY license, with permission from [name of publisher], original copyright [original copyright year].”

b. If you are unable to obtain permission from the original copyright holder to publish these figures under the CC BY 4.0 license or if the copyright holder’s requirements are incompatible with the CC BY 4.0 license, please either i) remove the figure or ii) supply a replacement figure that complies with the CC BY 4.0 license. Please check copyright information on all replacement figures and update the figure caption with source information. If applicable, please specify in the figure caption text when a figure is similar but not identical to the original image and is therefore for illustrative purposes only.

The following resources for replacing copyrighted map figures may be helpful:

USGS National Map Viewer (public domain): http://viewer.nationalmap.gov/viewer/

The Gateway to Astronaut Photography of Earth (public domain): http://eol.jsc.nasa.gov/sseop/clickmap/

Maps at the CIA (public domain): https://www.cia.gov/library/publications/the-world-factbook/index.html and https://www.cia.gov/library/publications/cia-maps-publications/index.html

NASA Earth Observatory (public domain): http://earthobservatory.nasa.gov/

Landsat: http://landsat.visibleearth.nasa.gov/

USGS EROS (Earth Resources Observatory and Science (EROS) Center) (public domain): http://eros.usgs.gov/#

Natural Earth (public domain): http://www.naturalearthdata.com/

We created the boundary maps from the scratch with open-access country (Nigeria) and Africa-continent boundaries which were obtained from [www.gadm.org](http://www.gadm.org). We checked and confirmed that Figure 1 (Map of study area) does not contain any additional satellite imagery or copyrighted materials. The source allows for use and publishing of maps and shapefile data in PloSOne as stated in this license permission clause - https://gadm.org/license.html. Our georeferenced price datapoints were used to create the location point shapefile layers that were included in the first map (Fig 1). The hash-line shading in the map is mere symbology of the focal states and no other spatial layers or maps are embedded. The grey background was a mere color-fill, not a basemap, and it does not convey any information. Additional information regarding the source of the shapefile has been added to the Figure caption.

8. Please include captions for your Supporting Information files at the end of your manuscript, and update any in-text citations to match accordingly. Please see our Supporting Information guidelines for more information: http://journals.plos.org/plosone/s/supporting-information.

The Supporting information section has been duly updated as recommended, following PloSOne guideline.

9. Please review your reference list to ensure that it is complete and correct. If you have cited papers that have been retracted, please include the rationale for doing so in the manuscript text, or remove these references and replace them with relevant current references. Any changes to the reference list should be mentioned in the rebuttal letter that accompanies your revised manuscript. If you need to cite a retracted article, indicate the article’s retracted status in the References list and also include a citation and full reference for the retraction notice.

Reference list has been reviewed for completeness; There is no instance of retracted article so no changes were made on the list to warrant additional notes

Reviewer #1: Reviewer Comment

Title: Validation of AI and Crowdsourced Food Price Data for Real-Time Monitoring in Fragile Contexts

Specific Comments

1. Abstract

The abstract is well-written and presents important findings.

• Explicitly state the research gap the study addresses. For example, mention the limitations of current price monitoring methods and the specific contributions of this study

Thank you for the suggestion. The abstract has been revised to further bring the research problem to fore, within the word count limit [L32-33] and [L36-41]

• Include the statistical methods used for correlation analysis, such as the Pearson correlation coefficient.

The statistical method is now included in the abstract, as recommended

2. Introduction

• Lines 64-87: The challenges section is comprehensive but could be summarized to focus on the key points.

We deleted [L67-69] and [L79-82] to shorten the paragraph, and revised the paragraph slightly to sharpen the focus

• Lines 88-105: The introduction to data crowdsourcing and AI is detailed. Streamline this section to focus on the relevance of these methods to the study's objectives.

We intended to provide relevant contexts that can guide the understanding of the audience, however, following the reviewers’ comment, we have deleted [L99-101]. This narrative provides a prelude to justify data innovation for food system

• Lines 139-150: The study's objective should be clearly highlighted, summarizing the validation effort's key goals and significance.

Thank you. This section has been revised to clearly call out the objective. Additional texts were added to highlight the significance of the analysis as well.

3. Methods

• Lines 155-177: Consider breaking the paragraph into smaller sections to improve readability. For example, separate discussions on socio-economic context, agricultural production, and market dynamics This is a helpful suggestion. We have split the context into 2 paragraphs for better readability; The first part addresses the socio-economic context while the second part addresses the Agricultural production/market context.

We presume that this is

• Lines 177-182: The map description is clear. Ensure it is referenced appropriately within the text to guide readers' understanding of the study area's scope.

Thank you for confirming that the map description is clear. We checked and confirmed that it is reference appropriately within the text as well.

4. Results

• Provide concise summaries at the beginning of each subsection (e.g., Ground truth vs. Crowdsourced Prices, crowdsourced vs. AI-Estimated Prices) to orient readers to the focus and findings of that particular analysis.

Thank you. For clarity, we have revised the beginning of the sub-sections to point readers to the summary note before delving into details.

• Emphasize the practical implications of findings, such as how the coherence between datasets (e.g., crowdsourced vs. enumerator) at different time intervals impacts the reliability of high-frequency surveillance in food price monitoring.

This is definitely an important note which rightly fits under the discussion and conclusion section, as already presented there. To avoid redundancy, we retain the rendition of the results with minimal modification.

• Consider discussing outliers or unexpected trends observed in the data, as this can provide insights into the limitations or strengths of each data source.

Indeed! We appreciate this valuable suggestion and we have added texts into the discussion section regarding outliers and their connotation for future research interests

5. Discussion

• Clearly link each discussion point directly to the study objectives to maintain focus.

This is a helpful note. We have revised the discussion section to improve the flow and focus for the audience.

• Describe trends and patterns highlighted by figures and relate them back to broader discussion points on data reliability and applicability.

Per note above, we revised and updated texts to bring focus to data pattern.

Overall Recommendation

The study is valuable, and warrants publication after the above concerns are addressed.

Reviewer #2: The paper can be accepted subject to the following modification:

1. The data interpretation should be accompanied with some suitable visualisations. This should be considered within the section Materials and Methods. It would be interesting to see how the variables are distributed over different geographic regions, market areas etc.

We thank the reviewer for this comment. We conducted extra analysis of time-lagged correlations between AI-generated and crowdsourced prices, and included 2 new maps (Fig 6b and Fig 6c), a new chart (Fig 5), and additional texts in L235 – 241. Regarding “how the Variables are distributed”, please note that the relevant visualizations are already presented in the article, including figure 1 which shows the geographic distribution of the crowd-submitted datapoints, and georeferenced locations of the other data sources, while Figures 6a and 6d shows the relationship of compared datasets at disaggregated admin1 (State) level and between market segments. The new map (Fig 6b) further presents spatial distribution of the attribute average monthly price for Maize, while second new map (Fig 6c) shows visualization of the disaggregated correlation at lower administrative levels (admin2) to showcase the spatial consistence of the relationship. We are also intentional to avoid redundant charts/maps or texts that may unduly stretch the length of the article.

2. Assumptions regarding the data prior to correlation analysis should be explained clearly. Time lagged relationship between the crowd sourced data and the AI generated data should also be identified using cross correlation techniques. The visualisations shown as per the comment number 1 should also be helpful in indicating the time lagged properties of the data.

This suggestion is duly noted and we have included additional texts regarding our assumptions on the datasets prior to the analysis, including individual independent distribution (IID) of datasets from the different sources (L223-225; & L508-514). As noted above, we have also implemented additional analysis and included a graphical chart to show lagged correlation properties of the data, and the correlation at lower admin2 level.
