# Supplementary material for: AI-imputed and crowdsourced price data show strong agreement with traditional price surveys in data-scarce environments
Source: PLoS One. 2025 Apr 8;20(4):e0320720. doi: 10.1371/journal.pone.0320720 (PMC11978078; doi:10.1371/journal.pone.0320720)
Supplement: Attachment — Submitted filename: Response to Reviewers.docx [file pone.0320720.s007.docx]

**Dear Reviewer/Editor,**

Thank you very much for your thorough review of our paper and the insightful suggestions you provided. We have carefully considered each of your comments and made revisions throughout the manuscript, which we believe have significantly enhanced its quality. Your feedback has been instrumental in improving the clarity, depth, and overall presentation of our work.

We have structured our response as follows: we first provide a single response to each of the 14 groups of comments for a more streamlined overview. This is followed by specific point-by-point answers to each of the 141 individual comments, presented in a comment/response format. To ensure clarity and conciseness, we sometimes use shortened versions of your comments that reflect our understanding and the actions we took. This approach allowed us to address all feedback comprehensively while keeping the response letter concise, as including the full text of each comment would have resulted in a response letter more than twice the length of our article.

We hope that our revisions and the detailed explanations provided meet your expectations, and we sincerely appreciate your time and effort in reviewing our manuscript.

Thank you once again for your valuable feedback.

**Title**:

*Response:*

Thank you for your detailed feedback and suggestions on the title. We have revised it to: “AI-imputed and crowdsourced price data show strong agreement with traditional price surveys in data-scarce environments” to better reflect the focus and contributions of our study. In response to your comments, we made several key revisions. We specified the AI methods used, emphasizing the use of hybrid machine learning models, and clarified the crowdsourcing approach, particularly the smartphone-based data collection process. Additionally, we included more information about how ground truth data was collected through enumerators. To avoid potential confusion, we removed terms like "fragile" and "high-frequency," ensuring that the title remains both concise and precise, while avoiding any redundancy. These changes aim to enhance the clarity and readability of our manuscript, making it more accessible to the journal's audience.

1. **Ambiguity in Terminology:**

- *Comment:* The title includes the term "Artificial Intelligence" (AI) but does not specify what type of AI or the particular methods used (e.g., machine learning, neural networks). Providing more detail could clarify the technological approach employed.
- *Response:* We have included additional information about the type of AI used in the manuscript [lines 237-266: “The imputation process employs hybrid machine learning models that integrate local relationships within specific regions of the feature space, akin to the concept of local receptive fields in convolutional neural networks”]. Our study explores the feasibility of AI-imputation methods broadly, using publishedAI-imputed price datasets; therefore, our revised title captures this broader focus without specifying a single approach.

2. **Vague Description of "Crowdsourcing Approach":**

- *Comment:* The term "crowdsourcing approach" is broad and could benefit from more specificity. Detailing the type of crowdsourcing method used (e.g., survey-based, app-based submissions) would give readers a clearer understanding of the data collection process.
- *Response:* We have clarified the details in the manuscript [lines 213-236: “. The volunteers were remotely invited and onboarded to submit commodity price data, including maize and rice, through the ODK app”]. While most crowdsourcing is conducted through mobile devices, we felt using the term "mobile" in the title might cause confusion with other mobile data types, like location data, which differ in context and methodology.

3. **Lack of Detail on "Groundtruth Data":**

- *Comment:* The title mentions "groundtruth data" but does not indicate how this data was collected or its source. Including information about the type or source of groundtruth data could make the title more informative and precise.
- *Response:* We have added more details on the collection of ground truth data in the text [lines 161-167], describing the role of enumerators and their submission methods.

4. **Inconsistency in Terminology:**

- *Comment:* The phrase "validated with groundtruth data" suggests a comparison or verification process. To improve clarity, the title could specify what aspects of the data were validated and how this validation supports the findings.
- *Response:* We have revised the title to remove this ambiguity and ensure a clearer representation of the study’s approach and findings.

5. **Generalization of "Fragile Context":**

- *Comment:* The term "fragile context" is used without explanation. Providing more context about what constitutes a fragile context in this study (e.g., economic instability, conflict areas) would help in understanding the specific relevance and application of the research.
- *Response:* We have revised the title to exclude the term “fragile” and made changes in the abstract and throughout the manuscript to better explain the specific context [e.g., “northern Nigeria, a region affected by conflict, food insecurity, and data scarcity”]. We recognize that "fragile" is a term with a specific meaning in certain circles but may not be immediately clear to all readers.

6. **Potential for Overstatement:**

- *Comment:* The title might overstate the significance of the findings by using terms like "high-frequency" and "validated." If the validation process or the frequency of data collection is not as comprehensive as implied, this could lead to misleading interpretations.
- *Response:* We have removed the terms "high-frequency" and "validated" from the title to avoid any potential overstatement.

7. **Complexity and Length:**

- *Comment:* The title is somewhat long and complex, which might make it less accessible. A more concise title that still conveys the main aspects of the study could be more effective in attracting and retaining reader interest.
- *Response:* The revised title aims to balance conciseness with clarity, emphasizing the study’s focus while keeping the length in check.

8. **Repetition of Terms:**

- *Comment:* The title repeats the concept of validation and the use of alternative data sources. Streamlining these elements could enhance readability and focus.
- *Response:* We have streamlined the title to avoid repetition and focus on the key aspects of our study.

**Abstract:**

*Response:*

Thank you for your thorough review and constructive feedback on the abstract. We have fully revised it to improve readability, clarity, and detail, addressing the specific issues you raised. Key revisions include adding more information on how the AI and crowdsourcing methods were applied, justifying our choice of validation methods like Pearson’s correlation and paired t-tests, and providing clearer details on the validation process itself, including how ground truth data was collected and analyzed. We also streamlined the abstract to remove repetitive statements and enhance focus. Additionally, we emphasized the practical implications of our findings, particularly in regions with limited capacity for conventional surveys, and highlighted the relevance of our study to northern Nigeria and similar contexts. We also resolved grammatical and typographical issues identified, providing both a clean and tracked version for your review. We believe these changes have significantly strengthened the abstract, and we appreciate your feedback in helping to improve our manuscript.

1. **Ambiguity in Data Innovation Details:**

- *Comment:* The abstract mentions data innovations such as AI and crowdsourcing but does not clearly explain how these methods were specifically applied or what distinguishes them from conventional methods.
- *Response:* We have revised the abstract to include more details about the specific application and justification of the methods. Additionally, we have simplified the language for improved clarity, focusing on the distinctive aspects of AI and crowdsourcing compared to traditional methods.

2. **Methodological Concerns:**

- *Comment:* While the abstract states that Pearson’s correlation and paired t-tests were used for validation, it does not provide information on the rationale for choosing these specific methods or how they were applied.
- *Response:* We have revised the abstract to briefly justify the choice of these methods [lines 30-38]. Further details on the rationale and application of the methods are now included in the main text, including supporting information.

3. **Insufficient Description of Validation Process:**

- *Comment:* The abstract notes the validation of AI-generated and crowdsourced data against ground truth data but lacks details about the validation process.
- *Response:* We have updated the abstract to include more specific details on the validation process, such as how the ground truth data was collected, the time periods compared, and key item pair-level validation results. These additions aim to enhance the credibility of the findings.

4. **Repetitive Statements:**

- *Comment:* The abstract contains repetitive statements regarding the need for validation and the importance of real-time intelligence on food affordability.
- *Response:* The abstract has been revised to improve conciseness and eliminate redundancies. We have streamlined the text to ensure a sharper focus on the core findings and contributions.

5. **Limited Insight into Findings:**

- *Comment:* Although the abstract highlights the comparability of AI and crowdsourced prices, it lacks insight into the practical implications of these findings.
- *Response:* We have revised the latter part of the abstract to address the practical implications of our findings. This now emphasizes how our results provide crucial evidence to support the use of alternative data sources in decision-making processes, particularly in contexts with limited capacity for conventional price surveys [lines 39-43].

6. **Generalization of Results:**

- *Comment:* The abstract reports high correlation values and similar inflation trends but does not discuss potential limitations or contexts where these methods might be less effective.
- *Response:* We have adjusted the concluding sentences of the abstract to indicate the contexts in which these methods are most applicable. Specifically, we highlight their usefulness in subnational regions with limited monitoring capacity and where high-frequency local data is essential.

7. **Lack of Contextual Relevance:**

- *Comment:* The abstract emphasizes high-frequency monitoring in fragile contexts without providing sufficient background on the relevance of the study's specific focus on northern Nigeria.
- *Response:* We have provided additional context at the end of the abstract, explaining the relevance of the northern Nigeria setting and its broader implications for similar regions. This contextual relevance underscores why the study’s findings matter beyond this particular case.

8. **Typographical and Grammar Issues:**

- *Comment:* There are minor grammatical errors and typographical issues in the abstract, such as “addresson” instead of “address,” and “wWe” instead of “We.”
- *Response:* We have thoroughly copy-edited the paper, and these issues have been resolved. The examples you mentioned were artifacts of track changes, and we have now provided both a track changes version for transparency and a clean version for ease of reading.

**Introduction section:**

*Response:*

Thank you for your thorough review of the introduction. We have rewritten this section to be more focused on the core issues, reducing extraneous statistics, streamlining the text, and adding necessary details. Specifically, we removed unnecessary statistics, focusing instead on targeted data points that directly support the study’s focus. We clarified the link between data scarcity and development challenges, highlighting the critical need for timely food price monitoring in fragile contexts and emphasizing our study's unique contribution in this area. Additionally, we shortened the introduction to ensure a tighter focus on the study's objectives, included more specific details on AI and crowdsourcing methods, and provided a clearer explanation of the term “fragile context” with a preference for more precise language where possible. We also refined the presentation of our research question, removed redundant information, and reduced technical jargon for accessibility. Finally, we improved the transition between the general background and the study’s specific focus, ensuring a more logical flow. We believe these revisions have enhanced the clarity, focus, and overall presentation of the introduction, and we appreciate your feedback, which has greatly improved this section of our manuscript.

1. **Overuse of Statistics Without Context:**

- *Comment:* The introduction contains extensive statistics on food insecurity and poverty but lacks specific context or direct relevance to the study's focus.
- *Response:* We have revised the introduction to remove most extraneous statistics and have presented remaining data in a more targeted way. For instance, we now directly mention the item pairs for which statistics are presented, instead of providing a range for multiple comparisons. These adjustments help ensure that the statistics serve to directly support the study’s focus. Similar edits have been made throughout the paper for consistency.

2. **Lack of Focus on the Core Problem:**

- *Comment:* The introduction could benefit from a more focused discussion on why monitoring food prices in fragile contexts is critical and how the study uniquely addresses this issue.
- *Response:* We agree, and we have clarified the connection between data scarcity and development challenges, citing recent quantitative studies on the subject. The revised text highlights food security as a specific issue requiring timely data, emphasizing the potential for the price data to support monitoring of rapid food crisis escalation. The unique focus and value of our study are now clearly outlined in lines 61-103.

3. **Complexity and Length:**

- *Comment:* The introduction is lengthy and dense, covering multiple aspects of food insecurity, data challenges, and innovations in data methods.
- *Response:* We have shortened and streamlined the introduction, ensuring a tighter focus on the study’s objectives and the most relevant background information.

4. **Insufficient Detail on Data Innovations:**

- *Comment:* The introduction discusses crowdsourcing and AI but lacks specific details on their application within the study.
- *Response:* We have introduced more details on the crowdsourcing and AI method in lines 104-115, and have also expanded on this in the Methods section (lines 161-267) to provide a clearer understanding of the implementation of these approaches.

5. **Overemphasis on General Background Information:**

- *Comment:* The introduction includes extensive background information that could be condensed.
- *Response:* We have significantly condensed the introduction to focus on the information directly relevant to the study. Background information has been reduced to ensure a more direct rationale for the study's focus on validating innovative data methods.

6. **Ambiguous Explanation of "Fragile Context":**

- *Comment:* The term "fragile context" is used without a clear definition.
- *Response:* As mentioned in previous responses, we have clarified this term throughout the paper and de-emphasized its use in favor of more specific descriptions where possible.

7. **Lack of Clear Research Questions or Objectives:**

- *Comment:* The introduction outlines broad issues but does not clearly state the study’s research questions or objectives early enough.
- *Response:* We have revised the introduction to sharpen the focus, clearly stating the research objectives in lines 104-115. Some technical details have been moved to the Methods section to improve the flow and focus of the introduction.

8. **Redundancy and Repetition:**

- *Comment:* Some points, such as the limitations of traditional data collection and the potential of new data methods, are repeated.
- *Response:* We have reviewed the introduction for redundancy and have made edits to remove repeated points. This has resulted in a more concise and impactful presentation.

9. **Technical Jargon Without Explanation:**

- *Comment:* The introduction uses technical terms without sufficient explanation.
- *Response:* We have removed jargon that is not critical for understanding the study’s focus and have ensured that essential terms are introduced later in the paper with appropriate explanations in the Methods section.

10. **Unclear Transition to Study’s Specifics:**

- *Comment:* The transition from general discussion to the study’s specific focus is abrupt.
- *Response:* We have streamlined the introduction to follow a logical flow: first outlining the challenge of obtaining timely, actionable data, then discussing the innovations around crowdsourcing and AI imputation, stating the study’s objective of assessing the reliability of these methods, and concluding with the relevance of the findings. This improved structure enhances the coherence and readability of the introduction.

***Materials and Methods Section:***

*Response:*

Thank you for your detailed review of the Materials and Methods section. We have made several revisions to address your suggestions, improving the flow and aligning it better with the Data description. We clarified the rationale for selecting the study area, specifying our criteria for choosing the three states in northern Nigeria (Lines 141-148). We also connected the historical context more directly to the study's focus on price data. To clarify the overlap between data sources, we revised the description of how regions were selected based on available data from all three sources. Additional details were provided for the data collection methods, including separate explanations for each data source with dedicated headers (Lines 181 - 267). We also simplified the discussion around market types, by refocusing on “market segments” for clarity. References to “price transfer” were removed to keep the focus on the study’s core scope. Further, we expanded on the methods used by the AI algorithms and crowdsourcing platforms, offering more detail on data preprocessing (Lines 269-292). We improved the description of the validation process (including sensitivity and formal equivalence tests), outlining it more clearly to highlight the rigor of our approach (Lines 371-537). Lastly, we adjusted the description of Figure 1 to better integrate with the text and added discussions on potential biases related to market selection. These revisions aim to enhance clarity and ensure that readers gain a better understanding of the study’s methods and scope.

1. **Lack of Justification for State Selection:**

- *Comment:* The section does not provide a clear rationale for selecting these specific three states in northern Nigeria.
- *Response:* We appreciate this observation and have revised the methods section to clearly justify the study area selection. See Lines 141-148, which state: “The study area was selected based on specific criteria for validating alternative data sources …[ctd]”.

2. **Unclear Relevance of Historical Context:**

- *Comment:* The historical context of insecurity and agricultural practices is mentioned but not explicitly linked to the study’s objectives.
- *Response:* Thank you for this feedback. We have revised the introduction section to better connect the historical context with the study’s focus on price data.

3. **Ambiguity in Data Overlap Description:**

- *Comment:* The description of the overlap between World Bank’s RTP data and crowdsourced submissions is vague.
- *Response:* We have revised the data description to clarify the independence and overlap of the data sources. The study opportunistically focuses on regions where prices are available for all three data sources (Lines 141-170). We note that the challenges in the selected study area have broader applicability.

4. **Insufficient Detail on Data Collection Methods:**

- Comment: The section lacks detailed information on how crowdsourcing and AI estimation methods were implemented.
- *Response:* We have included more details, presenting each of the three data sources with dedicated headers for clarity (Lines 181-267). We also referenced previously published work to provide further information on process and quality control measures without adding redundancy.

5. **Inconsistent Information on Market Types:**

- Comment: There is repetition and inconsistency in describing market types and their characteristics.
- *Response:* We removed references to “market types” in this section to avoid confusion. The analysis examines price correlation at the “market segment” level, not by “market type,” since AI-imputed data represents only the “Retail” market segment.

6. **Inadequate Explanation of Price Transfer Influence:**

- Comment: The influence of vendors owning multiple stores on spatial price transfer is mentioned but not elaborated.
- *Response:* To avoid distracting the reader, we have removed references to “price transfer,” as its assessment is beyond the scope of this study.

7. **Lack of Detail on AI and Crowdsourcing Methods:**

- Comment: The section does not sufficiently describe the specific methods used by the AI algorithms and crowdsourcing platforms.
- *Response:* As noted earlier, we have revised this section to provide more detailed information about AI models, data preprocessing, and crowdsourced data integrity (Lines 181-267).

8. **Limited Description of Validation Process:**

- Comment: The process of how ground truth prices were compared to AI-generated and crowdsourced prices is not adequately described.
- *Response:* We have improved clarity regarding the validation process by laying out the validation steps in Lines 258-267, ensuring a clear understanding of the study’s rigor.

9. **Map and Data Source Details:**

- Comment: The description of Figure 1 and the data sources is somewhat disjointed.
- *Response:* We have revised the figure title for better integration with the text and clarified that GADM is the source of the shapefiles used to build the map.

10. **Potential Biases in Market Coverage:**

- *Comment:* The section does not address potential biases in market selection or their impact on the study results.
- *Response:* We have addressed this point by discussing potential biases in Lines 196-210. We also added content to the discussion section to inform readers of the potential limitations regarding market locations.

**Data Description section:**

Thank you for your detailed review of the Data Description section. We have made several revisions to improve clarity and detail. We added an introductory paragraph to explain the integration of datasets (Lines 162-267) and included additional details on quality control measures for crowdsourced data, referencing previous work to avoid repetition. To clarify metadata use, we removed the term “unpublished metadata” and specified how submitter IDs were used (Lines 196-202). We expanded on the training and protocols for enumerators to address potential biases (182-189) and refined descriptions of data processing methods, replacing vague terms like "wrangled." The term “standardized” was clarified to better explain price recalculations using conversion factors. We also highlighted the study’s focus on validating AI-imputed prices against ground truth data (Lines 171-180). Additionally, we justified the selection of rice and maize based on their presence in both datasets (Lines 141-148) and clarified the description of market segments and coverage, supported by Figure 1. These revisions enhance the clarity and comprehensiveness of the data description.

1. **Lack of Clarity on Data Integration Process:**

- *Comment:* The section briefly mentions coupling and wrangling datasets but lacks specific details on the methodology used to integrate data from different sources. Information on how inconsistencies and data mismatches were resolved is missing, which raises concerns about the reliability of the combined dataset.
- *Response:* We have revised the data description section to provide better clarity. We inserted an introductory paragraph to explain how the datasets relate to each other [Lines 162-180].

2. **Ambiguity in Crowdsourcing Data Collection:**

- *Comment:* The description of crowdsourced data collection lacks detail on how data quality was ensured, such as the verification of volunteer submissions.
- *Response:* We have added more details and restructured the section to include brief descriptions of quality control measures. We also refer readers to our published article for further details to maintain focus on this study and avoid repetition [L214-237]

3. **Insufficient Information on Metadata:**

- *Comment:* The role of metadata, such as submitter IDs, is not clearly explained in the context of data validation.
- *Response:* To avoid confusion, we have excluded the term “unpublished metadata” and instead clarified that pre-assigned IDs were used to differentiate data submitted by crowd volunteers from those submitted by enumerators (L196-202).

4. **Potential Bias in Enumerator Data:**

- *Comment:* Details on how enumerator data might be influenced by personal biases or logistical challenges are lacking.
- *Response:* We have added more information on the training process, data collection protocols, and measures to ensure consistency in the methods section (L200-210).

5. **Inadequate Explanation of Data Wrangling:**

- *Comment:* The term "wrangled" is used without elaboration on the data cleaning and transformation techniques.
- *Response:* We have revised this section, removed the vague term, and provided more precise descriptions of how the data was processed.

6. **Unclear Standardization Process:**

- *Comment:* The explanation of price standardization across market segments is vague.
- *Response:* We have removed references to “standardized” and clarified that prices were recalculated in local currency based on conversion factors of measurement units.

7. **No Mention of Data Validation for AI-Estimated Prices:**

- *Comment:* There is no information on how the accuracy of AI-estimated prices was validated against ground truth data.
- *Response:* We clarified that the main purpose of this paper is to validate AI-imputed prices against independent ground truth price data, and this paper represents the first attempt at external validation of an operational AI-imputed price data pipeline (L171 – 180). The internal validation of the AI-imputed prices is addressed in L258-267.

8. **Ambiguity in AI Methodology Description:**

- *Comment:* The AI methodology description is technical but lacks clarity on how models address local price dynamics.
- *Response:* We revised the methods section to better highlight the AI imputation procedure, citing our previous publication that provides a more comprehensive explanation.

9. **Limited Justification for Commodity Selection:**

- *Comment:* The rationale for focusing on rice and maize over other commodities is not provided.
- *Response:* We have revised the text to clarify that rice and maize were selected because they are the only commodities present in both alternative price datasets (Lines 141-145). We also noted that the commodities available in RTP data are a function of data availability in the primary survey source (WFP), which reflects a prioritization stemming from their operational use in humanitarian responses. The foods are typically key staples in a region.

10. **Generalization of Market Coverage:**

- *Comment:* The description of market types and coverage is too generalized.
- *Response:* We have excluded references to “Market types” to focus more directly on market segments (Lines 229-231). Also, we revised the text to indicate the number of markets covered within each dataset, and Figure 1 provides details on the spatial distribution of prices across more than 150 unique market locations.

**Statistical Analyses section:**

Thank you for your detailed feedback on the Statistical Analyses section. In response, we have added justifications for our chosen methods, clarified the impact of outlier detection, and addressed assumptions in our statistical tests. We improved the explanation for time-lag analysis (which was requested by the previous review) and the use of centroids, expanded on the limitations of correlation metrics, and emphasized practical significance alongside statistical results. We also refined the description of software use, discussed the effects of data aggregation, and enhanced the interpretation of results in the context of our study. These revisions aim to provide a clearer and more rigorous presentation of our statistical approach.

1. **Limited Description of Statistical Methods:**

- *Comment:* The section lacks detailed explanations of why specific statistical techniques were chosen, such as Pearson’s correlation and paired t-tests.
- *Response:* Based on this note, we have revised the section to provide more detailed justifications for using correlation tests, emphasizing how they align with our study’s objectives (L293-537).

2. **Potential Issues with Outlier Detection:**

- *Comment:* The use of Tukey's outlier detection method is not discussed in terms of its impact on the results and the integrity of the data.
- *Response:* Thank you for highlighting this point. We have added text to explain that outliers had no significant impact on average prices but were removed as a routine procedure to ensure analysis integrity (L286-292).

3. **Assumption of Independence:**

- *Comment:* The assumption of independence between datasets may oversimplify the relationship between crowdsourced and enumerator-submitted data.
- *Response:* Thank you. We have clarified in the data description that enumerators operated independently from crowd volunteers. The focus of this paper is to explore correlations and assess whether alternative data sources are comparable to enumerator-submitted data.

4. **Lack of Justification for Time-Lag Analysis:**

- *Comment:* The rationale for evaluating time-lagged relationships is not clearly explained.
- *Response:* We have added further explanation to justify the time-lag analysis, noting its relevance for understanding correlations between AI-estimated and crowdsourced prices over time (Lines 371-380). We thank the previous reviewer for suggesting this analysis.

5. **Methodological Concerns with Centroid Calculation:**

- *Comment:* The centroid calculation to assess correlations may introduce inaccuracies, and its impact is not discussed.
- *Response:* We have added text discussing how selecting the closest AI market location might impact correlation results, providing additional context for the analysis (Lines 381-389

6. **Inadequate Detail on Correlation Metrics:**

- *Comment:* The discussion of correlation coefficients and their limitations is basic, lacking context on their application in this study.
- *Response:* We have added a discussion on the limitations of correlation metrics (Lines 293 -340), further noting that this justifies the use of paired t-tests (and other tests) as an additional means of analysis (L341-370)

7. **No Mention of Assumptions in Statistical Tests:**

- *Comment:* The section does not address the assumptions underlying statistical tests like the paired sample t-tests.
- *Response:* We have included a discussion on the assumptions of normality and equal variances for the t-tests, ensuring transparency about the conditions of the tests (Lines 341-370).

8. **Potential Over-Reliance on Statistical Significance:**

- *Comment:* The focus on p-values may overlook the practical significance of the results.
- *Response:* This point has been considered in our overall revisions, with a greater emphasis placed on discussing the practical implications of the findings.

9. **Ambiguity in Statistical Software Usage:**

- *Comment:* The reference to "relevant R packages and functions" is vague.
- *Response:* We have revised the statement, moving it to the first paragraph of the section. Our R code is available for public access and all the packages used are included; We also provide link to a reproducibility package with the doi for ease of access L294 - 299.

10. **Lack of Discussion on Data Aggregation:**

- *Comment:* The effects of aggregating data to different time intervals (daily, weekly, monthly) are not discussed.
- *Response:* We agree that aggregation can influence correlation results, as observed in our analyses. We have added a discussion on how temporal aggregation improved correlations, highlighting its impact (L78-85; L282-285; L838-842).

11. **Unclear Explanation of Results Interpretation:**

- *Comment:* The section lacks information on how the results contribute to the study’s conclusions.
- *Response:* We have restructured the discussion section to better connect the statistical results with the study's objectives and conclusions, providing more insight into their implications (L 390-537). Specifically, we provide relevant hypothesis to guide the interpretation.

**Results section:**

Thank you for your valuable feedback on the Results section. In response, we have clarified the presentation of statistical analyses and included additional details on interpreting statistical measures. We refined the focus of the results to emphasize key findings and simplified the presentation of AI-estimated prices. The rationale for selecting specific commodities was better explained, and we integrated findings across different analyses for a cohesive presentation. We addressed potential data limitations and provided more detail on the supplementary materials, while adding comparisons to existing literature to contextualize our findings. These revisions aim to enhance the clarity, depth, and relevance of the Results section.

1. **Lack of Detailed Statistical Analysis:**

- *Comment:* The results lack detail on how statistical values are interpreted and their significance. A more thorough presentation, including measures of uncertainty and confidence intervals, would improve clarity.
- *Response:* We revised the results section to first present a general summary and then detail findings for each correlation analysis (Lines 538-793). Additional explanations on interpreting correlation coefficients and other statistical measures are included in the methods section (Lines 293-537).

2. **Inadequate Comparison of Sub-Types:**

- *Comment:* The comparison of commodity sub-types is not sufficiently detailed, leading to possible confusion about their relevance.
- *Response:* To enhance clarity, we revised the section and Table 1 to present results for maize and rice subtypes; Also, we clarified that rice sub-types is not fully defined for AI-imputed prices because they are only available as “imported Rice” which may include different brands beyond what’s covered in crowdsourced rice prices (L540-595).

3. **Issues with Data Aggregation:**

- *Comment:* The conversion of units to a per-kilogram basis is not discussed in terms of its impact on accuracy, such as discounts for bulk quantities.
- *Response:* We removed references to conversion or discounts from the results section, noting that these are more appropriate for the discussion section. Standardized conversion was applied to derive the price per kilogram; The impacts of potential discount on bulk quantities are discussed. Generally, we assume that these artifacts are minor and have no impacts on data trends, based on random distribution of the reported quantities and the averaging out of the monthly aggregates.

4. **Confusing Presentation of AI-Estimated Prices:**

- *Comment:* The rationale for using specific price metrics, like closing prices, is unclear.
- *Response:* We clarified the choice of “closing prices” and moved this explanation to the Methods section for improved flow and readability (Lines 238-267).

5. **Unclear Justification for Focus Areas:**

- *Comment:* The rationale for focusing on certain commodities over others is not well explained.
- *Response:* We revised the entire section to present the results on all commodities, including subtypes as shown in L541 - 598; More results for rice are presented as supporting information.

6. **Lack of Integration of Findings:**

- *Comment:* The results from different analyses are presented separately without integration, making it harder to understand the overall implications.
- *Response:* We improved the flow and connection between sections, with an integrated presentation of findings, as shown on the summary in Table 1. Further results for tests of sensitivity and equivalence are shown on Table 2 (L780 -796)

7. **Limited Discussion of Nuanced Aspects:**

- *Comment:* There is little exploration of how factors like sub-national geography and market segments influence the results.
- *Response:* We streamlined the focus on administrative-level geography (Admin1 i.e. States) and market segments, adding results that show differences in price relationships based on this both dimensions (L697-741).

8. **Insufficient Detail on Data Quality and Limitations:**

- *Comment:* Potential quality issues and limitations of data sources, such as biases in crowdsourced data, are not sufficiently addressed.
- *Response:* We added a new paragraph discussing data limitations (Lines 558-592) and revised earlier sections to highlight potential biases and their effects (Lines 214-220).

9. **Sparse Explanation of Supplementary Material:**

- *Comment:* There is little detail on the supplementary material and how it supports the main findings.
- *Response:* We have added texts to connect the supporting information to the main results (Lines e.g. L76-481; L548-550).

10. **Missing Contextualization of Findings:**

- *Comment:* The results lack sufficient context or comparison to existing literature.
- *Response:* We revised the discussion section for improved readability and added comparisons to existing literature to better contextualize our findings (e.g. L885-905).

**Figure 2 results section:**

Thank you for your valuable feedback on the Figure 2 Results section. We have made several revisions to improve clarity and context. We added more detailed explanations for interpreting correlation coefficients and adjusted the presentation to avoid confusing references to variability. The emphasis on p-values has been balanced with a discussion of practical implications, and we expanded on the impact of data noise at different time resolutions. We clarified terms like "ground truth" and improved the explanations in the figure legend for better readability. We also provided a more balanced focus on AI-estimated prices and refined the discussion on temporal trends and statistical metrics. Additionally, we addressed potential biases in the data sources and clarified the impact of conversion methods. These revisions aim to enhance the comprehensiveness and clarity of the results presented in Figure 2.

1. **Lack of Context for Correlation Coefficients:**

- *Comment:* Correlation coefficients are presented without sufficient context, making it unclear how these values compare to benchmarks or their practical significance.
- *Response:* The figure legend has been revised for greater clarity, providing more context for interpreting the coefficients (Lines 567-577). Also, as noted previously, we have provided a contextualization of the correlation coefficients in the methods section.

2. **Inconsistent Data Presentation:**

- *Comment:* There is a lack of clarity on how variability in prices impacts overall correlation, particularly when comparing results at different time intervals.
- *Response:* We revised the text to avoid confusing references to "variability" and added further explanation regarding the effects of daily, weekly, and monthly price aggregation (L615-623).

3. **Overemphasis on Statistical Significance:**

- *Comment:* Emphasis on p-values might obscure the practical significance of the findings.
- *Response:* We have reduced references to statistical significance and added discussion of the practical implications of the results for stakeholders (L743-779; ).

4. **Limited Discussion on Data Noise:**

- *Comment:* The impact of data noisiness at daily and weekly levels is not fully explored.
- *Response:* We addressed this by expanding the discussion on how data noise affects the validity of correlations and the reliability of results (e.g. L833-855).

5. **Ambiguous Variable Definitions:**

- *Comment:* Terms like "ground truth" and "crowdsourced" are used without clear definitions.
- *Response:* We have further defined these terms in the methods section to ensure clarity and prevent confusion (L162-180).

6. **Inadequate Visualization Explanation:**

- *Comment:* Figures 2B and 2C are referenced without sufficient explanation of what they illustrate.
- *Response:* We updated the legend for Figures 2A-C to provide a more detailed description of what each figure represents (Lines 342-350).

7. **Sparse Comparison with AI-Estimated Prices:**

- *Comment:* The analysis places more emphasis on comparing crowdsourced and enumerator prices than on AI-estimated prices.
- *Response:* We updated the section to provide a balanced emphasis, focusing on how AI-estimated prices align with the other data sources. Kindly note that this analysis is stepwise, first focused on alignment between enumerator vs crowdsourced data, and later focused on crowdsourced vs AI price datasets (as explained in L543-547).

8. **Lack of Analysis on Temporal Trends:**

- *Comment:* The discussion on temporal trends is limited, lacking analysis of their effects on results.
- *Response:* We revised the text to clarify that our correlation analysis assessed the alignment of temporal trends between paired price datasets.

9. **Inconsistent Use of Statistical Metrics:**

- *Comment:* Various statistical metrics (e.g., R, r²) are used without clear rationale or interpretation.
- *Response:* We revised the manuscript to ensure consistency in the use of statistical metrics, aligning them with correlation analysis and citing relevant literature to justify our approach. We included full explanation regarding the theoretical underpinnings of the various statistical metrics in the Methods section and as supporting information. [L296-540]

10. **Omission of Limitations and Potential Biases:**

- *Comment:* The results section does not discuss potential limitations or biases in the data sources.
- *Response:* We have added a new paragraph in the discussion section to address potential biases and limitations in the data collection process (Lines 555-621).

11. **Inadequate Insight into Sub-Type Variability:**

- *Comment:* The implications of high variability in yellow maize prices are not fully explored.
- *Response:* To avoid confusion, we have minimized references to sub-type variability and focused more on general trends and analysis of the price for each commodity.

12. **Unclear Impact of Conversion Methods:**

- *Comment:* The conversion of prices to a per-kilogram basis is mentioned without discussion of its potential impact on comparisons.
- *Response:* We clarified the conversion process in the Methods section (Lines 275-279), emphasizing that conversion was applied across all datasets based on standard measures, but no effect was observed on trends because of the random distribution of quantities and aggregation of large volume of data into the time steps.

**Crowdsourced vs. AI-Estimated Commodity Price" section:**

Thank you for your thorough review of this section. In response, we consolidated redundant points and clarified the representation of results, especially regarding discrepancies between AI and crowdsourced data. We provided deeper analysis of specific divergences, expanded on the practical relevance of statistical metrics, and added context to explain observed price differences. We refined the explanation of time-lag analysis and data filtering methods for clarity. We also addressed the impact of temporal gaps in crowdsourced data and ensured better integration of Figures into the text. Additionally, we introduced a discussion of potential biases and limitations, and emphasized the practical implications of our findings. These revisions aim to improve the clarity and depth of this section.

1. **Redundancy in Explanation:**

- *Comment:* The section reiterates similar points about the relationship between crowdsourced and AI-estimated prices, leading to redundancy.
- *Response:* We revamped this section for improved readability and consolidated the points to avoid repetition.

2. **Inconsistent Analysis and Interpretation:**

- *Comment:* The analysis mentions a near-perfect correlation but does not sufficiently address month-to-month discrepancies and their impact on the overall assessment.
- *Response:* We revised the text to ensure accurate representation of the results and included additional clarification on how discrepancies were assessed (Lines 599-741). We also revamped the overall results and discussion sections for better clarity.

3. **Limited Discussion on Discrepancies:**

- *Comment:* The section notes specific divergences between AI and crowdsourced prices but does not explore their causes or implications.
- *Response:* We revised the text to highlight this non-spurious discrepancy as a potential issue for AI-based data (L649-656], provided further results on sensitivity and equivalence analysis to explore the price discrepancies (L742-796), and discussed in L856-863.

4. **Overemphasis on Statistical Metrics:**

- *Comment:* The focus on statistical metrics may obscure practical significance, particularly regarding the near-perfect correlation.
- *Response:* We added explanations to help readers interpret the practical relevance of these metrics (e.g. Lines 694-696; L707-707; L766-772).

5. **Lack of Contextual Explanation:**

- *Comment:* The section notes higher AI-estimated prices in certain contexts but lacks an explanation of contributing factors.
- *Response:* We revised the article to include more context on factors influencing price differences, such as market conditions and data collection methods (e.g. L41-842).

6. **Ambiguous Temporal Analysis:**

- *Comment:* The time-lag analysis shows a decline in correlation over time but lacks a clear explanation of the reasons and implications.
- *Response:* We reviewed and revised this paragraph to provide a more detailed discussion of the observed trend and its implications for the reliability of AI estimates (L657-664).

7. **Inadequate Detail on Data Filtering:**

- *Comment:* The section lacks detail on how filtering crowdsourced prices by market type was conducted.
- *Response:* To improve clarity, we revised the wording about data filtering to “querying” and provided more explanation in the methods (Lines 685-688).

8. **Insufficient Discussion on Data Gaps:**

- *Comment:* The impact of "inherent temporal gaps" in crowdsourced data is not sufficiently explored.
- *Response:* We clarified this point by adding a new section, revising the text to better contextualize these gaps for readers' understanding (Lines 943-954).

9. **Misalignment Between Figures and Text:**

- *Comment:* Figures 4 and 5 are not fully integrated into the text, making it hard to understand their significance.
- *Response:* We changed the positioning of the figures and revised the relevant sections (nof Figs 5 and 6b), adding more details to integrate them in context and improve readers’ comprehension (e.g. L657-682).

10. **Potential Biases and Limitations Not Addressed:**

- *Comment:* The analysis does not discuss potential biases or limitations in the data sources.
- *Response:* We added a new sub-section in the Discussion section to address potential biases and limitations (Lines 940-971); Also, prior to this section, we included additional sensitivity and equivalence test results including normality of price datasets to show non-bias (344-540).

11. **Failure to Address Practical Implications:**

- *Comment:* The section focuses on statistical relationships without discussing their practical implications for stakeholders.
- *Response:* We revised the text to highlight practical implications for market monitoring and price forecasting (e.g. L661-664; L757 – 772; L852-855).

12. **Lack of Clarity on Data Sources:**

- *Comment:* There is confusion about the specifics of the data sources used for AI estimation and crowdsourcing.
- *Response:* We revised the data description sections to provide clearer introductions and definitions of the datasets (Lines 161-267).

**Unraveling Relationships by Location, Commodity Type, and Market Segments section:**

Thank you for your thorough feedback on this section. In response, we have revised the text to provide a more nuanced presentation of results, added clarity on data disaggregation methods, and addressed variability in regional and market segment data. We improved the discussion on price differences across market segments and included information on outlier detection and its impact. The presentation of figures was refined for better integration with the text, and we emphasized practical implications alongside statistical findings. We also added context about market conditions, discussed data limitations, and provided insights on the replicability of the study. Additionally, we removed ambiguous terminology and compared our findings with relevant literature. These changes aim to provide a clearer and more balanced analysis of the relationships explored in this section.

1. **Overgeneralization of Results:**

- *Comment:* The section claims strong relationships between crowdsourced and AI-estimated prices without addressing potential inconsistencies at lower geographic levels or among different commodity sub-types.
- *Response:* We have revised the text to avoid overgeneralization and provided more nuanced details regarding the variability observed at different geographic levels, for each commodity sub-types (Lines 542-656).

2. **Lack of Detail on Methodology:**

- *Comment:* The process of disaggregating data by geographic level, commodity type, and market segment lacks detail.
- *Response:* We revised the text to specify that standard queries were used to select data according to these attributes, improving clarity (L687-690).

3. **Inadequate Discussion on Variability:**

- *Comment:* The section does not sufficiently discuss reasons for variability in prices across regions or market segments.
- *Response:* We revised the discussion section to address differences in variations across administrative boundaries and market segments (Lines 705 - 707).

4. **Superficial Analysis of Market Segments:**

- *Comment:* The section mentions price variations across market segments (Farmgate < Wholesale < Retail) without exploring the implications.
- *Response:* We provided further explanation of these price differences in the discussion section where it is more relevant.

5. **Failure to Address Outliers:**

- *Comment:* Strong correlations could be misleading if outliers or anomalies are not addressed.
- *Response:* We included additional text about outlier detection methods and their impact on results in both the methods section (L289-295) and the discussion section (L895-901).

6. **Inconsistent Presentation of Figures:**

- *Comment:* Figures are referenced without clear explanations of what they illustrate or how they support the analysis.
- *Response:* We revised the Figure titles throughout to integrate with text and provide clear explanations of their relevance to the overall paper focus (e.g. L625-636; L885-905).

7. **Overreliance on Statistical Metrics:**

- *Comment:* Emphasizing statistical significance may overshadow practical implications.
- *Response:* We revised the narrative to highlight the real-world applications of the findings, emphasizing their practical relevance in the discussion section [e.g. L932-938].

8. **Lack of Contextual Insight:**

- *Comment:* The section lacks information about the economic or market conditions of the regions analyzed.
- *Response:* We added context on the economic and market conditions in the Materials and Methods section to provide a better foundation for interpreting the results (Lines 129-140).

9. **Unexplored Data Limitations:**

- *Comment:* There is no discussion of potential limitations or biases in the data sources or analysis methods.
- *Response:* We added a paragraph discussing data limitations and potential biases to provide a balanced view of the study’s findings (L940-971).

10. **Insufficient Exploration of Discrepancies:**

- *Comment:* The section mentions differences in prices between crowdsourced and AI-estimated data without exploring their causes.
- *Response:* We added further discussion on these discrepancies in the discussion section to provide a more comprehensive understanding (L757 - 772).

11. **Potential Lack of Replicability:**

- *Comment:* The replicability of the findings across other datasets or regions is not addressed.
- *Response:* We included additional text to guide readers on the potential for replicability and future research directions (L932-938).

12. **Ambiguous Terminology:**

- *Comment:* Terms like "fragile context" and "co-variance" are not clearly defined.
- *Response:* We have removed these terms from the manuscript to avoid confusion.

13. **Inadequate Comparison to Previous Studies:**

- *Comment:* The section does not compare findings to previous studies or benchmarks.
- *Response:* We revised the manuscript to reference previous work and position this paper as foundational for future data validation efforts (L887-894).

**Discussion and Conclusion section:**

Thank you for your detailed feedback on the Discussion and Conclusion section. In response, we provided a balanced evaluation of the data's strengths and limitations by adding a new sub-section on potential caveats. We expanded our analysis of variability, considered external factors like market disruptions, and ensured that our references to figures were clear and relevant. We also provided a more nuanced view of the practical implications of our findings and addressed data gaps and challenges. The discussion now includes suggestions for future research, ethical considerations, and broader contextualization of the study’s relevance. These revisions aim to provide a more comprehensive and balanced discussion that accurately reflects the study's contributions and limitations.

1. **Lack of Critical Evaluation:**

- *Comment:* The discussion emphasizes positive outcomes without addressing limitations or potential weaknesses of the data.
- *Response:* We revised the discussion by introducing a new sub-section dedicated to potential limitations and caveats, providing a more balanced perspective (Lines L940-971).

2. **Overemphasis on Correlation:**

- *Comment:* The discussion highlights high correlation without exploring its practical implications or addressing the difference between correlation and causation.
- *Response:* We revised the manuscript to avoid any inference on causality, and clarified that the correlations were based on independent datasets (L149-152; L345-348). We included results from additional tests, transcending correlation, to establish coherence and relationships between the paired datasets.

3. **Insufficient Analysis of Variability:**

- *Comment:* The discussion does not sufficiently explore data variability or its impact on reliability.
- *Response:* We expanded the discussion to include analysis of spatial and temporal differences, providing deeper insight into the sensitivity and equivalence of the paired price datastes (856-873).

4. **Lack of Consideration for External Factors:**

- *Comment:* The impact of external factors like market disruptions or the COVID-19 pandemic is mentioned but not thoroughly analyzed.
- *Response:* We revised the text by presenting results from the sensitivity test which demonstrated that actual price process, not external factors, led to the findings of pairwise price correlations (L742-779; 856-863).

5. **Ambiguous References to Figures:**

- *Comment:* The text references figures without clearly explaining their relevance to the discussion.
- *Response:* We revised the manuscript to integrate the figures more effectively, providing clearer explanations of their relevance.

6. **Superficial Examination of Data Gaps:**

- *Comment:* The discussion of gaps in crowdsourced data is brief and lacks detail.
- *Response:* We expanded on this point, adding more detail about the effects of data gaps on analysis continuity and validity (L944-955).

7. **Overstated Claims about Crowdsourcing and AI:**

- *Comment:* The discussion makes strong claims about the reliability of crowdsourcing and AI without addressing challenges.
- *Response:* We expanded the discussion of limitations, providing a more nuanced view of the challenges associated with crowdsourced and AI-generated data (Lines 920-932).

8. **Unclear Practical Implications:**

- *Comment:* The practical implications of the findings for real-world applications are not fully developed.
- *Response:* We revised the text to provide more specific examples of how the results can be applied in practice, especially in low-resource settings (L933-939).

9. **Overreliance on Statistical Significance:**

- *Comment:* The focus on statistical significance may overshadow practical significance.
- *Response:* We revised the discussion to emphasize the real-world implications for stakeholders, balancing statistical and practical perspectives (L930-932).

10. **Inadequate Exploration of Data Source Integration:**

- *Comment:* The discussion mentions data integration potential without providing a practical framework for achieving it.
- *Response:* We added a conceptual proposal for integrating crowdsourced and AI-generated data sources (L975-985; L991 - 996).

11. **Failure to Address Methodological Limitations:**

- *Comment:* The discussion does not sufficiently address potential methodological limitations, such as data collection biases.
- *Response:* This point is addressed in the expanded Methods section and discussion of limitations, ensuring a more comprehensive understanding of the study’s constraints (L345-373; L991-994).

12. **Limited Insight into Data Quality:**

- *Comment:* There is insufficient discussion on the quality of crowdsourced data compared to traditional enumerator data.
- *Response:* We revised the text to include a discussion on data quality assessment methods and potential issues with data accuracy (L901-919).

13. **Lack of Future Research Directions:**

- *Comment:* The section does not offer recommendations for future research or improvements.
- *Response:* We have added suggestions for future research directions, including advancements in data collection methods and AI algorithms (L933-939; L993-996).

14. **Overlooked Ethical Considerations:**
1. *Comment:* The discussion does not address ethical considerations related to crowdsourcing, such as data privacy.
2. *Response:* We included a note on potential ethical considerations to ensure responsible use of innovative data collection methods (L991-998).
15. **Limited Contextualization:**

- *Comment:* The analysis does not consider how findings might differ in other regions or countries.
- *Response:* We revised the text to discuss the potential applicability of the findings to regions beyond northern Nigeria, providing broader contextualization (L998-1000).

**References:**

Thank you for your feedback on the References section. In response, we refined our references to ensure greater focus, removing outdated or less relevant sources and incorporating a broader range of data perspectives. We clarified the presentation of data sources and included more recent studies where appropriate. We also addressed concerns about potential biases by ensuring transparency in data origins and highlighting the independent nature of various datasets. Additionally, we added a critical discussion of data limitations and potential biases to provide a more balanced view. These revisions aim to enhance the overall relevance, rigor, and clarity of the referenced materials.

1. **Lack of Cohesion and Structure:**

- *Comment:* The references span a broad range of topics, which may indicate a lack of focus or cohesion in the literature review.
- *Response:* We revised the references to exclude less relevant sources, ensuring alignment with the revisions made throughout the paper for improved focus and relevance.

2. **Outdated or Less Relevant Sources:**

- *Comment:* Some sources may be outdated, given the rapid developments in the field of food security.
- *Response:* The references were reviewed to include a more balanced mix of recent and relevant publications that directly support the study.

3. **Overemphasis on Certain Types of Data:**

- *Comment:* There is a heavy reliance on World Bank reports and crowdsourced data, which may limit the diversity of perspectives.
- *Response:* We revised the references to include a broader representation of perspectives, including academic papers. Some references have been removed as well.

4. **Potential Bias in Data Sources:**

- *Comment:* References from organizations like the World Bank or IMF might introduce bias due to their economic perspectives.
- *Response:* As noted above, we have cited openly accessible documentation on the World Bank’s AI-price imputation to ensure transparency. The independent nature of the crowdsourced data from the EC-JRC project is also highlighted.

5. **Technical and Niche Focus:**

- *Comment:* Some references on advanced methodologies might be too technical for general readers.
- *Response:* We carefully reviewed these references and believe they are necessary for guiding readers’ understanding of the technical aspects of the study, supporting the main text’s rigor. We also provide less technical explanations in the paper itself for general readers.

6. **Inconsistencies in Data and Analysis:**

- *Comment:* Disparities between crowdsourced data and official statistics might lead to conflicting conclusions.
- *Response:* We refined the presentation of data sources for clarity and consistency, ensuring better flow and comprehension for readers (Lines 155-225).

7. **Limited Geographic Focus:**

- *Comment:* Many references focus on specific regions, like Nigeria and Africa, which might limit the applicability of findings to other contexts.
- *Response:* We revised the manuscript to acknowledge the current geographic focus (Lines 655-658). The need for alternative price data is especially critical in low- and middle-income countries, making sub-Saharan Africa a focus area for this study.

8. **Lack of Critical Analysis:**

- *Comment:* The references primarily present data without a critical analysis of their limitations or potential biases.
- *Response:* We revised the manuscript to demonstrate a more rigorous approach throughout, adding a new paragraph that discusses potential data biases, limitations, and caveats (Lines 941-972).

**Supporting information text:**

Thank you for your feedback on the Supporting Information Text section. In response, we revised figure captions for greater clarity, defined terms like "intraday submissions" and "log-transformed counts," and ensured better explanations of data handling and availability. We provided direct URLs for data access, improved the organization of data sources in the methods section, and resolved formatting inconsistencies for a more professional presentation. Additionally, we expanded the methodology descriptions and ensured figures are more clearly integrated into the overall analysis. These changes aim to enhance the clarity, accessibility, and professionalism of the supporting information provided.

1. **Lack of Clarity in Figure Descriptions:**

- *S1 Fig:* The description is vague, especially regarding "cohesion of price signal" and how it is measured.
- *Response:* The figure caption has been revised for clarity, providing a more detailed explanation was provided and the term “cohesion” was excluded.
- *S2 Fig:* The term "fragile context" is used without explanation, and the discrepancy in the number of geolocated market points needs more context.
- *Response:* We removed the term “fragile context” from the caption and added additional details to clarify the reasons behind the discrepancy in geolocated market points.
- *S3 Fig:* The relationship between crowdsourced and AI-estimated prices is not explained in detail, particularly in terms of disaggregation by state and commodity subtype.
- *Response:* The figure has been repositioned and the caption updated to include a more detailed explanation of the disaggregation process and its impact on the analysis.

2. **Inconsistencies and Unexplained Terms:**

- *S1 Fig:* The reference to "intraday datapoints" and their averaging into different time intervals is not fully explained.
- *Response:* We have updated the caption for all figures to define “intraday submissions” and provided a more detailed explanation of the averaging process in the main text for clarity.
- *S4 Fig:* The term "log-transformed counts" is not explained.
- *Response:* The figure has been repositioned within text. It is now Fig 6b(ii). The caption has been revised to include an explanation of the log transformation and its relevance to the data interpretation.

3. **Potential Data Access Issues:**

- *Comment:* The text mentions that datasets are available through repositories but lacks direct links, which could be frustrating for readers.
- *Response:* We revised the data availability section to include direct URLs for easier access, including doi. Also, we checked and verified that the repositories are fully open access.

4. **Inadequate Description of Data Availability:**

- *Comment:* The description of data availability is dense and mixes data sources, which could lead to confusion.
- *Response:* We revised the methods section to include sub-headings for each dataset, making the presentation of data sources clearer (161-267).

5. **Missing Details on Data Handling:**

- *Comment:* The text mentions intraday datasets but does not explain their content or how they differ from daily averages.
- *Response:* We revised the methods section to provide more detailed explanations of the data processing, including how intraday submissions were averaged into daily, weekly, and monthly prices.

6. **Redundancy and Formatting Issues:**

- *Comment:* There are inconsistencies in formatting, such as repeated "Formatted: Font" notes, which detract from professionalism.
- *Response:* We have resolved these formatting issues to ensure a more professional and polished presentation.

7. **Inadequate Explanation of Methodology:**

- *Comment:* The description of how correlation analysis was performed is brief, making it difficult to assess the robustness of the analysis.
- *Response:* We have revised the methods section for enhanced clarity, providing more detail on the statistical methods used for correlation analysis.

8. **Unclear Presentation of Figures and Data:**

- *Comment:* The text references figures without explaining how they relate to each other or support the study’s conclusions.
- *Response:* We revised the manuscript to include more detailed captions for each figure and provided explanations that clarify their significance and interconnections.

**The main areas where the manuscript requires further revision:**

Thank you for your constructive feedback on the key areas needing revision. We have addressed each point thoroughly, enhancing the clarity and detail of figure descriptions, providing direct data access links, and improving the explanation of statistical methods. Our discussion now includes a deeper exploration of trends and outliers, as suggested, and we have refined the presentation of figures for better integration and understanding. Formatting issues have been resolved, ensuring consistency and professionalism throughout the manuscript. These revisions aim to provide a more accurate, accessible, and well-structured manuscript that meets the reviewers’ expectations.

1. **Clarity and Detail in Supporting Information:**

- *Comment:* The descriptions of figures (S1-S4) lack detail, including explanations of terms like "cohesion of price signal" and "log-transformed counts."
- *Response:* We have revised the captions for all supporting figures to provide clearer and more detailed explanations of these terms. The rationale behind the methods used is now better articulated, ensuring a comprehensive understanding of the data presented.

2. **Consistency and Completeness of Data Access Information:**

- *Comment:* The information on data availability is incomplete and confusing, lacking direct links to data repositories.
- *Response:* We have revised the manuscript to include direct and functional dois for all data repositories, ensuring that data availability descriptions are clear and accurate. This improvement facilitates easier access and verification of the datasets by readers.

3. **Explanation of Statistical Methods:**

- *Comment:* Insufficient detail is provided regarding the statistical methods used for correlation analysis and time-lagged relationships.
- *Response:* The Methods section has been revised to offer a more detailed explanation of the statistical techniques, including how assumptions were handled and the approach to time-lagged analysis. This ensures greater transparency and understanding of the robustness of our analysis.

4. **Addressing Review Comments Thoroughly:**

- *Comment:* Some reviewer comments, particularly regarding trends and outliers, have not been fully addressed.
- *Response:* We revisited the reviewers' comments and made further revisions to expand our discussion of unexpected trends and outliers, providing a more balanced interpretation. This ensures that all aspects of the feedback are comprehensively incorporated.

5. **Improving Figure and Data Presentation:**

- *Comment:* Figures and related data need clearer presentation and better integration with the text.
- *Response:* We reviewed and revised the figure captions to enhance clarity. Figures are now clearly referenced in the text, and their captions provide sufficient context to understand the data without ambiguity.

6. **Formatting and Professionalism:**

- *Comment:* The manuscript has formatting inconsistencies that detract from its professionalism.
- *Response:* All formatting issues have been addressed. The manuscript has been reviewed for consistency throughout, and any extraneous formatting notes have been removed to ensure a professional presentation.
