# Supplementary material for: AI-imputed and crowdsourced price data show strong agreement with traditional price surveys in data-scarce environments
Source: PLoS One. 2025 Apr 8;20(4):e0320720. doi: 10.1371/journal.pone.0320720 (PMC11978078; doi:10.1371/journal.pone.0320720)
Supplement: Peer review history

# Author comment: Author response to Decision Letter 0

**DOI:** 10.1371/journal.pone.0320720.r001

4 Jun 2024

# Peer review: Decision Letter 0

**DOI:** 10.1371/journal.pone.0320720.r002

**Authors:** Pradeep Paraman

1 Jul 2024

PONE-D-24-22683High-frequency food prices from Artificial Intelligence and crowdsourcing approach validated with groundtruth data in a fragile contextPLOS ONE

Dear Dr. Adewopo,

Thank you for submitting your manuscript to PLOS ONE. After careful consideration, we feel that it has merit but does not fully meet PLOS ONE’s publication criteria as it currently stands. Therefore, we invite you to submit a revised version of the manuscript that addresses the points raised during the review process. Please submit your revised manuscript by Aug 15 2024 11:59PM. If you will need more time than this to complete your revisions, please reply to this message or contact the journal office at plosone@plos.org . When you're ready to submit your revision, log on to https://www.editorialmanager.com/pone/ and select the 'Submissions Needing Revision' folder to locate your manuscript file.

Please include the following items when submitting your revised manuscript:

- A rebuttal letter that responds to each point raised by the academic editor and reviewer(s). You should upload this letter as a separate file labeled 'Response to Reviewers'.
- A marked-up copy of your manuscript that highlights changes made to the original version. You should upload this as a separate file labeled 'Revised Manuscript with Track Changes'.
- An unmarked version of your revised paper without tracked changes. You should upload this as a separate file labeled 'Manuscript'.

If you would like to make changes to your financial disclosure, please include your updated statement in your cover letter. Guidelines for resubmitting your figure files are available below the reviewer comments at the end of this letter.

If applicable, we recommend that you deposit your laboratory protocols in protocols.io to enhance the reproducibility of your results. Protocols.io assigns your protocol its own identifier (DOI) so that it can be cited independently in the future. For instructions see: https://journals.plos.org/plosone/s/submission-guidelines#loc-laboratory-protocols . Additionally, PLOS ONE offers an option for publishing peer-reviewed Lab Protocol articles, which describe protocols hosted on protocols.io. Read more information on sharing protocols at https://plos.org/protocols?utm_medium=editorial-email&utm_source=authorletters&utm_campaign=protocols .

We look forward to receiving your revised manuscript.

Kind regards,

Pradeep Paraman

Academic Editor

PLOS ONE

Journal Requirements:

When submitting your revision, we need you to address these additional requirements.

1. Please ensure that your manuscript meets PLOS ONE's style requirements, including those for file naming. The PLOS ONE style templates can be found at 

https://journals.plos.org/plosone/s/file?id=wjVg/PLOSOne_formatting_sample_main_body.pdf and 

https://journals.plos.org/plosone/s/file?id=ba62/PLOSOne_formatting_sample_title_authors_affiliations.pdf

2. Please note that PLOS ONE has specific guidelines on code sharing for submissions in which author-generated code underpins the findings in the manuscript. In these cases, we expect all author-generated code to be made available without restrictions upon publication of the work. Please review our guidelines at https://journals.plos.org/plosone/s/materials-and-software-sharing#loc-sharing-code and ensure that your code is shared in a way that follows best practice and facilitates reproducibility and reuse.

3. Please note that funding information should not appear in any section or other areas of your manuscript. We will only publish funding information present in the Funding Statement section of the online submission form. Please remove any funding-related text from the manuscript.

4. Thank you for stating the following financial disclosure: 

   "Funding by the Federal Ministry for Economic Cooperation and Development (BMZ, Germany) as part of the World Bank’s Food Systems 2030 (FS2030) Multi-Donor Trust Fund program (grants TF073570 and TF0C0728)

European Commission Joint Research Center (EC-JRC)

Agropolis Foundation for the research mobility under Louis Malassis International Scientific Prize 2019"

Please state what role the funders took in the study.  If the funders had no role, please state: "The funders had no role in study design, data collection and analysis, decision to publish, or preparation of the manuscript." 

If this statement is not correct you must amend it as needed. 

Please include this amended Role of Funder statement in your cover letter; we will change the online submission form on your behalf.

5. When completing the data availability statement of the submission form, you indicated that you will make your data available on acceptance. We strongly recommend all authors decide on a data sharing plan before acceptance, as the process can be lengthy and hold up publication timelines. Please note that, though access restrictions are acceptable now, your entire data will need to be made freely accessible if your manuscript is accepted for publication. This policy applies to all data except where public deposition would breach compliance with the protocol approved by your research ethics board. If you are unable to adhere to our open data policy, please kindly revise your statement to explain your reasoning and we will seek the editor's input on an exemption. Please be assured that, once you have provided your new statement, the assessment of your exemption will not hold up the peer review process.

6. We note that you have included the phrase “data not shown” in your manuscript. Unfortunately, this does not meet our data sharing requirements. PLOS does not permit references to inaccessible data. We require that authors provide all relevant data within the paper, Supporting Information files, or in an acceptable, public repository. Please add a citation to support this phrase or upload the data that corresponds with these findings to a stable repository (such as Figshare or Dryad) and provide and URLs, DOIs, or accession numbers that may be used to access these data. Or, if the data are not a core part of the research being presented in your study, we ask that you remove the phrase that refers to these data.

7. We note that Figure 1 in your submission contain map/satellite images which may be copyrighted. All PLOS content is published under the Creative Commons Attribution License (CC BY 4.0), which means that the manuscript, images, and Supporting Information files will be freely available online, and any third party is permitted to access, download, copy, distribute, and use these materials in any way, even commercially, with proper attribution. For these reasons, we cannot publish previously copyrighted maps or satellite images created using proprietary data, such as Google software (Google Maps, Street View, and Earth). For more information, see our copyright guidelines: http://journals.plos.org/plosone/s/licenses-and-copyright.

We require you to either (a) present written permission from the copyright holder to publish these figures specifically under the CC BY 4.0 license, or (b) remove the figures from your submission:

a. You may seek permission from the original copyright holder of Figure(s) [#] to publish the content specifically under the CC BY 4.0 license.  

We recommend that you contact the original copyright holder with the Content Permission Form (http://journals.plos.org/plosone/s/file?id=7c09/content-permission-form.pdf) and the following text:

“I request permission for the open-access journal PLOS ONE to publish XXX under the Creative Commons Attribution License (CCAL) CC BY 4.0 (http://creativecommons.org/licenses/by/4.0/). Please be aware that this license allows unrestricted use and distribution, even commercially, by third parties. Please reply and provide explicit written permission to publish XXX under a CC BY license and complete the attached form.”

Please upload the completed Content Permission Form or other proof of granted permissions as an "Other" file with your submission.

In the figure caption of the copyrighted figure, please include the following text: “Reprinted from [ref] under a CC BY license, with permission from [name of publisher], original copyright [original copyright year].”

b. If you are unable to obtain permission from the original copyright holder to publish these figures under the CC BY 4.0 license or if the copyright holder’s requirements are incompatible with the CC BY 4.0 license, please either i) remove the figure or ii) supply a replacement figure that complies with the CC BY 4.0 license. Please check copyright information on all replacement figures and update the figure caption with source information. If applicable, please specify in the figure caption text when a figure is similar but not identical to the original image and is therefore for illustrative purposes only.

The following resources for replacing copyrighted map figures may be helpful:

USGS National Map Viewer (public domain): http://viewer.nationalmap.gov/viewer/

The Gateway to Astronaut Photography of Earth (public domain): http://eol.jsc.nasa.gov/sseop/clickmap/

Maps at the CIA (public domain): https://www.cia.gov/library/publications/the-world-factbook/index.html and https://www.cia.gov/library/publications/cia-maps-publications/index.html

NASA Earth Observatory (public domain): http://earthobservatory.nasa.gov/

Landsat: http://landsat.visibleearth.nasa.gov/

USGS EROS (Earth Resources Observatory and Science (EROS) Center) (public domain): http://eros.usgs.gov/#

Natural Earth (public domain): http://www.naturalearthdata.com/

8. Please include captions for your Supporting Information files at the end of your manuscript, and update any in-text citations to match accordingly. Please see our Supporting Information guidelines for more information: http://journals.plos.org/plosone/s/supporting-information. 

9. Please review your reference list to ensure that it is complete and correct. If you have cited papers that have been retracted, please include the rationale for doing so in the manuscript text, or remove these references and replace them with relevant current references. Any changes to the reference list should be mentioned in the rebuttal letter that accompanies your revised manuscript. If you need to cite a retracted article, indicate the article’s retracted status in the References list and also include a citation and full reference for the retraction notice.

[Note: HTML markup is below. Please do not edit.]

Reviewers' comments:

Reviewer's Responses to Questions

**Comments to the Author**

1. Is the manuscript technically sound, and do the data support the conclusions?

The manuscript must describe a technically sound piece of scientific research with data that supports the conclusions. Experiments must have been conducted rigorously, with appropriate controls, replication, and sample sizes. The conclusions must be drawn appropriately based on the data presented. 

Reviewer #1: Yes

Reviewer #2: Yes

**********

2. Has the statistical analysis been performed appropriately and rigorously? 

Reviewer #1: Yes

Reviewer #2: No

**********

3. Have the authors made all data underlying the findings in their manuscript fully available?

The PLOS Data policy requires authors to make all data underlying the findings described in their manuscript fully available without restriction, with rare exception (please refer to the Data Availability Statement in the manuscript PDF file). The data should be provided as part of the manuscript or its supporting information, or deposited to a public repository. For example, in addition to summary statistics, the data points behind means, medians and variance measures should be available. If there are restrictions on publicly sharing data—e.g. participant privacy or use of data from a third party—those must be specified.

Reviewer #1: Yes

Reviewer #2: Yes

**********

4. Is the manuscript presented in an intelligible fashion and written in standard English?

PLOS ONE does not copyedit accepted manuscripts, so the language in submitted articles must be clear, correct, and unambiguous. Any typographical or grammatical errors should be corrected at revision, so please note any specific errors here.

Reviewer #1: Yes

Reviewer #2: Yes

**********

5. Review Comments to the Author

Please use the space provided to explain your answers to the questions above. You may also include additional comments for the author, including concerns about dual publication, research ethics, or publication ethics. (Please upload your review as an attachment if it exceeds 20,000 characters)

Reviewer #1: Reviewer Comment

Title: Validation of AI and Crowdsourced Food Price Data for Real-Time Monitoring in Fragile Contexts

Specific Comments

1. Abstract

The abstract is well-written and presents important findings.

• Explicitly state the research gap the study addresses. For example, mention the limitations of current price monitoring methods and the specific contributions of this study

• Include the statistical methods used for correlation analysis, such as the Pearson correlation coefficient.

2. Introduction

• Lines 64-87: The challenges section is comprehensive but could be summarized to focus on the key points.

• Lines 88-105: The introduction to data crowdsourcing and AI is detailed. Streamline this section to focus on the relevance of these methods to the study's objectives.

• Lines 139-150: The study's objective should be clearly highlighted, summarizing the validation effort's key goals and significance.

3. Methods

• Lines 155-177: Consider breaking the paragraph into smaller sections to improve readability. For example, separate discussions on socio-economic context, agricultural production, and market dynamics into distinct paragraphs.

• Lines 177-182: The map description is clear. Ensure it is referenced appropriately within the text to guide readers' understanding of the study area's scope.

4. Results

• Provide concise summaries at the beginning of each subsection (e.g., Ground truth vs. Crowdsourced Prices, crowdsourced vs. AI-Estimated Prices) to orient readers to the focus and findings of that particular analysis.

• Emphasize the practical implications of findings, such as how the coherence between datasets (e.g., crowdsourced vs. enumerator) at different time intervals impacts the reliability of high-frequency surveillance in food price monitoring.

• Consider discussing outliers or unexpected trends observed in the data, as this can provide insights into the limitations or strengths of each data source.

5. Discussion

• Clearly link each discussion point directly to the study objectives to maintain focus.

• Describe trends and patterns highlighted by figures and relate them back to broader discussion points on data reliability and applicability.

Overall Recommendation

The study is valuable, and warrants publication after the above concerns are addressed.

Reviewer #2: The paper can be accepted subject to the following modification:

1. The data interpretation should be accompanied with some suitable visualisations. This should be considered within the section Materials and Methods. It would be interesting to see how the variables are distributed over different geographic regions, market areas etc.

2. Assumptions regarding the data prior to correlation analysis should be explained clearly. Time lagged relationship between the crowd sourced data and the AI generated data should also be identified using cross correlation techniques. The visualisations shown as per the comment number 1 should also be helpful in indicating the time lagged properties of the data.

**********

6. PLOS authors have the option to publish the peer review history of their article (what does this mean? ). If published, this will include your full peer review and any attached files.

If you choose “no”, your identity will remain anonymous but your review may still be made public.

**Do you want your identity to be public for this peer review?** For information about this choice, including consent withdrawal, please see our Privacy Policy .

Reviewer #1: No

Reviewer #2: No

**********

[NOTE: If reviewer comments were submitted as an attachment file, they will be attached to this email and accessible via the submission site. Please log into your account, locate the manuscript record, and check for the action link "View Attachments". If this link does not appear, there are no attachment files.]

While revising your submission, please upload your figure files to the Preflight Analysis and Conversion Engine (PACE) digital diagnostic tool, https://pacev2.apexcovantage.com/ . PACE helps ensure that figures meet PLOS requirements. To use PACE, you must first register as a user. Registration is free. Then, login and navigate to the UPLOAD tab, where you will find detailed instructions on how to use the tool. If you encounter any issues or have any questions when using PACE, please email PLOS at figures@plos.org . Please note that Supporting Information files do not need this step.

# Author comment: Author response to Decision Letter 1

**DOI:** 10.1371/journal.pone.0320720.r003

9 Aug 2024

Response Letter to Editor and Reviewer Notes

**We thank the reviewers and editor for the extra diligence in reviewing this manuscript. The comments, suggestions, and notes have been very useful to enhance the content and clarity of the manuscript. Responses to each note are provided in texts below.

1. Please ensure that your manuscript meets PLOS ONE's style requirements, including those for file naming. The PLOS ONE style templates can be found at

https://journals.plos.org/plosone/s/file?id=wjVg/PLOSOne_formatting_sample_main_body.pdf and

https://journals.plos.org/plosone/s/file?id=ba62/PLOSOne_formatting_sample_title_authors_affiliations.pdf

Response: All formatting requirements have been reviewed and addressed throughout the manuscript

2. Please note that PLOS ONE has specific guidelines on code sharing for submissions in which author-generated code underpins the findings in the manuscript. In these cases, we expect all author-generated code to be made available without restrictions upon publication of the work. Please review our guidelines at https://journals.plos.org/plosone/s/materials-and-software-sharing#loc-sharing-code and ensure that your code is shared in a way that follows best practice and facilitates reproducibility and reuse.

Response:The guidelines for code sharing were duly followed, with DOI and associated notes published in the reproducibility package. We published the reproducibility package through WorldBank’s rigorous peer-review process, which includes exhaustive verification of the code and final independent report on the reproducibility of the outputs. The reproducibility package can be accessed here . Also, we have created a new version or the code and posted in GitHub, updated with annotation that corresponds to the figure labeling within the paper. The url is https://github.com/PJNation/FoodPriceAnalytics

3. Please note that funding information should not appear in any section or other areas of your manuscript. We will only publish funding information present in the Funding Statement section of the online submission form. Please remove any funding-related text from the manuscript.

Response:Done; Funding and other extraneous information has been removed

4. Thank you for stating the following financial disclosure:

"Funding by the Federal Ministry for Economic Cooperation and Development (BMZ, Germany) as part of the World Bank’s Food Systems 2030 (FS2030) Multi-Donor Trust Fund program (grants TF073570 and TF0C0728)

European Commission Joint Research Center (EC-JRC) Agropolis Foundation for the research mobility under Louis Malassis International Scientific Prize 2019"

Please state what role the funders took in the study. If the funders had no role, please state: "The funders had no role in study design, data collection and analysis, decision to publish, or preparation of the manuscript."

If this statement is not correct you must amend it as needed.

Please include this amended Role of Funder statement in your cover letter; we will change the online submission form on your behalf.

Response:The role of funders is now indicated in the cover letter for your reference and update in the online submission

5. When completing the data availability statement of the submission form, you indicated that you will make your data available on acceptance. We strongly recommend all authors decide on a data sharing plan before acceptance, as the process can be lengthy and hold up publication timelines. Please note that, though access restrictions are acceptable now, your entire data will need to be made freely accessible if your manuscript is accepted for publication. This policy applies to all data except where public deposition would breach compliance with the protocol approved by your research ethics board. If you are unable to adhere to our open data policy, please kindly revise your statement to explain your reasoning and we will seek the editor's input on an exemption. Please be assured that, once you have provided your new statement, the assessment of your exemption will not hold up the peer review process.

Response:As stated in the manuscript, the entire datasets are already published in public access repositories and the extracted data are included in the published reproducibility package. However, we have created a new version of the code and posted in GitHub, with annotation that corresponds to the figure labeling within the paper. The public access url is https://github.com/PJNation/FoodPriceAnalytics

6. We note that you have included the phrase “data not shown” in your manuscript. Unfortunately, this does not meet our data sharing requirements. PLOS does not permit references to inaccessible data. We require that authors provide all relevant data within the paper, Supporting Information files, or in an acceptable, public repository. Please add a citation to support this phrase or upload the data that corresponds with these findings to a stable repository (such as Figshare or Dryad) and provide and URLs, DOIs, or accession numbers that may be used to access these data. Or, if the data are not a core part of the research being presented in your study, we ask that you remove the phrase that refers to these data.

Response:This phrase was inadvertently included in the paper and has been excluded because the full AI and crowdsourced data are included in the data repository. The average values are calculated from the respectively datasets in the open access reproducibility package.

7. We note that Figure 1 in your submission contain map/satellite images which may be copyrighted. All PLOS content is published under the Creative Commons Attribution License (CC BY 4.0), which means that the manuscript, images, and Supporting Information files will be freely available online, and any third party is permitted to access, download, copy, distribute, and use these materials in any way, even commercially, with proper attribution. For these reasons, we cannot publish previously copyrighted maps or satellite images created using proprietary data, such as Google software (Google Maps, Street View, and Earth). For more information, see our copyright guidelines: http://journals.plos.org/plosone/s/licenses-and-copyright.

We require you to either (a) present written permission from the copyright holder to publish these figures specifically under the CC BY 4.0 license, or (b) remove the figures from your submission:

a. You may seek permission from the original copyright holder of Figure(s) [#] to publish the content specifically under the CC BY 4.0 license.

We recommend that you contact the original copyright holder with the Content Permission Form (http://journals.plos.org/plosone/s/file?id=7c09/content-permission-form.pdf) and the following text:

“I request permission for the open-access journal PLOS ONE to publish XXX under the Creative Commons Attribution License (CCAL) CC BY 4.0 (http://creativecommons.org/licenses/by/4.0/). Please be aware that this license allows unrestricted use and distribution, even commercially, by third parties. Please reply and provide explicit written permission to publish XXX under a CC BY license and complete the attached form.”

Please upload the completed Content Permission Form or other proof of granted permissions as an "Other" file with your submission.

In the figure caption of the copyrighted figure, please include the following text: “Reprinted from [ref] under a CC BY license, with permission from [name of publisher], original copyright [original copyright year].”

b. If you are unable to obtain permission from the original copyright holder to publish these figures under the CC BY 4.0 license or if the copyright holder’s requirements are incompatible with the CC BY 4.0 license, please either i) remove the figure or ii) supply a replacement figure that complies with the CC BY 4.0 license. Please check copyright information on all replacement figures and update the figure caption with source information. If applicable, please specify in the figure caption text when a figure is similar but not identical to the original image and is therefore for illustrative purposes only.

The following resources for replacing copyrighted map figures may be helpful:

USGS National Map Viewer (public domain): http://viewer.nationalmap.gov/viewer/

The Gateway to Astronaut Photography of Earth (public domain): http://eol.jsc.nasa.gov/sseop/clickmap/

Maps at the CIA (public domain): https://www.cia.gov/library/publications/the-world-factbook/index.html and https://www.cia.gov/library/publications/cia-maps-publications/index.html

NASA Earth Observatory (public domain): http://earthobservatory.nasa.gov/

Landsat: http://landsat.visibleearth.nasa.gov/

USGS EROS (Earth Resources Observatory and Science (EROS) Center) (public domain): http://eros.usgs.gov/#

Natural Earth (public domain): http://www.naturalearthdata.com/

Response:We created the boundary maps from the scratch with open-access country (Nigeria) and Africa-continent boundaries which were obtained from www.gadm.org. We checked and confirmed that Figure 1 (Map of study area) does not contain any additional satellite imagery or copyrighted materials. The source allows for use and publishing of maps and shapefile data in PloSOne as stated in this license permission clause - https://gadm.org/license.html. Our georeferenced price datapoints were used to create the location point shapefile layers that were included in the first map (Fig 1). The hash-line shading in the map is mere symbology of the focal states and no other spatial layers or maps are embedded. The grey background was a mere color-fill, not a basemap, and it does not convey any information. Additional information regarding the source of the shapefile has been added to the Figure caption.

8. Please include captions for your Supporting Information files at the end of your manuscript, and update any in-text citations to match accordingly. Please see our Supporting Information guidelines for more information: http://journals.plos.org/plosone/s/supporting-information.

Response:The Supporting information section has been duly updated as recommended, following PloSOne guideline.

9. Please review your reference list to ensure that it is complete and correct. If you have cited papers that have been retracted, please include the rationale for doing so in the manuscript text, or remove these references and replace them with relevant current references. Any changes to the reference list should be mentioned in the rebuttal letter that accompanies your revised manuscript. If you need to cite a retracted article, indicate the article’s retracted status in the References list and also include a citation and full reference for the retraction notice.

Response:Reference list has been reviewed for completeness; There is no instance of retracted article so no changes were made on the list to warrant additional notes

Reviewer #1: Reviewer Comment

Title: Validation of AI and Crowdsourced Food Price Data for Real-Time Monitoring in Fragile Contexts

Specific Comments

1. Abstract

The abstract is well-written and presents important findings.

• Explicitly state the research gap the study addresses. For example, mention the limitations of current price monitoring methods and the specific contributions of this study

Response:Thank you for the suggestion. The abstract has been revised to further bring the research problem to fore, within the word count limit [L32-33] and [L36-41]

• Include the statistical methods used for correlation analysis, such as the Pearson correlation coefficient.

The statistical method is now included in the abstract, as recommended

2. Introduction

Response:• Lines 64-87: The challenges section is comprehensive but could be summarized to focus on the key points.

We deleted [L67-69] and [L79-82] to shorten the paragraph, and revised the paragraph slightly to sharpen the focus

• Lines 88-105: The introduction to data crowdsourcing and AI is detailed. Streamline this section to focus on the relevance of these methods to the study's objectives.

Response:We intended to provide relevant contexts that can guide the understanding of the audience, however, following the reviewers’ comment, we have deleted [L99-101]. This narrative provides a prelude to justify data innovation for food system

• Lines 139-150: The study's objective should be clearly highlighted, summarizing the validation effort's key goals and significance.

Response:Thank you. This section has been revised to clearly call out the objective. Additional texts were added to highlight the significance of the analysis as well.

3. Methods

• Lines 155-177: Consider breaking the paragraph into smaller sections to improve readability. For example, separate discussions on socio-economic context, agricultural production, and market dynamics

Response: This is a helpful suggestion. We have split the context into 2 paragraphs for better readability; The first part addresses the socio-economic context while the second part addresses the Agricultural production/market context.

We presume that this is

• Lines 177-182: The map description is clear. Ensure it is referenced appropriately within the text to guide readers' understanding of the study area's scope.

Response:Thank you for confirming that the map description is clear. We checked and confirmed that it is reference appropriately within the text as well.

4. Results

• Provide concise summaries at the beginning of each subsection (e.g., Ground truth vs. Crowdsourced Prices, crowdsourced vs. AI-Estimated Prices) to orient readers to the focus and findings of that particular analysis.

Response:Thank you. For clarity, we have revised the beginning of the sub-sections to point readers to the summary note before delving into details.

• Emphasize the practical implications of findings, such as how the coherence between datasets (e.g., crowdsourced vs. enumerator) at different time intervals impacts the reliability of high-frequency surveillance in food price monitoring.

Response:This is definitely an important note which rightly fits under the discussion and conclusion section, as already presented there. To avoid redundancy, we retain the rendition of the results with minimal modification.

• Consider discussing outliers or unexpected trends observed in the data, as this can provide insights into the limitations or strengths of each data source.

Response:Indeed! We appreciate this valuable suggestion and we have added texts into the discussion section regarding outliers and their connotation for future research interests

5. Discussion

• Clearly link each discussion point directly to the study objectives to maintain focus.

Response:This is a helpful note. We have revised the discussion section to improve the flow and focus for the audience.

• Describe trends and patterns highlighted by figures and relate them back to broader discussion points on data reliability and applicability.

Response:Per note above, we revised and updated texts to bring focus to data pattern.

Overall Recommendation

The study is valuable, and warrants publication after the above concerns are addressed.

Reviewer #2: The paper can be accepted subject to the following modification:

1. The data interpretation should be accompanied with some suitable visualisations. This should be considered within the section Materials and Methods. It would be interesting to see how the variables are distributed over different geographic regions, market areas etc.

Response:We thank the reviewer for this comment. We conducted extra analysis of time-lagged correlations between AI-generated and crowdsourced prices, and included 2 new maps (Fig 6b and Fig 6c), a new chart (Fig 5), and additional texts in L235 – 241. Regarding “how the Variables are distributed”, please note that the relevant visualizations are already presented in the article, including figure 1 which shows the geographic distribution of the crowd-submitted datapoints, and georeferenced locations of the other data sources, while Figures 6a and 6d shows the relationship of compared datasets at disaggregated admin1 (State) level and between market segments. The new map (Fig 6b) further presents spatial distribution of th

Attachment Submitted filename: Reviewer_Responses_JAdewopo.docx

# Editor report: Decision Letter 1

**DOI:** 10.1371/journal.pone.0320720.r004

**Authors:** Pradeep Paraman

19 Aug 2024

PONE-D-24-22683R1High-frequency food prices from Artificial Intelligence and crowdsourcing approach validated with groundtruth data in a fragile contextPLOS ONE

Dear Dr. Adewopo,

Thank you for submitting your manuscript to PLOS ONE. We have carefully reviewed the revised manuscript titled “High-frequency food prices from Artificial Intelligence and crowdsourcing approach validated with groundtruth data in a fragile context,” as well as the corresponding response letter submitted by the authors. While the authors have made significant improvements, there are several critical issues that remain unresolved and need to be addressed before the manuscript can be considered for publication in PLoS ONE.

Here are the main areas where the manuscript requires further revision:

**Title** :

1. **Ambiguity in Terminology** : The title includes the term "Artificial Intelligence" (AI) but does not specify what type of AI or the particular methods used (e.g., machine learning, neural networks). Providing more detail could clarify the technological approach employed.
2. **Vague Description of "Crowdsourcing Approach"** : The term "crowdsourcing approach" is broad and could benefit from more specificity. Detailing the type of crowdsourcing method used (e.g., survey-based, app-based submissions) would give readers a clearer understanding of the data collection process.
3. **Lack of Detail on "Groundtruth Data"** : The title mentions "groundtruth data" but does not indicate how this data was collected or its source. Including information about the type or source of groundtruth data could make the title more informative and precise.
4. **Inconsistency in Terminology** : The phrase "validated with groundtruth data" suggests a comparison or verification process. To improve clarity, the title could specify what aspects of the data were validated and how this validation supports the findings.
5. **Generalization of "Fragile Context"** : The term "fragile context" is used without explanation. Providing more context about what constitutes a fragile context in this study (e.g., economic instability, conflict areas) would help in understanding the specific relevance and application of the research.
6. **Potential for Overstatement** : The title might overstate the significance of the findings by using terms like "high-frequency" and "validated." If the validation process or the frequency of data collection is not as comprehensive as implied, this could lead to misleading interpretations.
7. **Complexity and Length** : The title is somewhat long and complex, which might make it less accessible. A more concise title that still conveys the main aspects of the study could be more effective in attracting and retaining reader interest.
8. **Repetition of Terms** : The title repeats the concept of validation and the use of alternative data sources. Streamlining these elements could enhance readability and focus.

**Suggested Revised Title** : "Validation of AI-Generated and Crowdsourced Food Price Data Against Ground Truth in Fragile Contexts"

**Abstract:**

- **Ambiguity in Data Innovation Details** : The abstract mentions data innovations such as AI and crowdsourcing but does not clearly explain how these methods were specifically applied or what distinguishes them from conventional methods. This lack of detail could leave readers unclear about the innovative aspects and practical implementation of these technologies.
- **Methodological Concerns** : While the abstract states that Pearson’s correlation and paired t-tests were used for validation, it does not provide information on the rationale for choosing these specific methods or how they were applied. This could raise questions about the robustness of the statistical analysis and whether other methods or metrics might have been more appropriate.
- **Insufficient Description of Validation Process** : The abstract notes the validation of AI-generated and crowdsourced data against ground truth data but does not elaborate on the specifics of the validation process. Details such as how the ground truth data was collected, the accuracy of the AI estimates, or the criteria for data validation are missing, which could impact the credibility of the findings.
- **Repetitive Statements** : There is a noticeable repetition in the abstract regarding the need for validation and the importance of real-time intelligence on food affordability. The redundancy of these statements could make the abstract less concise and focused.
- **Limited Insight into Findings** : Although the abstract highlights the comparability of AI and crowdsourced prices, it does not provide enough insight into the practical implications of these findings. For instance, how do these results impact decision-making processes or policy recommendations for food security in fragile contexts?
- **Generalization of Results** : The abstract reports high correlation values and similar inflation trends but does not discuss any potential limitations or contexts where these methods might not perform as well. This could lead to an overly optimistic view of the data innovations without acknowledging their limitations.
- **Lack of Contextual Relevance** : The abstract emphasizes the significance of high-frequency monitoring in fragile contexts but does not provide sufficient background on why the specific context of northern Nigeria was chosen. This could make it difficult for readers to understand the relevance and applicability of the study’s findings to other regions or situations.
- **Typographical and Grammar Issues** : There are minor grammatical errors and typographical issues in the abstract, such as “addresson” instead of “address,” and “wWe” instead of “We.” These issues could detract from the professionalism and clarity of the manuscript.

**Introduction section:**

1. **Overuse of Statistics Without Context** : The introduction begins with extensive statistics on food insecurity and poverty but lacks specific context or direct relevance to the study's focus. While these statistics are important, they could be better integrated to directly justify the need for the innovative approaches being discussed.
2. **Lack of Focus on the Core Problem** : The introduction covers a broad range of issues related to food insecurity, inflation, and the limitations of traditional data collection methods. However, it could benefit from a more focused discussion on why the specific problem of monitoring food prices in fragile contexts is critical and how the study addresses this issue uniquely.
3. **Complexity and Length** : The introduction is quite lengthy and dense, which may hinder reader engagement. It covers multiple aspects of food insecurity, data collection challenges, and innovations in data methods. Streamlining this section to focus on the most pertinent details about the study's objectives and methods would improve clarity.
4. **Insufficient Detail on Data Innovations** : While the introduction discusses crowdsourcing and AI, it lacks specific details on how these methods are applied within the study. More information on the practical implementation of these approaches and how they address the identified problems would enhance the reader's understanding.
5. **Overemphasis on General Background Information** : The introduction spends considerable time on general background information about food insecurity and data collection issues. This information, while relevant, could be condensed to provide a more direct and compelling rationale for the study's focus on validation of innovative data methods.
6. **Ambiguous Explanation of "Fragile Context"** : The term "fragile context" is used but not clearly defined. Providing a more precise definition or examples of what constitutes a fragile context in this study would help clarify why this setting is particularly relevant for the research.
7. **Lack of Clear Research Questions or Objectives** : The introduction outlines broad issues and the relevance of new data methods but does not clearly state the specific research questions or objectives of the study until later in the section. Presenting the research questions or hypotheses earlier would provide a clearer roadmap for the reader.
8. **Redundancy and Repetition** : Some points, such as the limitations of traditional data collection and the potential of new data methods, are repeated multiple times. This redundancy could be reduced to make the introduction more concise and impactful.
9. **Technical Jargon Without Explanation** : The introduction uses technical terms related to AI and data crowdsourcing (e.g., "Markov Chain Monte Carlo framework," "Cubist algorithm") without sufficient explanation. Providing a brief explanation or simplifying these terms would make the introduction more accessible to a broader audience.
10. **Unclear Transition to Study’s Specifics** : The transition from the general discussion of food insecurity and data challenges to the specific study focus is somewhat abrupt. A smoother transition that directly connects the broader issues to the study’s objectives would enhance coherence.

**Materials and Methods section:**

1. **Lack of Justification for State Selection** : The section does not provide a clear rationale for selecting these specific three states in northern Nigeria. While it mentions that these states have a high population and economic significance, it does not explain why they are particularly suitable for this study compared to other regions or how their selection influences the results.
2. **Unclear Relevance of Historical Context** : The historical context of insecurity and agricultural practices is mentioned but not explicitly linked to the study’s objectives. While this information is relevant, it should be clearly tied to how these factors impact data collection, price monitoring, or the study's outcomes.
3. **Ambiguity in Data Overlap Description** : The description of the overlap between World Bank’s RTP data and crowdsourced submissions is vague. It would be helpful to provide specific details on how this overlap is determined, including the criteria used to select the data points and the temporal and spatial alignment of the datasets.
4. **Insufficient Detail on Data Collection Methods** : The section mentions data collection from crowdsourcing and AI estimation but lacks detailed information on how these methods were implemented in practice. Specifics about the process, quality control measures, and the frequency of data collection would provide a clearer understanding of the methodology.
5. **Inconsistent Information on Market Types** : There is some repetition and inconsistency in describing market types and their characteristics. For example, the section describes both major markets and smaller village markets but does not clearly differentiate how these various market types are factored into the analysis.
6. **Inadequate Explanation of Price Transfer Influence** : The potential influence of vendors owning multiple stores on spatial price transfer is mentioned but not elaborated. A more detailed explanation of how this might affect the study’s findings would enhance understanding of potential biases or variations in the data.
7. **Lack of Detail on AI and Crowdsourcing Methods** : The section does not sufficiently describe the specific methods used by the AI algorithms and crowdsourcing platforms. For example, it would be beneficial to include information on the types of AI models used, data preprocessing steps, and how crowdsourced data integrity is ensured.
8. **Limited Description of Validation Process** : The process of how ground truth prices were compared to AI-generated and crowdsourced prices is not adequately described. Details on the validation criteria, statistical methods used, and how discrepancies are handled would provide a clearer picture of the study’s rigor.
9. **Map and Data Source Details** : The description of Figure 1 and the data sources is somewhat disjointed. It would be better to integrate the figure description more seamlessly with the text and ensure that the source of the map data (GADM) is presented clearly and contextually.
10. **Potential Biases in Market Coverage** : The section does not address any potential biases in the selection of market locations or the impact of these biases on the study results. Discussing how representative the selected markets are of the broader region would help to contextualize the findings.

**Data Description section:**

1. **Lack of Clarity on Data Integration Process** : The section briefly mentions coupling and wrangling datasets but lacks specific details on the methodology used to integrate data from different sources. Information on how inconsistencies and data mismatches were resolved is missing, which raises concerns about the reliability of the combined dataset.
2. **Ambiguity in Crowdsourcing Data Collection** : The description of crowdsourced data collection lacks detail on how data quality was ensured. For example, how were the volunteer submissions verified for accuracy? There is no mention of any quality control measures or validation processes to address potential inaccuracies or biases from volunteer submissions.
3. **Insufficient Information on Metadata** : While it is noted that unpublished metadata includes the ID of data submitters, the significance of this metadata is not explained. Understanding how this metadata was used to validate or cross-check the data is crucial, but this is not addressed in the description.
4. **Potential Bias in Enumerator Data** : Although the section mentions that enumerators were trained and dispersed across locations, it does not provide sufficient details on how their data might have been influenced by personal biases or logistical challenges. Further information on the training process, data collection protocols, and monitoring for consistency would be beneficial.
5. **Inadequate Explanation of Data Wrangling** : The term "wrangled" is used without elaboration. The description lacks specific details on the data wrangling techniques applied, including how raw data were cleaned, standardized, or transformed before analysis. This omission could impact the understanding of how robust and reliable the final dataset is.
6. **Unclear Standardization Process** : The explanation of how prices were standardized across various market segments and local measures is vague. Detailed information on the conversion units and standardization procedures is necessary to assess the accuracy and comparability of the price data.
7. **No Mention of Data Validation for AI-Estimated Prices** : There is no information on how the accuracy of the AI-estimated prices was validated against ground truth data or other benchmarks. Without this, it is difficult to gauge the reliability of the AI-generated data used in the analysis.
8. **Ambiguity in AI Methodology Description** : The description of the AI methodology is quite technical but lacks clarity on how the machine learning models specifically address local price dynamics. It would be helpful to include more information on the types of models used, the parameters, and how they were tuned to handle localized shocks.
9. **Limited Justification for Commodity Selection** : The focus on rice and maize is mentioned, but the rationale behind selecting these particular commodities over others is not discussed. Justifying why these staples were chosen and how their selection impacts the study would strengthen the methodology.
10. **Generalization of Market Coverage** : The description of market types and their coverage is somewhat generalized. More precise details on the number and type of markets covered, and how these markets are representative of the broader region, would provide better insight into the data's comprehensiveness and applicability.

**Statistical Analyses section:**

1. **Limited Description of Statistical Methods** : The section provides a basic overview of the statistical methods used but lacks detailed explanations of why specific techniques were chosen and how they address the study’s objectives. For example, while Pearson’s correlation and paired t-tests are mentioned, there is no rationale given for their selection over other possible statistical tests.
2. **Potential Issues with Outlier Detection** : The use of Tukey's outlier detection method is mentioned, but there is no discussion on how outliers could impact the results or the implications of removing 1.3% of the dataset. The effectiveness of this method in capturing all relevant outliers and its impact on the data’s integrity is not addressed.
3. **Assumption of Independence** : The assumption that the datasets are completely independent may oversimplify the situation. Given that the data sources are interrelated (crowdsourced and enumerator-submitted), this assumption could overlook potential dependencies or correlations that might affect the validity of the results.
4. **Lack of Justification for Time-Lag Analysis** : While time-lagged relationships are evaluated, the rationale for this specific analysis is not clearly justified. It would be helpful to explain why time-lags are important in this context and how they contribute to understanding the correlation between AI-estimated and crowdsourced prices.
5. **Methodological Concerns with Centroid Calculation** : The process of computing the centroid of administrative boundaries to assess correlations with AI-imputed market locations may introduce inaccuracies. The choice of the closest market location and its impact on correlation results is not thoroughly discussed.
6. **Inadequate Detail on Correlation Metrics** : The description of the correlation coefficient (R) and coefficient of determination (r²) is basic and does not include potential limitations or interpretations of these metrics in this context. It is important to discuss how these metrics are applied and their limitations in evaluating the data.
7. **No Mention of Assumptions in Statistical Tests** : The section mentions paired sample t-tests but does not discuss the assumptions underlying these tests, such as normality and equal variances. Ignoring these assumptions may lead to incorrect conclusions if the data do not meet the required conditions.
8. **Potential Over-Reliance on Statistical Significance** : The focus on statistical significance (p-values) may overlook practical significance and the real-world relevance of the findings. It’s important to consider both the statistical and practical implications of the results.
9. **Ambiguity in Statistical Software Usage** : The reference to using "relevant R packages and functions" is vague. Specific details on the packages and functions used would provide clarity on the methods and their appropriateness for the analyses conducted.
10. **Lack of Discussion on Data Aggregation** : The analysis involves aggregating data to daily, weekly, and monthly averages, but there is no discussion on how this aggregation might affect the results. Aggregation can obscure significant fluctuations and variations, and the potential impact of this on the analysis should be addressed.
11. **Unclear Explanation of Results Interpretation** : The section lacks information on how the results from the statistical analyses were interpreted and how they contribute to the overall conclusions of the study. More insight into how the results align with the research objectives would be useful.

**Results section:**

1. **Lack of Detailed Statistical Analysis** : The results are presented with limited detail on the specific statistical analyses performed. For instance, while correlation coefficients are mentioned, there is no explanation of how these values are interpreted or their statistical significance. A more thorough presentation of statistical findings, including measures of uncertainty and confidence intervals, would enhance the clarity and reliability of the results.
2. **Inadequate Comparison of Sub-Types** : The differentiation of commodities into sub-types (e.g., yellow maize, white maize) is mentioned, but the results section lacks a detailed comparison of these sub-types. The impact of these distinctions on the analysis and how they affect the overall results are not clearly discussed. This could lead to confusion about the relevance and accuracy of the comparisons made.
3. **Issues with Data Aggregation** : The results discuss converting various units to a per-kilogram basis, but there is no mention of how this conversion might impact the accuracy of the comparisons. The fact that discounts and other pricing factors associated with larger quantities are not controlled for could lead to misleading conclusions about price trends.
4. **Confusing Presentation of AI-Estimated Prices** : The mention of AI-estimated prices with a range of metrics (OHLC) alongside the intra-month interval data from crowdsourced and enumerator sources could be confusing. The rationale for using specific price metrics (e.g., closing prices) and their impact on the results are not sufficiently clarified.
5. **Unclear Justification for Focus Areas** : The choice to focus on certain commodities (e.g., yellow maize) over others is not well justified. The rationale for selecting these particular commodities and the implications for the broader analysis are not discussed, which may leave readers questioning the relevance of the focus areas.
6. **Lack of Integration of Findings** : The section seems to separate the results of different analyses (enumerator vs. crowdsourced prices, AI-estimated vs. crowdsourced prices) without clearly integrating these findings. A more cohesive presentation that links the results from different comparisons would help in understanding the overall implications.
7. **Limited Discussion of Nuanced Aspects** : The exploration of nuanced aspects relative to sub-national geography, market segments, or commodity sub-types is mentioned but not elaborated upon. Detailed insights into how these factors influence the results are missing, which could provide a deeper understanding of the data.
8. **Insufficient Detail on Data Quality and Limitations** : There is little discussion on the potential quality issues or limitations of the data sources. For example, the reliability of the crowdsourced data and its potential biases are not addressed, which could impact the credibility of the results.
9. **Sparse Explanation of Supplementary Material** : The mention of additional insights in the supplementary material is brief, with no detail on what this material contains or how it supports the main findings. A summary of key points from the supplementary material would be useful for understanding the complete scope of the results.
10. **Missing Contextualization of Findings** : The results are presented without sufficient context or comparison to existing literature. Including comparisons to previous studies or industry benchmarks would help in contextualizing the findings and assessing their significance.

**Figure 2 results section:**

1. **Lack of Context for Correlation Coefficients** : The correlation coefficients reported (e.g., 0.69 to 0.94) are presented without sufficient context or explanation. It is unclear how these values compare to benchmarks or what they indicate about the practical significance of the correlations. A more detailed interpretation of these coefficients would help in understanding their relevance.
2. **Inconsistent Data Presentation** : The results mention significant correlation between data sources but also highlight variability in prices. The text should clarify how variability impacts the overall correlation and whether the apparent high correlation at a monthly level masks important differences observed at daily or weekly intervals.
3. **Overemphasis on Statistical Significance** : The emphasis on p-values and statistical significance might obscure practical significance. For instance, the differences in mean prices are not statistically significant (p=0.40), yet this might not address practical implications of price differences for stakeholders.
4. **Limited Discussion on Data Noise** : While the text acknowledges data noisiness at higher resolution (daily and weekly), it does not explain how this noise affects the validity of the correlations. There is insufficient discussion on how data noise might impact the reliability of the results and whether any adjustments were made to account for it.
5. **Ambiguous Variable Definitions** : Terms such as "ground truth" and "crowdsourced" are used without clear definitions or differentiation. The results should explicitly define these terms to avoid confusion and ensure that the reader understands the different data sources and their implications.
6. **Inadequate Visualization Explanation** : The results refer to Figures 2B and 2C without sufficient explanation of what these figures specifically illustrate. A more detailed description of the figures and how they relate to the textual results would improve comprehension.
7. **Sparse Comparison with AI-Estimated Prices** : The focus seems to be more on comparing crowdsourced and enumerator prices rather than providing a thorough analysis of the AI-estimated prices. More emphasis on how AI-estimated prices align or diverge from the other datasets would be beneficial.
8. **Lack of Analysis on Temporal Trends** : Although temporal trends are mentioned, there is limited analysis on how these trends might affect the results or their interpretation. For instance, how do temporal variations in pricing affect the overall findings?
9. **Inconsistent Use of Statistical Metrics** : The text uses various statistical metrics (e.g., R, r²) without clearly explaining why these metrics were chosen or how they are best interpreted in the context of this study. There should be more rationale behind the choice of metrics and their application.
10. **Omission of Limitations and Potential Biases** : There is no discussion of potential limitations or biases in the data sources. Addressing these aspects is crucial for understanding the reliability of the results and for acknowledging any potential sources of error or bias in the data collection process.
11. **Inadequate Insight into Sub-Type Variability** : While there is mention of high variability in yellow maize prices, the implications of this variability are not fully explored. The results should delve deeper into how this variability affects the comparison and what it means for the overall analysis.
12. **Unclear Impact of Conversion Methods** : The conversion of prices to a per-kilogram basis is noted but not thoroughly discussed. The impact of this conversion method on the comparison results should be elaborated to ensure that any potential biases or inaccuracies are addressed.

**Crowdsourced vs. AI-Estimated Commodity Price" section:**

1. **Redundancy in Explanation** : The section reiterates similar points about the strong relationship between crowdsourced and AI-estimated prices multiple times (e.g., lines 399, 403, 409). This redundancy could have been avoided by consolidating these points into a more concise summary, enhancing readability and focus.
2. **Inconsistent Analysis and Interpretation** : The analysis claims a near-perfect correlation (r² = 0.94) between crowdsourced and AI-estimated prices, but it also highlights month-to-month discrepancies (e.g., June 2021). The interpretation does not sufficiently address how these discrepancies impact the overall assessment of data reliability and validity.
3. **Limited Discussion on Discrepancies** : While the section notes a specific instance where AI-estimated prices declined and crowdsourced prices increased significantly, it does not delve into the reasons behind this divergence or its implications. There should be a deeper analysis of why such discrepancies occur and how they affect the overall findings.
4. **Overemphasis on Statistical Metrics** : The focus on statistical metrics such as r² and p-values may overshadow practical implications. The practical significance of the near-perfect correlation and the month-to-month variations is not clearly explained, leaving the reader questioning the real-world relevance of these findings.
5. **Lack of Contextual Explanation** : The section mentions that the AI-estimated prices are higher in certain contexts compared to crowdsourced prices without providing a clear explanation for why this is the case. More context on factors influencing price differences (e.g., market conditions, data collection methods) would improve understanding.
6. **Ambiguous Temporal Analysis** : The time-lagged analysis shows a decline in correlation with increasing lag, but the explanation is somewhat vague. It should include a more detailed discussion on the potential reasons for this decline and its implications for the reliability of the AI estimates over time.
7. **Inadequate Detail on Data Filtering** : The discussion on filtering crowdsourced prices by market type (farmgate, wholesale, retail) lacks detail. It does not explain the methodology for filtering or how these different market segments might affect the price comparison with AI estimates.
8. **Insufficient Discussion on Data Gaps** : The mention of "inherent temporal gaps" in crowdsourced data is not sufficiently explored. The impact of these gaps on the overall analysis and how they might affect the correlation with AI estimates should be discussed in more detail.
9. **Misalignment Between Figures and Text** : The figures mentioned (Figure 4 and Figure 5) are not fully integrated into the text. The section describes trends and relationships but does not adequately explain or interpret the figures, leaving the reader without a clear understanding of what the visual data represent.
10. **Potential Biases and Limitations Not Addressed** : The analysis does not address potential biases or limitations in the crowdsourced or AI-estimated data. Discussing these factors is crucial for assessing the reliability of the results and understanding any potential sources of error.
11. **Failure to Address Practical Implications** : While statistical relationships are discussed, the practical implications of these relationships for stakeholders or decision-makers are not addressed. The section should include a discussion on how these findings impact real-world applications, such as market monitoring or price forecasting.
12. **Lack of Clarity on Data Sources** : There is some confusion about the specifics of the data sources used for AI-estimation and crowdsourcing. Clearer definitions and explanations of these sources would help in understanding the context and reliability of the data.

**Unraveling Relationships by Location, Commodity Type, and Market Segments section:**

- **Overgeneralization of Results** : The section claims strong and consistent relationships between crowdsourced and AI-estimated prices across various levels of granularity but does not provide sufficient details on any potential deviations or inconsistencies at lower geographic levels or among different commodity sub-types. This overgeneralization may overlook important nuances and variations.
- **Lack of Detail on Methodology** : The process of disaggregating data by geographic level, commodity type, and market segment is described superficially. There is insufficient detail on how these analyses were conducted, including specific methods for handling different data segments and potential issues encountered during disaggregation.
- **Inadequate Discussion on Variability** : While the section mentions that the prices are comparable, it lacks a thorough discussion on the reasons behind any variability in prices, especially in terms of regional differences or market segments. The section should address why certain areas or segments might show different price trends or discrepancies.
- **Superficial Analysis of Market Segments** : The analysis mentions that market segments show price variations (Farmgate < Wholesale < Retail) but does not explore the implications of these differences. The potential impact of these variations on the overall findings is not discussed, leaving a gap in understanding how market segment differences might affect price comparisons.
- **Failure to Address Outliers** : The strong correlations reported (R values between 0.72 and 0.99) could be misleading if outliers or anomalies are not adequately addressed. The section does not mention whether outliers were examined or how they might influence the observed relationships.
- **Inconsistent Presentation of Figures** : The text references several figures (e.g., Figure 6a, Figure 6b) without providing a clear explanation of what these figures illustrate or how they support the textual analysis. This lack of integration makes it difficult for readers to understand the connection between the figures and the reported results.
- **Overreliance on Statistical Metrics** : The focus on correlation coefficients (R values) and their statistical significance may overshadow the practical significance of the findings. The practical implications of these strong correlations for real-world applications and decision-making are not adequately discussed.
- **Lack of Contextual Insight** : The section lacks contextual information about the economic or market conditions in the regions analyzed. Understanding the broader market dynamics or external factors that could influence price data is crucial for interpreting the results.
- **Unexplored Data Limitations** : There is no mention of any limitations or potential biases in the data sources or analysis methods. Addressing possible limitations would provide a more balanced view of the reliability and robustness of the findings.
- **Insufficient Exploration of Discrepancies** : While the section notes that there are slight differences in prices between crowdsourced and AI-estimated data, it does not delve into the potential reasons for these discrepancies. A more detailed exploration of why such differences exist and their implications would enhance the analysis.
- **Potential Lack of Replicability** : The section does not discuss whether the observed relationships are replicable across other datasets or regions. This raises concerns about the generalizability of the findings.
- **Ambiguous Terminology** : Terms like "fragile context" and "co-variance" are not clearly defined or explained. Ambiguous terminology can lead to confusion and misinterpretation of the results.
- **Inadequate Comparison to Previous Studies** : The section does not compare the findings to previous studies or benchmarks, which would provide additional context and validation for the results.

**Discussion and Conclusion section:**

1. **Lack of Critical Evaluation** : The discussion overly emphasizes the positive outcomes of the analysis without sufficiently addressing the limitations or potential weaknesses of the crowdsourced and AI-estimated data. A more balanced view should include a critical evaluation of potential drawbacks or uncertainties in the data and methodologies.
2. **Overemphasis on Correlation** : The discussion highlights the high correlation between data sources, but it does not sufficiently explore the practical implications of these correlations. Correlation does not imply causation, and the discussion should address whether the observed relationships truly reflect accurate and reliable price assessments or merely superficial agreement.
3. **Insufficient Analysis of Variability** : While the analysis demonstrates strong correlations, it fails to delve into the variability or inconsistencies observed in the data, particularly in the context of temporal and spatial differences. A deeper exploration of how variability impacts the reliability of both data sources would strengthen the discussion.
4. **Lack of Consideration for External Factors** : The impact of external factors such as market disruptions, economic changes, or the COVID-19 pandemic is mentioned but not thoroughly analyzed. The discussion should better explore how these factors might have influenced the data and the robustness of the findings.
5. **Ambiguous References to Figures** : The text refers to several figures (e.g., Figure 1, Figure 4A) without clearly explaining their relevance or findings in relation to the discussion. The figures should be more explicitly integrated into the discussion to clarify their contribution to the overall analysis.
6. **Superficial Examination of Data Gaps** : The acknowledgment of gaps in crowdsourced data due to discontinuous phases is brief and lacks detail. The discussion should provide a more thorough examination of how these gaps might affect the overall validity and continuity of the data analysis.
7. **Overstated Claims about Crowdsourcing and AI** : The discussion makes strong claims about the reliability and potential of crowdsourced data and AI estimates but does not sufficiently address the challenges and limitations of these innovative methods. A more nuanced discussion of the limitations and potential issues with crowdsourced and AI-generated data would be valuable.
8. **Unclear Practical Implications** : The practical implications of the findings for food security and market monitoring are mentioned but not fully developed. The discussion should provide more specific examples of how the results can be applied in real-world scenarios, particularly in fragile or low-resource settings.
9. **Overreliance on Statistical Significance** : The emphasis on statistical significance (e.g., p-values, r² values) may overshadow practical significance and real-world relevance. The discussion should consider whether the statistically significant correlations translate into meaningful insights for stakeholders.
10. **Inadequate Exploration of Data Source Integration** : The discussion briefly mentions the potential for integrating crowdsourced and AI-generated data but does not provide a detailed plan or framework for how such integration might be practically achieved. A more concrete proposal for integrating these data sources would enhance the discussion.
11. **Failure to Address Methodological Limitations** : The discussion does not adequately address potential methodological limitations, such as biases in data collection or issues with the AI algorithms used for estimation. Understanding these limitations is crucial for assessing the reliability and generalizability of the findings.
12. **Limited Insight into Data Quality** : There is insufficient discussion on the quality of the crowdsourced data compared to traditional enumerator data. While the results suggest strong agreement, the discussion should address how data quality was assessed and whether there are any concerns about data accuracy or completeness.
13. **Lack of Future Research Directions** : The section does not provide clear recommendations for future research or improvements. Discussing potential future studies, improvements in data collection methods, or advancements in AI algorithms would provide valuable direction for further investigation.
14. **Overlooked Ethical Considerations** : The discussion does not mention ethical considerations related to crowdsourcing data, such as data privacy and the potential for misuse. Addressing these concerns is important for ensuring the responsible use of innovative data collection methods.
15. **Limited Contextualization** : The analysis focuses on a specific geographic context (northern Nigeria) but does not consider how the findings might differ in other regions or countries. A broader contextualization of the results would enhance the understanding of their applicability and relevance.

**References:**

1. **Lack of Cohesion and Structure** : The references span a broad range of topics, from food security updates to machine learning applications. This wide scope might indicate a lack of focus or cohesion in the literature review or research topic. A more streamlined selection of sources could improve clarity and relevance.
2. **Outdated or Less Relevant Sources** : Some sources, such as the 2002 Regional Economist article or the 2015 FAO report, might be considered outdated in the context of current food security issues. Given the rapid developments in this field, newer research or more recent data might provide more accurate insights.
3. **Overemphasis on Certain Types of Data** : The heavy reliance on World Bank reports and crowdsourced data might limit the diversity of perspectives and methodologies. While these sources are valuable, integrating a broader range of data types and sources could provide a more comprehensive view of food security issues.
4. **Potential Bias in Data Sources** : Some references are from organizations with specific agendas or funding sources, which might introduce bias into the data or analysis. For instance, reports from the World Bank or the IMF might have particular economic perspectives that could affect their interpretations.
5. **Technical and Niche Focus** : Several references, particularly those on advanced statistical methods or machine learning (e.g., references 24, 25, 28), may be too technical for some audiences. This focus might alienate readers who are less familiar with these methodologies or who are more interested in practical, policy-oriented insights.
6. **Inconsistencies in Data and Analysis** : There may be inconsistencies in the data presented across different sources. For example, disparities between crowdsourced data and official statistics could lead to conflicting conclusions, making it challenging to derive clear policy recommendations.
7. **Limited Geographic Focus** : Many references are focused on specific regions or countries (e.g., Nigeria, Africa), which might limit the applicability of their findings to other contexts. A more global perspective or additional regional studies could enhance the applicability and relevance of the research.
8. **Lack of Critical Analysis** : The references primarily present data and findings without a critical analysis of the limitations or potential biases in the studies. A more critical approach could offer deeper insights into the reliability and applicability of the findings.

 **Supporting information text:**

1. **Lack of Clarity in Figure Descriptions** :
  - **S1 Fig** : The description is vague and lacks specifics on what "cohesion of price signal" means. It’s unclear how the improvement of price signal coherence is measured or presented.
  - **S2 Fig** : The term "fragile context" is used without explanation. The difference in the number of geolocated market points for crowdsourced versus AI-estimated prices could be misleading without further context on why this discrepancy exists.
  - **S3 Fig** : The description of the relationship between crowdsourced and AI-estimated prices is not detailed enough. There is no explanation of what “disaggregated by State and commodity subtype” specifically entails or how it impacts the analysis.
2. **Inconsistencies and Unexplained Terms** :
  - **S1 Fig** : The reference to "intraday datapoints" and how they were averaged into different time intervals is not fully explained. The impact of averaging on data accuracy or relevance should be clarified.
  - **S4 Fig** : The term "log-transformed counts" might be confusing without additional context on why log-transformation was necessary and how it affects data interpretation.
3. **Potential Data Access Issues** :
  - The text states that datasets are accessible through various repositories but does not provide direct links to these datasets. This could be frustrating for readers attempting to verify or access the data. The URLs provided (e.g., GitHub and World Bank) should be clearly accessible and not require additional search.
4. **Inadequate Description of Data Availability** :
  - The description of data availability is dense and somewhat unclear. It mixes different data sources and repositories without clear separation or explanation of how each dataset contributes to the overall analysis. This could lead to confusion about where to find specific types of data.
5. **Missing Details on Data Handling** :
  - The text mentions that detailed intraday datasets are accessible but does not specify what these datasets contain or how they differ from the daily averages. There is also no explanation of how data was processed or filtered, which is important for reproducibility and understanding the results.
6. **Redundancy and Formatting Issues** :
  - There is a noticeable inconsistency in the formatting, such as the repeated "Formatted: Font" and "Formatted: Line spacing" statements, which detract from the professionalism of the document. These formatting issues can be distracting and detract from the content’s credibility.
7. **Inadequate Explanation of Methodology** :
  - The description of how the correlation analysis was performed is brief and lacks detail on the statistical methods used. This omission makes it difficult for readers to fully understand the robustness of the analysis and the reliability of the findings.
8. **Unclear Presentation of Figures and Data** :
  - The text refers to various figures and their content but does not provide a clear explanation of how these figures are related to each other or how they collectively support the study’s conclusions. More detailed captions and explanations for each figure would help clarify their significance and interconnections.

**The main areas where the manuscript requires further revision:**

1. **Clarity and Detail in Supporting Information** :
  - The descriptions of the figures (S1-S4) are still vague and lack critical detail. For instance, the explanations regarding the "cohesion of price signal" and "log-transformed counts" need further elaboration. The authors should provide clearer explanations of the data presented and the rationale behind the methods used.
2. **Consistency and Completeness of Data Access Information** :
  - The information on data availability is incomplete and somewhat confusing. The manuscript should include direct, functional links to all data repositories and ensure that the descriptions of datasets and their availability are clear and accurate.
3. **Explanation of Statistical Methods** :
  - There is insufficient detail on the statistical methods used for correlation analysis. The authors should expand on how they handled assumptions, and describe any statistical techniques used to analyze time-lagged relationships between datasets.
4. **Addressing Review Comments Thoroughly** :
  - While some reviewer comments have been addressed, others have not been fully incorporated. For example, the discussion on unexpected trends and outliers remains insufficient. The manuscript should be revised to better reflect the reviewers' suggestions and ensure that all aspects of the feedback are comprehensively addressed.
5. **Improving Figure and Data Presentation** :
  - The presentation of figures and related data needs to be improved for better clarity. Ensure that figures are clearly referenced in the text and that their captions provide sufficient context to understand the data without ambiguity.
6. **Formatting and Professionalism** :
  - The manuscript has several formatting issues that need to be corrected. Ensure that the formatting is consistent throughout, and that any extraneous formatting notes or errors are removed.

We request that the authors revise the manuscript to address these concerns and resubmit it for further consideration. Please ensure that the revised manuscript includes a detailed response letter that explicitly addresses each of the points raised in this letter.

Thank you for your attention to these matters. 

Please submit your revised manuscript by  Oct 03 2024 11:59PM. If you will need more time than this to complete your revisions, please reply to this message or contact the journal office at plosone@plos.org . When you're ready to submit your revision, log on to https://www.editorialmanager.com/pone/ and select the 'Submissions Needing Revision' folder to locate your manuscript file.

Please include the following items when submitting your revised manuscript:

- A rebuttal letter that responds to each point raised by the academic editor and reviewer(s). You should upload this letter as a separate file labeled 'Response to Reviewers'.
- A marked-up copy of your manuscript that highlights changes made to the original version. You should upload this as a separate file labeled 'Revised Manuscript with Track Changes'.
- An unmarked version of your revised paper without tracked changes. You should upload this as a separate file labeled 'Manuscript'.

If you would like to make changes to your financial disclosure, please include your updated statement in your cover letter. Guidelines for resubmitting your figure files are available below the reviewer comments at the end of this letter.

If applicable, we recommend that you deposit your laboratory protocols in protocols.io to enhance the reproducibility of your results. Protocols.io assigns your protocol its own identifier (DOI) so that it can be cited independently in the future. For instructions see: https://journals.plos.org/plosone/s/submission-guidelines#loc-laboratory-protocols . Additionally, PLOS ONE offers an option for publishing peer-reviewed Lab Protocol articles, which describe protocols hosted on protocols.io. Read more information on sharing protocols at https://plos.org/protocols?utm_medium=editorial-email&utm_source=authorletters&utm_campaign=protocols .

We look forward to receiving your revised manuscript.

Kind regards,

Pradeep Paraman

Academic Editor

PLOS ONE

Journal Requirements:

Please review your reference list to ensure that it is complete and correct. If you have cited papers that have been retracted, please include the rationale for doing so in the manuscript text, or remove these references and replace them with relevant current references. Any changes to the reference list should be mentioned in the rebuttal letter that accompanies your revised manuscript. If you need to cite a retracted article, indicate the article’s retracted status in the References list and also include a citation and full reference for the retraction notice.

[Note: HTML markup is below. Please do not edit.]

[NOTE: If reviewer comments were submitted as an attachment file, they will be attached to this email and accessible via the submission site. Please log into your account, locate the manuscript record, and check for the action link "View Attachments". If this link does not appear, there are no attachment files.]

While revising your submission, please upload your figure files to the Preflight Analysis and Conversion Engine (PACE) digital diagnostic tool, https://pacev2.apexcovantage.com/ . PACE helps ensure that figures meet PLOS requirements. To use PACE, you must first register as a user. Registration is free. Then, login and navigate to the UPLOAD tab, where you will find detailed instructions on how to use the tool. If you encounter any issues or have any questions when using PACE, please email PLOS at figures@plos.org . Please note that Supporting Information files do not need this step.

# Author comment: Author response to Decision Letter 2

**DOI:** 10.1371/journal.pone.0320720.r005

15 Jan 2025

Dear Reviewer/Editor,

Thank you very much for your thorough review of our paper and the insightful suggestions you provided. We have carefully considered each of your comments and made revisions throughout the manuscript, which we believe have significantly enhanced its quality. Your feedback has been instrumental in improving the clarity, depth, and overall presentation of our work.

We have structured our response as follows: we first provide a single response to each of the 14 groups of comments for a more streamlined overview. This is followed by specific point-by-point answers to each of the 141 individual comments, presented in a comment/response format. To ensure clarity and conciseness, we sometimes use shortened versions of your comments that reflect our understanding and the actions we took. This approach allowed us to address all feedback comprehensively while keeping the response letter concise, as including the full text of each comment would have resulted in a response letter more than twice the length of our article.

We hope that our revisions and the detailed explanations provided meet your expectations, and we sincerely appreciate your time and effort in reviewing our manuscript.

Thank you once again for your valuable feedback.

Title:

Response:

Thank you for your detailed feedback and suggestions on the title. We have revised it to: “AI-imputed and crowdsourced price data show strong agreement with traditional price surveys in data-scarce environments” to better reflect the focus and contributions of our study. In response to your comments, we made several key revisions. We specified the AI methods used, emphasizing the use of hybrid machine learning models, and clarified the crowdsourcing approach, particularly the smartphone-based data collection process. Additionally, we included more information about how ground truth data was collected through enumerators. To avoid potential confusion, we removed terms like "fragile" and "high-frequency," ensuring that the title remains both concise and precise, while avoiding any redundancy. These changes aim to enhance the clarity and readability of our manuscript, making it more accessible to the journal's audience.

1. Ambiguity in Terminology:

• Comment: The title includes the term "Artificial Intelligence" (AI) but does not specify what type of AI or the particular methods used (e.g., machine learning, neural networks). Providing more detail could clarify the technological approach employed.

• Response: We have included additional information about the type of AI used in the manuscript [lines 237-266: “The imputation process employs hybrid machine learning models that integrate local relationships within specific regions of the feature space, akin to the concept of local receptive fields in convolutional neural networks”]. Our study explores the feasibility of AI-imputation methods broadly, using publishedAI-imputed price datasets; therefore, our revised title captures this broader focus without specifying a single approach.

2. Vague Description of "Crowdsourcing Approach":

• Comment: The term "crowdsourcing approach" is broad and could benefit from more specificity. Detailing the type of crowdsourcing method used (e.g., survey-based, app-based submissions) would give readers a clearer understanding of the data collection process.

• Response: We have clarified the details in the manuscript [lines 213-236: “. The volunteers were remotely invited and onboarded to submit commodity price data, including maize and rice, through the ODK app”]. While most crowdsourcing is conducted through mobile devices, we felt using the term "mobile" in the title might cause confusion with other mobile data types, like location data, which differ in context and methodology.

3. Lack of Detail on "Groundtruth Data":

• Comment: The title mentions "groundtruth data" but does not indicate how this data was collected or its source. Including information about the type or source of groundtruth data could make the title more informative and precise.

• Response: We have added more details on the collection of ground truth data in the text [lines 161-167], describing the role of enumerators and their submission methods.

4. Inconsistency in Terminology:

• Comment: The phrase "validated with groundtruth data" suggests a comparison or verification process. To improve clarity, the title could specify what aspects of the data were validated and how this validation supports the findings.

• Response: We have revised the title to remove this ambiguity and ensure a clearer representation of the study’s approach and findings.

5. Generalization of "Fragile Context":

• Comment: The term "fragile context" is used without explanation. Providing more context about what constitutes a fragile context in this study (e.g., economic instability, conflict areas) would help in understanding the specific relevance and application of the research.

• Response: We have revised the title to exclude the term “fragile” and made changes in the abstract and throughout the manuscript to better explain the specific context [e.g., “northern Nigeria, a region affected by conflict, food insecurity, and data scarcity”]. We recognize that "fragile" is a term with a specific meaning in certain circles but may not be immediately clear to all readers.

6. Potential for Overstatement:

• Comment: The title might overstate the significance of the findings by using terms like "high-frequency" and "validated." If the validation process or the frequency of data collection is not as comprehensive as implied, this could lead to misleading interpretations.

• Response: We have removed the terms "high-frequency" and "validated" from the title to avoid any potential overstatement.

7. Complexity and Length:

• Comment: The title is somewhat long and complex, which might make it less accessible. A more concise title that still conveys the main aspects of the study could be more effective in attracting and retaining reader interest.

• Response: The revised title aims to balance conciseness with clarity, emphasizing the study’s focus while keeping the length in check.

8. Repetition of Terms:

• Comment: The title repeats the concept of validation and the use of alternative data sources. Streamlining these elements could enhance readability and focus.

• Response: We have streamlined the title to avoid repetition and focus on the key aspects of our study.

Abstract:

Response:

Thank you for your thorough review and constructive feedback on the abstract. We have fully revised it to improve readability, clarity, and detail, addressing the specific issues you raised. Key revisions include adding more information on how the AI and crowdsourcing methods were applied, justifying our choice of validation methods like Pearson’s correlation and paired t-tests, and providing clearer details on the validation process itself, including how ground truth data was collected and analyzed. We also streamlined the abstract to remove repetitive statements and enhance focus. Additionally, we emphasized the practical implications of our findings, particularly in regions with limited capacity for conventional surveys, and highlighted the relevance of our study to northern Nigeria and similar contexts. We also resolved grammatical and typographical issues identified, providing both a clean and tracked version for your review. We believe these changes have significantly strengthened the abstract, and we appreciate your feedback in helping to improve our manuscript.

1. Ambiguity in Data Innovation Details:

• Comment: The abstract mentions data innovations such as AI and crowdsourcing but does not clearly explain how these methods were specifically applied or what distinguishes them from conventional methods.

• Response: We have revised the abstract to include more details about the specific application and justification of the methods. Additionally, we have simplified the language for improved clarity, focusing on the distinctive aspects of AI and crowdsourcing compared to traditional methods.

2. Methodological Concerns:

• Comment: While the abstract states that Pearson’s correlation and paired t-tests were used for validation, it does not provide information on the rationale for choosing these specific methods or how they were applied.

• Response: We have revised the abstract to briefly justify the choice of these methods [lines 30-38]. Further details on the rationale and application of the methods are now included in the main text, including supporting information.

3. Insufficient Description of Validation Process:

• Comment: The abstract notes the validation of AI-generated and crowdsourced data against ground truth data but lacks details about the validation process.

• Response: We have updated the abstract to include more specific details on the validation process, such as how the ground truth data was collected, the time periods compared, and key item pair-level validation results. These additions aim to enhance the credibility of the findings.

4. Repetitive Statements:

• Comment: The abstract contains repetitive statements regarding the need for validation and the importance of real-time intelligence on food affordability.

• Response: The abstract has been revised to improve conciseness and eliminate redundancies. We have streamlined the text to ensure a sharper focus on the core findings and contributions.

5. Limited Insight into Findings:

• Comment: Although the abstract highlights the comparability of AI and crowdsourced prices, it lacks insight into the practical implications of these findings.

• Response: We have revised the latter part of the abstract to address the practical implications of our findings. This now emphasizes how our results provide crucial evidence to support the use of alternative data sources in decision-making processes, particularly in contexts with limited capacity for conventional price surveys [lines 39-43].

6. Generalization of Results:

• Comment: The abstract reports high correlation values and similar inflation trends but does not discuss potential limitations or contexts where these methods might be less effective.

• Response: We have adjusted the concluding sentences of the abstract to indicate the contexts in which these methods are most applicable. Specifically, we highlight their usefulness in subnational regions with limited monitoring capacity and where high-frequency local data is essential.

7. Lack of Contextual Relevance:

• Comment: The abstract emphasizes high-frequency monitoring in fragile contexts without providing sufficient background on the relevance of the study's specific focus on northern Nigeria.

• Response: We have provided additional context at the end of the abstract, explaining the relevance of the northern Nigeria setting and its broader implications for similar regions. This contextual relevance underscores why the study’s findings matter beyond this particular case.

8. Typographical and Grammar Issues:

• Comment: There are minor grammatical errors and typographical issues in the abstract, such as “addresson” instead of “address,” and “wWe” instead of “We.”

• Response: We have thoroughly copy-edited the paper, and these issues have been resolved. The examples you mentioned were artifacts of track changes, and we have now provided both a track changes version for transparency and a clean version for ease of reading.

Introduction section:

Response:

Thank you for your thorough review of the introduction. We have rewritten this section to be more focused on the core issues, reducing extraneous statistics, streamlining the text, and adding necessary details. Specifically, we removed unnecessary statistics, focusing instead on targeted data points that directly support the study’s focus. We clarified the link between data scarcity and development challenges, highlighting the critical need for timely food price monitoring in fragile contexts and emphasizing our study's unique contribution in this area. Additionally, we shortened the introduction to ensure a tighter focus on the study's objectives, included more specific details on AI and crowdsourcing methods, and provided a clearer explanation of the term “fragile context” with a preference for more precise language where possible. We also refined the presentation of our research question, removed redundant information, and reduced technical jargon for accessibility. Finally, we improved the transition between the general background and the study’s specific focus, ensuring a more logical flow. We believe these revisions have enhanced the clarity, focus, and overall presentation of the introduction, and we appreciate your feedback, which has greatly improved this section of our manuscript.

1. Overuse of Statistics Without Context:

• Comment: The introduction contains extensive statistics on food insecurity and poverty but lacks specific context or direct relevance to the study's focus.

• Response: We have revised the introduction to remove most extraneous statistics and have presented remaining data in a more targeted way. For instance, we now directly mention the item pairs for which statistics are presented, instead of providing a range for multiple comparisons. These adjustments help ensure that the statistics serve to directly support the study’s focus. Similar edits have been made throughout the paper for consistency.

2. Lack of Focus on the Core Problem:

• Comment: The introduction could benefit from a more focused discussion on why monitoring food prices in fragile contexts is critical and how the study uniquely addresses this issue.

• Response: We agree, and we have clarified the connection between data scarcity and development challenges, citing recent quantitative studies on the subject. The revised text highlights food security as a specific issue requiring timely data, emphasizing the potential for the price data to support monitoring of rapid food crisis escalation. The unique focus and value of our study are now clearly outlined in lines 61-103.

3. Complexity and Length:

• Comment: The introduction is lengthy and dense, covering multiple aspects of food insecurity, data challenges, and innovations in data methods.

• Response: We have shortened and streamlined the introduction, ensuring a tighter focus on the study’s objectives and the most relevant background information.

4. Insufficient Detail on Data Innovations:

• Comment: The introduction discusses crowdsourcing and AI but lacks specific details on their application within the study.

• Response: We have introduced more details on the crowdsourcing and AI method in lines 104-115, and have also expanded on this in the Methods section (lines 161-267) to provide a clearer understanding of the implementation of these approaches.

5. Overemphasis on General Background Information:

• Comment: The introduction includes extensive background information that could be condensed.

• Response: We have significantly condensed the introduction to focus on the information directly relevant to the study. Background information has been reduced to ensure a more direct rationale for the study's focus on validating innovative data methods.

6. Ambiguous Explanation of "Fragile Context":

• Comment: The term "fragile context" is used without a clear definition.

• Response: As mentioned in previous responses, we have clarified this term throughout the paper and de-emphasized its use in favor of more specific descriptions where possible.

7. Lack of Clear Research Questions or Objectives:

• Comment: The introduction outlines broad issues but does not clearly state the study’s research questions or objectives early enough.

• Response: We have revised the introduction to sharpen the focus, clearly stating the research objectives in lines 104-115. Some technical details have been moved to the Methods section to improve the flow and focus of the introduction.

8. Redundancy and Repetition:

• Comment: Some points, such as the limitations of traditional data collection and the potential of new data methods, are repeated.

• Response: We have reviewed the introduction for redundancy and have made edits to remove repeated points. This has resulted in a more concise and impactful presentation.

Attachment Submitted filename: Response to Reviewers.docx

# Peer review: Decision Letter 2

**DOI:** 10.1371/journal.pone.0320720.r006

**Authors:** Youssef El Khatib

25 Feb 2025

AI-imputed and crowdsourced price data show strong agreeement with traditional price surveys in data-scarce environments

PONE-D-24-22683R2

Dear Dr. Julius Adewopo,

We’re pleased to inform you that your manuscript has been judged scientifically suitable for publication and will be formally accepted for publication once it meets all outstanding technical requirements.

Within one week, you’ll receive an e-mail detailing the required amendments. When these have been addressed, you’ll receive a formal acceptance letter and your manuscript will be scheduled for publication.

An invoice will be generated when your article is formally accepted. Please note, if your institution has a publishing partnership with PLOS and your article meets the relevant criteria, all or part of your publication costs will be covered. Please make sure your user information is up-to-date by logging into Editorial Manager at Editorial Manager®  and clicking the ‘Update My Information' link at the top of the page. If you have any questions relating to publication charges, please contact our Author Billing department directly at authorbilling@plos.org.

If your institution or institutions have a press office, please notify them about your upcoming paper to help maximize its impact. If they’ll be preparing press materials, please inform our press team as soon as possible -- no later than 48 hours after receiving the formal acceptance. Your manuscript will remain under strict press embargo until 2 pm Eastern Time on the date of publication. For more information, please contact onepress@plos.org.

Kind regards,

Youssef El Khatib, Ph.D.

Academic Editor

PLOS ONE

Additional Editor Comments (optional):

Reviewers' comments:

Reviewer's Responses to Questions

**Comments to the Author**

1. If the authors have adequately addressed your comments raised in a previous round of review and you feel that this manuscript is now acceptable for publication, you may indicate that here to bypass the “Comments to the Author” section, enter your conflict of interest statement in the “Confidential to Editor” section, and submit your "Accept" recommendation.

Reviewer #1: All comments have been addressed

**********

2. Is the manuscript technically sound, and do the data support the conclusions?

The manuscript must describe a technically sound piece of scientific research with data that supports the conclusions. Experiments must have been conducted rigorously, with appropriate controls, replication, and sample sizes. The conclusions must be drawn appropriately based on the data presented. 

Reviewer #1: Yes

**********

3. Has the statistical analysis been performed appropriately and rigorously? 

Reviewer #1: Yes

**********

4. Have the authors made all data underlying the findings in their manuscript fully available?

The PLOS Data policy requires authors to make all data underlying the findings described in their manuscript fully available without restriction, with rare exception (please refer to the Data Availability Statement in the manuscript PDF file). The data should be provided as part of the manuscript or its supporting information, or deposited to a public repository. For example, in addition to summary statistics, the data points behind means, medians and variance measures should be available. If there are restrictions on publicly sharing data—e.g. participant privacy or use of data from a third party—those must be specified.

Reviewer #1: Yes

**********

5. Is the manuscript presented in an intelligible fashion and written in standard English?

PLOS ONE does not copyedit accepted manuscripts, so the language in submitted articles must be clear, correct, and unambiguous. Any typographical or grammatical errors should be corrected at revision, so please note any specific errors here.

Reviewer #1: Yes

**********

6. Review Comments to the Author

Please use the space provided to explain your answers to the questions above. You may also include additional comments for the author, including concerns about dual publication, research ethics, or publication ethics. (Please upload your review as an attachment if it exceeds 20,000 characters)

Reviewer #1: (No Response)

**********

7. PLOS authors have the option to publish the peer review history of their article (what does this mean? ). If published, this will include your full peer review and any attached files.

If you choose “no”, your identity will remain anonymous but your review may still be made public.

**Do you want your identity to be public for this peer review?** For information about this choice, including consent withdrawal, please see our Privacy Policy .

Reviewer #1: No

**********

# Editor report: Acceptance letter

**DOI:** 10.1371/journal.pone.0320720.r007

**Authors:** Youssef El Khatib

PONE-D-24-22683R2

PLOS ONE

Dear Dr. Adewopo,

I'm pleased to inform you that your manuscript has been deemed suitable for publication in PLOS ONE. Congratulations! Your manuscript is now being handed over to our production team.

At this stage, our production department will prepare your paper for publication. This includes ensuring the following:

* All references, tables, and figures are properly cited

* All relevant supporting information is included in the manuscript submission,

* There are no issues that prevent the paper from being properly typeset

If revisions are needed, the production department will contact you directly to resolve them. If no revisions are needed, you will receive an email when the publication date has been set. At this time, we do not offer pre-publication proofs to authors during production of the accepted work. Please keep in mind that we are working through a large volume of accepted articles, so please give us a few weeks to review your paper and let you know the next and final steps.

Lastly, if your institution or institutions have a press office, please let them know about your upcoming paper now to help maximize its impact. If they'll be preparing press materials, please inform our press team within the next 48 hours. Your manuscript will remain under strict press embargo until 2 pm Eastern Time on the date of publication. For more information, please contact onepress@plos.org.

If we can help with anything else, please email us at customercare@plos.org.

Thank you for submitting your work to PLOS ONE and supporting open access.

Kind regards,

PLOS ONE Editorial Office Staff

on behalf of

Prof. Youssef El Khatib

Academic Editor

PLOS ONE
